# Agricultural and geographic factors shaped the North American 2015 highly pathogenic avian influenza H5N2 outbreak

**DOI:** 10.1371/journal.ppat.1007857

**Published:** 2020-01-21

**Authors:** Joseph T. Hicks, Dong-Hun Lee, Venkata R. Duvvuri, Mia Kim Torchetti, David E. Swayne, Justin Bahl

**Affiliations:** 1 Center for Ecology of Infectious Diseases, Department of Infectious Diseases, Department of Ecology and Biostatistics, Institute of Bioinformatics, University of Georgia, Athens, Georgia, United States of America; 2 Department of Pathobiology and Veterinary Science, the University of Connecticut, Storrs, Connecticut, United States of America; 3 U.S. Department of Agriculture, Ames, Iowa, United States of America; 4 Exotic and Emerging Avian Viral Diseases Research Unit, U.S. National Poultry Research Center, Agricultural Research Service, U.S. Department of Agriculture, Athens, Georgia, United States of America; 5 Duke-NUS Graduate Medical School, Singapore; John E Fogarty International Center, UNITED STATES

## Abstract

The 2014–2015 highly pathogenic avian influenza (HPAI) H5NX outbreak represents the largest and most expensive HPAI outbreak in the United States to date. Despite extensive traditional and molecular epidemiological studies, factors associated with the spread of HPAI among midwestern poultry premises remain unclear. To better understand the dynamics of this outbreak, 182 full genome HPAI H5N2 sequences isolated from commercial layer chicken and turkey production premises were analyzed using evolutionary models able to accommodate epidemiological and geographic information. Epidemiological compartmental models embedded in a phylogenetic framework provided evidence that poultry type acted as a barrier to the transmission of virus among midwestern poultry farms. Furthermore, after initial introduction, the propagation of HPAI cases was self-sustainable within the commercial poultry industries. Discrete trait diffusion models indicated that within state viral transitions occurred more frequently than inter-state transitions. Distance and sample size were very strongly supported as associated with viral transition between county groups (Bayes Factor > 30.0). Together these findings indicate that the different types of midwestern poultry industries were not a single homogenous population, but rather, the outbreak was shaped by poultry industries and geographic factors.

## Author summary

The highly pathogenic avian influenza outbreak among poultry farms in the midwestern United States appears to be influenced by agricultural and geographic factors. After initial introduction of the virus into the poultry industries, no further introductions (such as from a wild bird reservoir) were necessary to explain the continuation of the outbreak from March to June 2015. Additionally, evidence suggests that proximity increases the chances of viral movement between two locations. While many theories have been proposed to explain the transmission of virus among poultry farms, presented evidence suggests human-mediated viral transportation played a key role in the spread of the highly pathogenic H5N2 outbreak in North America.

## Introduction

In 2014, a novel reassortant highly pathogenic avian influenza (HPAI) H5N8 virus of the hemagglutinin (HA) clade 2.3.4.4 was identified in South Korean poultry and wild birds and quickly spread to other Asian countries and Europe [1–3]. By the end of 2014, both the Eurasian H5N8 virus and its North American H5N2 reassortant were reported in western Canada and the United States [4–6]. The ensuing 2014–2015 North American HPAI outbreak marked the largest and most expensive HPAI outbreak in the United States to date [7]. In late November 2014, commercial poultry flocks in British Columbia, Canada were reported to be infected with the novel reassortant HPAI H5N2 [5], soon followed by HPAI H5N8 isolation within wild birds in the United States Pacific Northwest [4]. Over the next several months, sporadic infections arose in wild and domestic birds, including both commercial production and backyard poultry operations. In March 2015, a drastic increase of HPAI H5N2 cases was observed within domestic poultry in the Midwestern United States. By the end of the outbreak in June 2015, over 50.4 million poultry died or were culled due to the outbreak, costing the US government over $850 million, the poultry industries an estimated $700 million to $1 billion and had a negative $3.3 billion impact on the economy [7–9].

Risk factors that explain the continued transmission of HPAI between domestic poultry facilities remain unclear. For instance, previous analyses have provided conflicting evidence as to the role of wild birds in the propagation of the outbreak within the midwestern poultry industries. Despite frequent reports of wild birds on the grounds and within barns of HPAI-positive turkey premises [10], a case-control study found no significant difference in exposure to wild birds between positive turkey premises and matched controls [11]. Similarly, one phylodynamic analysis found no evidence of continued HPAI introductions into the midwestern poultry industries [12], but others have suggested multiple introductions [13,14]. Geographic and environmental variables, such as human population, agricultural, climatological, and ecological measures, may help explain farm-to-farm transmission observed within the poultry industries. For example, proximity between midwestern poultry premises has been implicated as an important risk factor for HPAI infection [11,13]. Although it has been suggested that poultry production type did not affect outbreak transmission [12], this has not been formally tested. Despite extensive molecular epidemiological studies, such environmental and ecological covariates of viral spread during this outbreak have not been formally investigated while accounting for epidemiological linkage.

Direct epidemiological links between most poultry premises are difficult to establish with traditional epidemiological approaches [15], limiting the ability to investigate risk factors that facilitated HPAI transmission among poultry farms. The incorporation of pathogen genetic sequence data into epidemiological investigations can elucidate network connections between infectious entities, be that individual hosts or populations, such as poultry farms. One approach is viral phylodynamic modeling, ie. the integration of epidemiological and evolutionary models to explore viral ecological dynamics. Based on the assumption that viral epidemiology and evolution occur on the same time scale, viral phylodynamic modeling can reveal underlying epidemiological patterns. Recent incorporation of generalized linear models (GLM), a family of commonly used regression methods, into Bayesian phylogenetic frameworks have enabled investigation into the impact of ecological factors on the diffusion of viral pathogens among discrete geographic regions [16,17]. This is in contrast to continuous trait phylogeographic modeling, which can also model environmental impacts on viral spread, given location data is continuously distributed (i.e., latitude and longitude coordinates) [18–20]. Through such approaches, factors associated with HPAI movement within United States poultry industries might be identified, informing future control efforts.

In this study, we integrated epidemiological and ecological parameters with genomic sequence data collected contemporaneously with the midwestern poultry industries HPAI outbreak to formally test outstanding hypotheses. Whole genome HPAI H5N2 sequence data isolated from layer chicken and turkey premises were analyzed using evolutionary model-based techniques. First, we utilized epidemiological compartmental population models to test the importance of poultry sector divisions (i.e. layer chicken vs turkey industries) and external viral introductions from an unsampled avian population in the propagation of the outbreak. Second, we evaluated ecological predictors of geographic diffusion of virus among midwestern counties to help identify environmental and human variables associated with viral transmission. Together, these analyses use information that accumulated within the HPAI H5N2 genome during the outbreak to help decipher higher-order patterns of viral dispersal among commercial poultry farms.

## Results

### HPAI H5N2 evolution among domestic poultry

**Fig 1:**
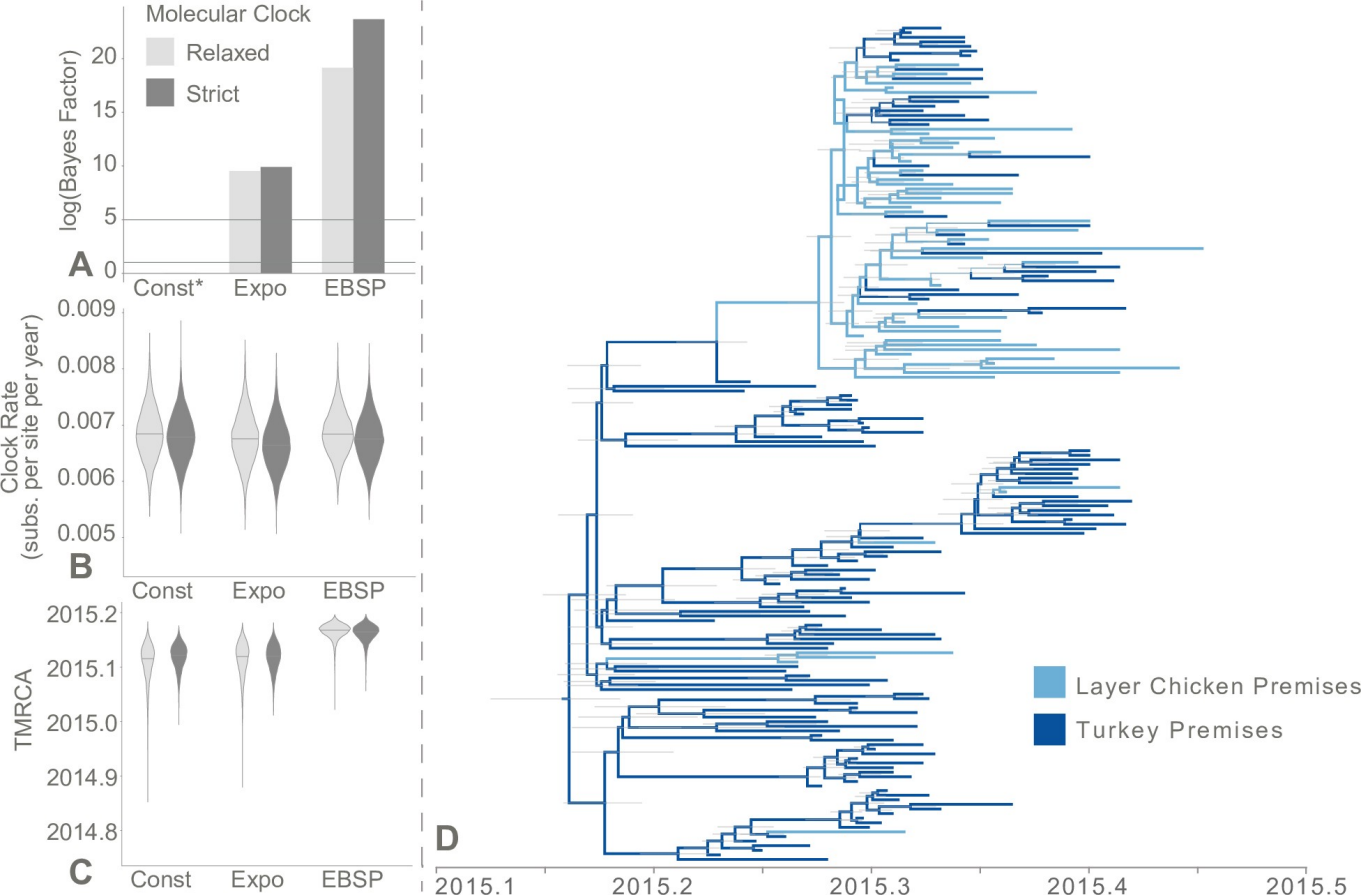
Evolutionary history of HPAI H5N2 isolated from commercial poultry premises, 2015. (A) Bayes factor (BF) tests between molecular clock and coalescent evolutionary models. For each coalescent model (exponential growth [Expo] and extended Bayesian skyline plot [EBSP]), BF was calculated using the constant coalescent model as reference (Const, indicated with asterisk) under the same molecular clock model. Two horizontal gray reference lines denote log(BF) = 1 and log(BF) = 5, which represent support and very strong support, respectively, for improved fit over the reference. (B) Molecular clock rate (substitutions per site per year) comparison between molecular clock and coalescent evolutionary models. (C) The estimated time of the most recent common ancestor (TMRCA; decimal year) compared between molecular clock and coalescent evolutionary models. (D) Maximum clade credibility tree representing the ancestral reconstruction of poultry industry (layer chicken vs. turkey) across the evolutionary history of the outbreak. The ancestral reconstruction assumed an EBSP coalescent and strict molecular clock evolutionary model. Tree branches are colored based on the most probable poultry industry of the descendant node. Thin gray node bars represent the 95% highest posterior density (HPD) of the node height (i.e., the time at which that ancestor is estimated to have existed).

182 full genome HPAI H5N2 genetic sequences, each representing a single commercial poultry farm operation across 49 counties in six states (Iowa, Minnesota, Nebraska, North Dakota, South Dakota, and Wisconsin), were included in the present analysis. The sequences were isolated from samples collected between March 25 and June 15, 2015 from positive turkey premises (72.5%) and layer chicken farms (27.5%). Based on the visual comparison of maximum likelihood phylogenetic trees of each gene segment and a recombination evaluation of the concatenated sequence data set using RDP4 [21], no evidence of reassortment was present within the concatenated whole genome sequence data set. A root-to-tip regression based on a preliminary maximum likelihood tree revealed the presence of a temporal signal within the sequence data set (*R* = 0.72, *x*-intercept = 2015.03; S1 Fig). Two molecular clock assumptions and three “traditional” coalescent models (i.e., constant population, exponential growth, and extended Bayesian skyline plot [EBSP]) were compared with marginal likelihood estimation (MLE) to evaluate the underlying population and evolutionary dynamics of the 2015 HPAI outbreak. The flexible EBSP coalescent with a strict molecular clock assumption had the best fit for the included sequence data (log(BF) = 4.45, compared with the next best fitting model, EBSP coalescent with a relaxed molecular clock; Fig 1A). Under the strict molecular clock and EBSP coalescent model, the estimated TMRCA was March 1, 2015 (95% HPD: February 16 to March 10, 2015; Fig 1C).

### HPAI H5N2 host dispersion and population dynamics

To explore the extent of viral dispersal between poultry industries, multiple phylogeographic methods were performed: the structured coalescent, the discrete trait diffusion model, and epidemiologic compartmental model-based coalescent. Each of these methods estimate a different approximation for the dispersal of virus between populations. The structured coalescent treats layer chicken premises and turkey premises as separate population demes between which virus was allowed to “migrate,” and thus estimates a *migration rate* between the two demes. In contrast, discrete trait diffusion models treat the trait of interest (here, poultry industry) as a characteristic that evolves over time, inferring a *transition rate*, analogous to a nucleotide substitution model. Finally, compartmental models enable the calculation of *transmission rates* between the two poultry compartments. Although all approximate the amount of viral dispersal among the poultry industries, each measure is calculated differently with unique assumptions and so are referred to by a particular term. All methods estimated that viral dispersal from layer chicken premises to turkey premises occurred more frequently than from turkey premises to layer chicken (S1 Table). In the structured coalescent, the migration rate from layer chicken to turkey premises was much greater than the reverse (migration rate from chickens to turkeys: 12.6, 95% HPD: 6.2–18.7; migration rate from turkeys to chickens: 0.7, 95% HPD: 0.00001–2.2). The transition rates between the poultry industries estimated from the discrete trait diffusion model were much more similar to each other (transition rate from chickens to turkeys: 1.4, 95% HPD: 0.04–3.9; transition rate from turkeys to chickens: 0.3, 95% HPD: 0.003–0.9). These models suggest the dispersion of virus between poultry industries was not symmetrical, potentially indicating poultry type played a role in the outbreak dynamics.

**Fig 2:**
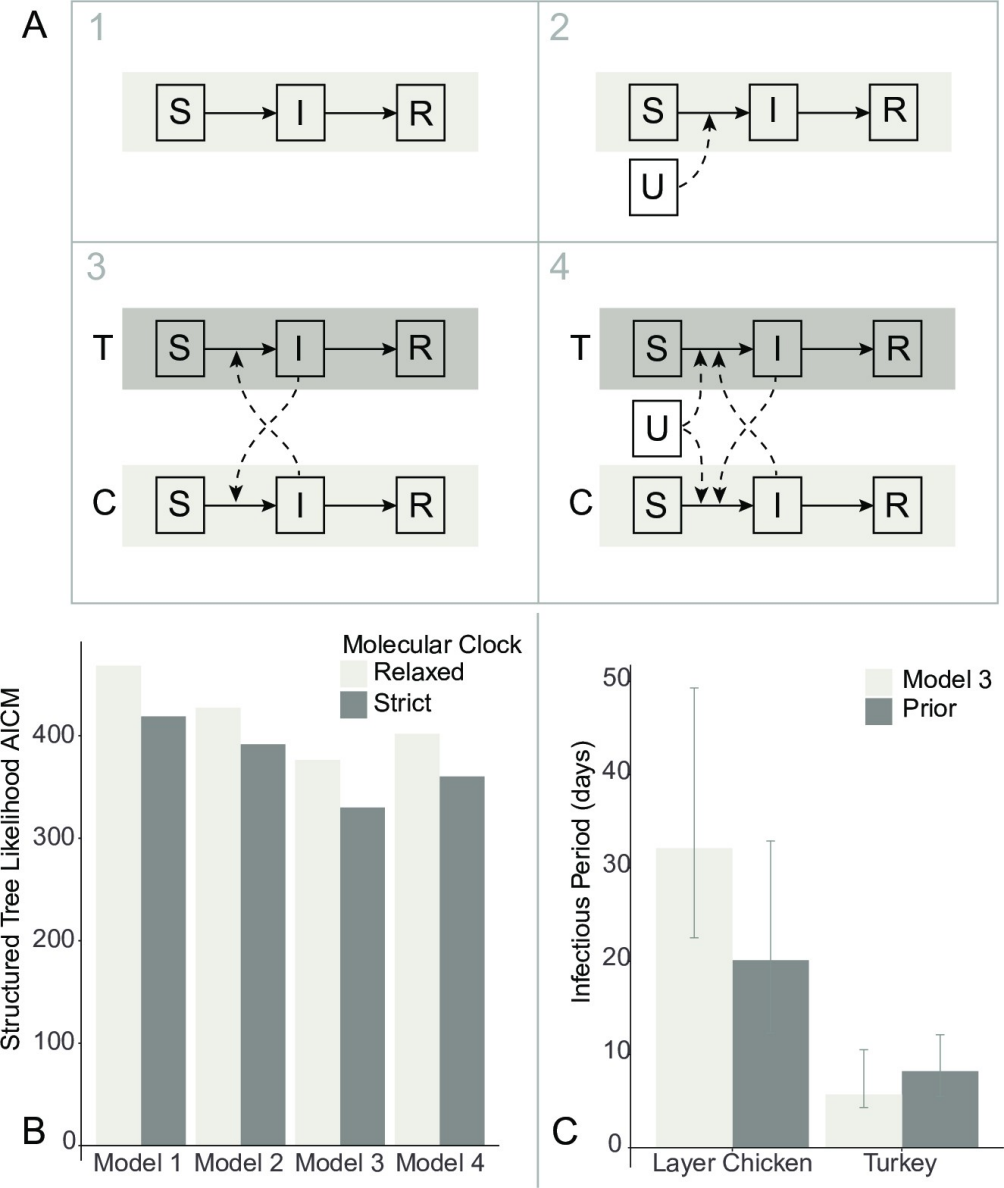
Comparison of hypothesized HPAI H5N2 epidemiological compartmental models. (A) Each compartmental model represents a Susceptible-Infectious-Removed (SIR) model with varied population heterogeneity: 1) a single, closed, homogenous population, 2) a single, homogenous population with a continual external source of virus (U), 3) a closed population, stratified by poultry system (turkeys (T) and layer chickens (C)), and 4) the stratified population with a continual external source of virus. (B) Compartmental model fit for the midwestern highly pathogenic avian influenza (HPAI) H5N2 outbreak, 2015. Akaike’s information criteria for Markov chain Monte Carlo (AICM) calculated based on the posterior distribution of the structured tree likelihood was used to evaluate the relative model fit for the four assessed compartmental models under differing molecular clock assumptions. Under both molecular clocks, Model 3 provided the best model fit. (C) Estimated infectious period of layer chicken and turkey farms during the 2015 midwestern highly pathogenic avian influenza (HPAI) H5N2 outbreak. During model specification, an informative prior was provided for the Bayesian process. This prior probability distribution was based on the reported average time from HPAI confirmation to depopulation plus 5 days to allow for delay between infection and HPAI confirmation. Model 3 estimated the infectious period for layer chickens to be longer than expected given the prior information.

To formally test this hypothesis, we used epidemiological compartmental model equations to describe the coalescent process [22]. Four competing scenarios were constructed (Fig 2A, S1 Text). Models 1 and 2 described a homogenous poultry population that differed by the presence of a continuous external viral source in Model 2. In contrast, Models 3 and 4 described a host population stratified by poultry production system, again differing based on an external viral source in Model 4. It should be noted that due to the sampling scheme of genetic sequences (one HPAI whole genome sequence per infected premises), the epidemiologic unit of interest was the premises (or farm), and not the individual bird. That is, findings of the compartmental models should be interpreted on the farm-to-farm scale, not the dynamics of transmission between individual birds. Akaike’s information criteria for Markov chain Monte Carlo (AICM) calculated from the posterior sample of structured tree likelihood estimates revealed that Model 3 provided the best fit for the data under both strict and relaxed molecular clock assumptions (AICM under strict clock = 330.1; under relaxed clock = 376.3; Fig 2B, S2 Table). This suggests the midwestern portion of the 2015 HPAI outbreak was isolated from external sources but most likely structured by poultry production system. Model 4, which includes parameters that allow for an external unsampled source, estimated an introduction rate that includes zero, equivalent to Model 3 (η_T_ 95% HPD 0.0–6.4; η_C_ 95% HPD 0.0–0.7). Estimated parameter values for each model are provided in S3 Table. Four transmission rates were estimated for Model 3 to describe the interaction between the layer chicken and turkey populations: two within-poultry system rates (β_T_ and β_C_) and two between-poultry system rates (β_TC_ and β_CT_). The model estimated the transmission rates within the turkey production system to be highest (β_T_ = 11.6, 95%HPD: 2.0–22.0), followed by transmission rates from chicken farms to turkey farms (β_CT_ = 4.9, 95% HPD: 0.6–9.6). The lowest transmission rate was estimated from turkey farms to chicken farms (β_TC_ = 0.1, 95% HPD: 0.02–0.22). This is similar to the results of the structured coalescent model and discrete trait model described above (S1 Table). Infectious period of a farm also varied substantially between the two production systems. A HPAI-positive turkey premises was estimated to remain infectious for 5.7 days (95% HPD: 4.3–10.5), whereas layer chicken premises were estimated to remain infectious for 32.1 days (95% HPD: 22.4–49.3; Fig 2C).

### Ecologic predictors of HPAI H5N2 geographic diffusion

**Fig 3:**
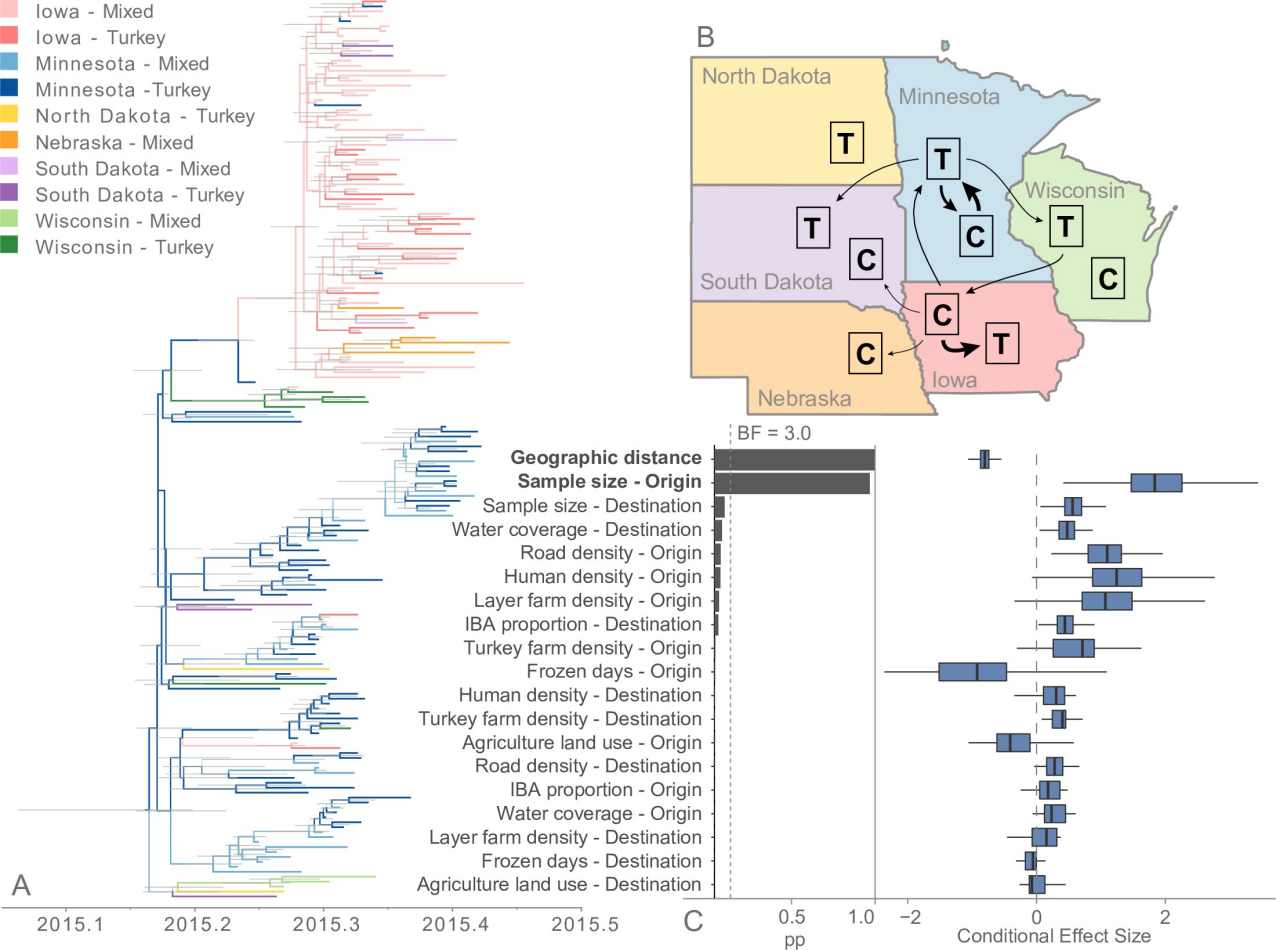
Discrete trait diffusion model of HPAI H5N2 among midwestern county groups. (A) Maximum clade credibility tree representing the ancestral reconstruction of county groups across the evolutionary history of the outbreak. The ancestral reconstruction was based on an EBSP coalescent and strict molecular clock evolutionary model. Tree branches are colored based on the most probable county group of the descendant node. Thin gray node bars represent the 95% highest posterior density (HPD) of the node height (i.e., the time at which that ancestor is estimated to have existed). (B) Diffusion rate summary among county groups. County groups were defined based on state and composition of host type within the county. Counties with only turkey cases (turkey exclusive; T) were grouped separately from counties with at least one layer chicken case (mixed poultry; C). Arrows represent transition rates with strong support (Bayes factor > 10) with arrow thickness proportional to the magnitude of transition rate. (C) Conditional effect size of environmental and geographic covariates within the generalized linear model (GLM). Conditional effect size represents the effect size of the variable coefficient given inclusion in the GLM. Supported covariates (Bayes factor > 3) are bolded. Covariates are ordered by Bayes factor. The dashed gray line represents a conditional effect size of 0, signifying little impact of the covariate on viral dispersal.

Using the posterior distribution of phylogenetic trees estimated under the EBSP coalescent and strict molecular clock assumptions, discrete trait diffusion models were estimated to describe the geographic dispersal of HPAI H5N2 throughout the midwestern United States. County of origin was used as the basis to categorize the 182 sequences. To capture both geographic and host information in the discrete trait definition, counties were grouped based on state and host composition. Counties were composed of only commercial turkey farms, only commercial layer chicken farms, or a mix of both farm types. In the final categorization, counties with only commercial layer chicken farms and a mix of farm types were combined to avoid categories with sparse sequence data, resulting in a total of 10 discrete trait categories (2 to 60 sequences per category). County groups with only turkey cases are henceforth referred to as turkey-exclusive while county groups with at least one layer chicken case are referred to as mixed poultry. The complete ancestral reconstruction of the midwestern outbreak is shown in Fig 3A. The three largest transition rates were observed between county groups within the same state, particularly Minnesota and Iowa (Fig 3B; S4 Table). The most frequent transitions occurred from Minnesota mixed poultry counties to Minnesota turkey-exclusive counties (median rate: 3.3 transitions per year; 95% HPD 0.7–6.4; BF = 490.6) and from Iowan mixed poultry counties to Iowan turkey-exclusive counties (3.3 transitions per year; 95% HPD 1.4–5.7; BF = 28,139.6). In Minnesota, the reverse rate (i.e., from turkey-exclusive counties to mixed poultry counties) was also decisively supported and had a relatively high transition rate (2.3 transitions per year; 95% HPD: 0.6–4.6; BF = 2,007.1). Three inter-state transitions were also decisively supported, but less frequent. These transitions were estimated from Iowa mixed-poultry counties to Minnesota turkey-exclusive counties (0.9 transitions per year; 95% HPD 0.2–2.2; BF = 14,068.3), from Minnesota turkey-exclusive counties to Wisconsin turkey-exclusive counties (0.74 transitions per year; 95% HPD 0.1–1.8; BF = 202.3) and from Wisconsin turkey-exclusive counties to Iowan mixed-poultry counties (0.9 transitions per year; 95% HPD 0.01–2.6; BF = 134.8). All supported transition rates (BF > 3.0) were found either within a state or between states that share borders, except for a single weakly supported rate from South Dakota turkey counties to Wisconsin mixed poultry counties (0.6 transitions per year; 95% HPD 0.0002–2.1; BF = 3.2). This suggests geographic distance influences the dispersal of HPAI H5N2 among midwestern counties.

As a secondary analysis to examine temporal patterns in viral dispersion among county groups, the discrete trait diffusion model was divided into two epochs, before and after April 10, 2015. This date represents the midpoint in time between the root of the phylogenetic tree and the last branch-point in the evolutionary history of the outbreak. Before April 10, only four transition rates were statistically supported, all of which originated from turkey-exclusive county groups, particularly in Minnesota and Wisconsin (S5 Table; S2 Fig). In contrast, after April 10, of the eight supported transition rates, six originated from mixed county groups. In the later part of the outbreak, only Iowa and Minnesota counties acted as origins of the virus to other areas. The sole transition rate supported in both phases of the outbreak was estimated from Minnesota turkey-exclusive counties to Minnesota mixed poultry counties.

The single-epoch discrete trait diffusion model was extended with a GLM that assessed the impact of distance and other environmental variables on the transition rates among the defined county groups. County characteristics for the 9 modeled variables are summarized in S6 Table. On average, county centers were 266 km apart, ranging from 30 to 862 km. HPAI-positive counties had a higher density of layer chicken farms (0.02 farms/km^2^) than turkey farms (0.004 farms/km^2^). Counties also had a broad range of human population density ranging from about 1 to 58 people/km^2^. In addition to the 9 environmental variables, sample size of the origin and destination county groups was included and compared with a GLM model that did not adjust for sample size. Only one predictor was statistically supported to be associated with diffusion of HPAI H5N2 among county groups in both GLM models (Fig 3C, S7 Table, S8 Table). In the sample size-adjusted model, distance between county group centroid was decisively supported to be negatively associated with transition between two groups (log conditional effect size = -0.8; 95% HPD -1.0, -0.6; BF = 242,228.2). In other words, viral transitions are less likely between county groups that are separated by a greater distance. Sample size of the originating county group was decisively supported in the sample-size adjusted model (log conditional effect size = 1.8; 95% HPD 0.8–3.2; BF = 783.3). While Road density of the origin county group (log conditional effect size = 1.2; 95% HPD 0.6–1.7; BF = 42.8) and proportion of the destination county group covered with water (log conditional effect size = 0.6; 95% HPD 0.2–0.9; BF = 3.9) were positively associated with viral dispersion in the non-adjusted analysis, these associations disappear with the inclusion of sample size. This suggests county group sample size partially explains viral dispersion observed in the analysis. To assess the impact of highly correlated environmental characteristics (S3 Fig), the GLM was repeated removing human density and important bird area (IBA) variables, providing similar results to the main analysis (S9 Table).

## Discussion

Our exploration of population models to describe the 2015 midwestern United States HPAI H5N2 outbreak provides evidence that upon entering the midwestern poultry industries, the outbreak was self-sustaining, requiring no further viral introductions from outside sources to explain the observed epidemiological trajectory. Furthermore, the statistical support for a stratified poultry population suggests that poultry industries should not be considered a homogenous host population for viral pathogens. It should be noted that poultry production system barriers were not the only observed factors influencing the course of the outbreak within the midwestern United States. As observed in the discrete trait diffusion models, geographic distance is associated with viral dispersion among counties, indicating viral movement is not random, but rather governed by environmental realities. Furthermore, viral dispersal patterns among both poultry production systems and geographic regions shifted over time, from an outbreak predominated by turkey premises in Minnesota to a predominance of infected layer chicken premises in multiple states.

In our analysis, the EBSP coalescent model had better support than the other traditional coalescent models in terms of model fit. This is most likely a reflection of EBSP’s flexibility, i.e. the piece-wise nature of this method, which facilitates the identification of complex population changes. Coalescent theory has been a popular technique to infer population demographics underlying viral outbreaks [23–32]. By relating effective population size to the rate at which phylogenetic lineages converge backwards in time, the coalescent has become a powerful tool to infer demographic changes in the face of incomplete sampling. Traditionally, the estimation of the coalescent process required rigid prior assumptions in the form of simplistic mathematical growth functions (e.g., constant population size, exponential growth, logistic growth, and expansion growth). To better reflect biological reality, methods have been developed that incorporate more flexibility than a one to two parameter mathematical function, such as the Bayesian skyline, extended Bayesian skyline (EBSP), skyride and skygrid plots [33–36]. For instance, the EBSP assumes demographic changes follow a smoothed, piece-wise, linear function whose change points are inferred from the sequence data [35]. To date, mathematical methods to incorporate population structure into EBSP coalescent models have not been developed even though population structure has been observed to confound coalescent estimates [37,38].

Despite the flexibility of EBSP, compartmental-based coalescent models are worth assessing as they allow for direct incorporation and hypothesis testing of specific population structures. Rather than the non-parametric, piece-wise approach of EBSP, the prior mathematical functions assumed are ordinary differential equations (ODEs) constructed from the specification of epidemiological compartmental models. It is the parameters of these ODEs that are fit during the Markov chain Monte Carlo (MCMC) process. Among the four analyzed compartmental models, we found that the closed, stratified population provided the best fit for the sequence data, suggesting layer chickens and turkeys represented two separate host populations that interacted with each other, but did not receive virus from a continuous external source. Interestingly, when only observing the single homogenous population models (Models 1 and 2), the inclusion of an external viral source (Model 2) improves model fit compared to the closed population model (Model 1). Once the population structure of poultry type is included (Models 3 and 4), the closed population model provides a better fit than that with continual viral introductions. This observation underlines the importance of including population heterogeneity within evolutionary demographic models to explain observed viral diversity and population dynamics.

To help improve the ability to estimate the remaining parameters within the compartmental model, expected prior distributions for the infectious period of affected premises were specified based on reported USDA data [7]. Despite the informative assumption, the infectious period of layer chicken farms was estimated to be longer than expected. In our model, we assumed a 5-day period between the onset of infectivity of the farm and reporting of HPAI infection. Delays in the identification and/or reporting of HPAI infection could result in infectious periods that begin well before the assumed 5 days. Continued infectivity beyond the completion of flock depopulation is another likely contributor to prolonged infectious periods. Although commercial poultry depopulation occurred on average 6.4 days after National Veterinary Services Laboratory (NVSL) HPAI confirmation, premises were not considered to be virus-free until, on average, 87.7 days following confirmation [7]. In either case, our models suggest layer chicken farms remained infectious for much longer than turkey farms, potentially explaining why the transmission rate from chicken farms to turkey farms was higher than its counterpart. In fact, regardless of the model (i.e., structured coalescent, discrete trait diffusion model, or compartmental model), layer chicken farms played a more central role to viral transmission than turkey farms during the outbreak. This may seem contradictory to experimental evidence that demonstrated the HPAI H5N2 virus had longer mean death times in turkeys (5–6 days) compared to chickens (2–3 days [39]. However, such experimental infections only describe transmission information on the individual bird scale, rather than the farm-to-farm transmission scale captured in this analysis. Although it may be that individual turkeys survive longer, in practice turkey premises were quicker to be depopulated, resulting in a shorter farm-level infectious period compared to chicken farms. Because intervention (i.e. depopulation) was performed on the farm level, individual-level infectious periods alone are not adequate to describe the overall observed outbreak dynamics.

The implementation of a GLM into a Bayesian discrete trait analysis has been previously applied to HPAI in China [40] and Egypt [41], providing evidence that environmental, agricultural and anthropogenic factors influence viral movement. Due to differences in social, governmental and agricultural systems, the generalization of these previous GLM results to other countries may not be appropriate. When such phylogeographic methods are applied to the North American HPAI outbreak among commercial poultry farms, distance is estimated to be a key factor that influenced the geographic spread of HPAI H5N2 among midwestern counties in the spring of 2015. A recent spatial modeling analysis revealed that HPAI spread among Minnesota poultry premises was heavily distance-dependent during the 2015 outbreak [13]. Our results support this claim by providing evidence that the frequency of viral transition between two locations increases as the distance between locations decreases. Risk of infection due to proximity can also be observed in our discrete trait diffusion model in which within-state HPAI spread was much more frequent than inter-state spread. HPAI movement between states may explain Bonney, et al.’s finding that distance-independent transmissions significantly improved the fit of their transmission kernel model [13].

Although the causal relationship between the supported covariates and viral dispersal cannot be determined from our analysis, the statistical support for road density within the GLM may provide evidence for the relative importance of anthropogenic movement of virus. The significance of road density is called into question, though, as it loses statistical support following adjustment for sample size. While the inclusion of sample size as a covariate has been argued as a means to assess the presence of sampling bias in phylogeographic GLM analyses [42], there is evidence to suggest that such practices hinder coefficient estimation [43]. Furthermore, the data analyzed in the present study were not subject to differential sampling efforts; rather, the geographic and host distribution of sequences reflect the outbreak distribution, thanks to intensive surveillance and sequencing efforts among commercial poultry premises during the outbreak. High road density may correlate with better logistic connectivity between farms, increasing the likelihood that an infected premises will export virus to nearby farms and counties. Road density has been associated with HPAI H5N1 outbreaks in Bangladesh [44], Thailand [45], Romania [46], Indonesia [47,48], and Nigeria [49], although high road density in these countries may reflect greater human population density and, therefore, a higher likelihood of case detection [50]. Intensive commercial poultry surveillance during the 2015 outbreak and the lack of support for human population density as a covariate within our model suggest that the statistical support for road density in the dispersal of HPAI among midwestern counties may not merely be an artifact of greater case detection in densely populated counties. A more exact measure of logistic connectivity between premises is the calculated driving time between infected poultry premises. Unfortunately, due to the lack of exact coordinates of infected premises, this covariate could not be included in the analysis. Alternatively, circuit theory can be used to compute environmental distances between discrete locations, which takes into account inaccessible areas and multiple paths of travel, to provide a more complete measure of the connectedness of two locations [19].

In this analysis, we provide limited evidence of the association between HPAI dispersal and the proportion of a destination county group covered by surface water. In other words, counties with a larger proportion of surface water received virus more frequently compared to those with less surface water. Surface water resources have been associated with HPAI dispersal and prevalence in China and may signify movement of virus by migrating waterfowl that stopover in lakes, rivers and wetlands [40,51]. In our analysis, this variable was only weakly supported, had a relatively small effect size and was not supported once the model was adjusted for county group sample size. Additionally, other variables that represent potential migratory stopover habitats, such as Important Bird Areas and agricultural land, were not supported within the model, suggesting that if wild birds contributed to HPAI dispersal within the Midwest, their role was limited. This further supports previous studies, which indicated that the midwestern portion of the outbreak was driven by inter-farm transmission [11,12,14]. Several mechanisms have been proposed to explain HPAI transmission between farms during the 2015 outbreak, including equipment sharing, personnel overlap, and aerosolization.

Due to the restricted number of sequences in the presented analysis, the number of variables and demographic scenarios that could be modelled was limited. This also affected the resolution of the geographic covariates that could be included within the GLM. In addition, precise locations of commercial premises were not available, preventing the ability to perform a continuous trait diffusion analysis. Rather, the most granular level of geographic information was county-level. Ideally, the environmental and agricultural characteristics of each individual farm or county would be evaluated as predictor for HPAI spread; however, the individual transition rates between 182 farms or even 49 counties would be impossible to accurately estimate from the 182 sequences of this dataset. For this reason, sequences were categorized into 10 county groups, resulting in a manageable transition rate matrix as well as permitting the summarization of environmental characteristics across a few counties rather than across an entire state. It should be noted that the grouping of counties by state and poultry type composition may have influenced both discrete trait and GLM analyses. While state boundaries are somewhat arbitrary, states are a practical means of categorizing agricultural entities as many agricultural policies are enacted at the state level. The division of counties by those with only infected turkey premises versus those with any infected chicken premises may have introduced bias into the analyses. For example, connections between the turkey and mixed poultry county groups may solely be due to viral dispersal among turkey premises. Categorization also assumes that categories are meaningfully different from each other. Similarity among county groups of the included environmental factors could make it difficult to detect associations with viral dispersal. Another potential limitation of phylogeographic models is sampling bias introduced by differential sampling effort. The influence of sampling bias in our GLM analysis is likely mitigated for two reasons. First, as mentioned, due to intense surveillance activities during the outbreak and viral sequencing of almost all infected premises, the sampling proportions across counties likely reflect true patterns in viral distribution. Second, when sampling disparities were adjusted for in the GLM, the covariate with the strongest support remained statistically significant. A GLM analysis based on a structured coalescent model may help resolve impacts of sampling on predictors of viral dispersal by incorporating demographic dynamics and allowing dispersal rates to vary with time [52].

Despite potential limitations, our results present several implications for future HPAI surveillance and control in the United States. While wild birds may provide a means of viral dispersal across large distances and initial introduction into an area, evidence suggests the HPAI outbreak within the midwestern poultry industries could be maintained without continued introductions. In this sense, in-place biosecurity efforts may have been enough to prevent continued viral introductions from outside sources (including wild birds, backyard poultry flocks, or long-distance movement from other geographic regions), but were ineffective against local farm-to-farm transmission. For instance, it has been suggested that biosecurity factors could explain the lack of HPAI cases within the broiler chicken industry in the Midwest [53]. A better understanding of how HPAI-positive farms are logistically connected would greatly aid surveillance and control efforts. With the knowledge of how these farms share personnel and equipment, future outbreaks could be contained by disruption of the transportation network.

## Methods

### Dataset

Whole genome HPAI H5N2 sequences collected, isolated and sequenced by the United States Department of Agriculture (USDA) during the 2014–2015 North American HPAI outbreak served as the basis for the analyzed data set. Full description of their collection and sequencing has been reported elsewhere [14]. A subset of this sequence data was selected to better investigate the farm-to-farm transmission dynamics of the midwestern portion of the HPAI H5N2 outbreak. This subset was defined by the following inclusion criteria: 1) sequences isolated from commercial domestic poultry samples and 2) membership of the sequence in a phylogenetically distinct group, as determined by maximum likelihood estimation by Lee, et al [14]. These viruses represented midwestern HPAI-positive poultry premises from the latter part of the outbreak, which was defined by a rapid increase in incidence within the midwestern poultry industries. As within-farm epidemiological dynamics were not of interest in this analysis, only one viral sequence per positive poultry premises was included. Viruses isolated from backyard poultry operations and wild birds were not included due to the incongruency in surveillance and sampling between these populations and the domestic poultry industries. A full list of the included sequence names and accession numbers are provided in S10 Table.

### Coalescent model comparison

Coalescent theory provides the statistical framework to estimate population changes over time from genetic sequence data. To investigate the population dynamics of the midwestern poultry portion of the outbreak, various coalescent population model prior assumptions were implemented and compared in BEAST2 [54]. Using ModelFinder [55] as implemented in the IQ-TREE software package (http://www.iqtree.org/), the Kimura three parameter (K3P; i.e., one transition rate and 2 transversion rates) model [56] with unequal base frequencies and a gamma distribution of rate variation among sites [57] was determined as the best fit nucleotide substitution model and was used for each BEAST2 model. Based on the maximum likelihood tree produced by IQ-TREE, a root-to-tip regression was performed using TempEst [58] as a preliminary assessment of the presence of a temporal signal within the sequence data. All coalescent models were separately estimated under both strict and lognormally distributed, uncorrelated, relaxed molecular clock assumptions. For each BEAST2 model, at least three independent MCMC runs of 50 million chain length were initiated from random starting trees. Convergence and chain stationarity was assessed in Tracer v1.5, ensuring an effective sample size (ESS) > 200 for each estimated parameter. If ESS < 200 or stationarity had not been reached at the default 10% burn-in fraction, the discarded burn-in fraction was increased or more MCMC runs were performed. Three “traditional” coalescent models (i.e., constant population, exponential growth, and EBSP [35]) were performed to investigate demographic dynamics. The mean rates for both the strict and relaxed molecular clocks were specified as a uniform distribution from 0 to 1 with an initial value of 0.0033. For all coalescent models, the mean population size was specified as a log normal distribution with a mean in real space of 5.0 and S = 1.25. All other parameters remained set to default settings. Model fit was compared among the coalescent and molecular clock models with path sampling to calculate the marginal likelihood estimate (MLE) [59]. Estimating the marginal likelihood enables the calculation of a Bayes Factor (BF), which is a ratio of two marginal likelihoods. A log(BF) > 5 indicates very strong statistical support for one model over the other [60]. Viral dispersion between poultry industries (layer chicken vs. turkey) was initially estimated with a simple discrete trait diffusion model [61,62] as well as a structured coalescent [29]. The EBSP coalescent model was used as the tree prior for the discrete trait diffusion model. Both viral dispersion models were performed under both strict and relaxed molecular clock, as above.

A recently developed structured coalescent-based BEAST2 package (PhyDyn) was used to investigate more complex pathogen population scenarios by specifying epidemiological compartmental models [22]. Four alternative compartmental models were assessed to investigate the presence of population structure by poultry type (layer chicken vs. turkey) and continual viral introductions from an unknown source population. Each compartmental model was a Susceptible-Infectious-Removed (SIR) model with varied population heterogeneity (Fig 2A): 1) a single, closed, homogenous population, 2) a closed population, stratified by poultry system, 3) a single, homogenous population with a continual external source of virus, and 4) a stratified population with a continual external source of virus. By including models with an external viral source, the models test whether repeated introductions of HPAI from wild birds, backyard poultry, or undetected HPAI-positive premises had a significant role during the outbreak among commercial poultry. Prior settings for the PhyDyn coalescent models are provided in S11 Table. Since marginal likelihood estimation via path sampling has not yet been developed for the PhyDyn package, Akaike Information Criterion for MCMC (AICM) [63] was used to assess model fit and was calculated from the posterior MCMC sample of the structured tree likelihood with the R package, aicm (https://rdrr.io/cran/geiger/man/aicm.html).

### Discrete trait diffusion models

To estimate the impact of environmental variables on the geographic diffusion of HPAI between midwestern counties, a discrete trait diffusion model was constructed and further extended with a generalized linear model (GLM) in BEAST v1.10 [64]. Discrete trait diffusion models are a phylogeographic technique in which each analyzed genetic sequence is assigned an observed characteristic trait that is assumed to have changed across the viral evolutionary history in a continuous time Markov chain process [61]. Transition rates among these observed traits can then be inferred. In this analysis, the discrete character trait definition was based on the United States county in which the HPAI-positive poultry premises was located. Counties were then categorized by state and whether the county’s sequences exclusively originated from turkey production premises. This method of categorization was employed to group sequences by both geographic region and poultry industry. Because the majority of sequences originated from turkey premises, counties with only infected turkey premises were more common than counties with infections of only layer chicken premises. Therefore, layer chicken-exclusive and mixed poultry counties were combined to avoid categories with sparse sequence data as well as limiting the total number of discrete categories in the face of a low overall sample size. In contrast to the above discrete trait model that solely tested viral dispersion between the two poultry industries, this model enables viral dispersion among geographic regions to be estimated. Because the midwestern outbreak appears dominated by two waves of viral proliferation, a secondary analysis was performed in which temporal patterns of HPAI geographic diffusion were assessed by defining two epochs of viral movement during the outbreak [65]. The epoch change point was determined as the halfway point between the tree root and the youngest internal node based on the annotated phylogenetic tree estimated under strict molecular clock and EBSP coalescent assumptions.

The geographic discrete trait diffusion model was extended with a GLM to assess the impact of environmental covariates on the viral transition rates among county categories. In this approach, viral diffusion rates among discrete geographic regions act as the outcome to a log-linear combination of environmental variables, regression coefficients and indicator variables [17]. Environmental and anthropogenic variables were selected based on previous indication of their importance to avian influenza risk [50]. Layer chicken farm density and turkey farm density were calculated from USDA 2012 census data (https://quickstats.nass.usda.gov/) divided by the land area of the county group. Human population density and proportion of county covered in water was obtained from United States census data (https://factfinder.census.gov/). The remaining variables were summarized per county group using ArcGIS Pro. Geographic distance was calculated as the linear distance between county group centroid. Road density was estimated as the total length of road per county divided by the total county group area. Proportion of county designated as an important bird area (IBA) was calculated using the publicly available Audubon Important Bird Areas and Conservation Priorities data [66]. Proportion of the county group used for agriculture (i.e., covered by pasture, hay or cultivated crops) was obtained from the United States Geological Survey National Land Cover Database created in 2011 and amended in 2014 [67]. The number of frozen days was calculated from daily freeze-thaw satellite data from March 1 to June 15, 2015 [68,69]. A frozen day was defined as a day in which more than half of the county group area had a temperature measured as below 0 C. All covariate measures were log-transformed and standardized before inclusion in the GLM. Two secondary GLM analyses were performed to assess the influence of collinearity and sampling bias on the observed results. First, collinearity of included covariates was assessed with the Pearson correlation test, and the GLM was performed with highly correlated variables (*R*^*2*^ > 0.85) removed. Second, sample size of both the originating and destination county group was included in the GLM. This attempts to control for disparities in sampling magnitude among the discrete trait categories [42].

The discrete trait diffusion models were applied to the empirical distribution of phylogenetic trees from the best fitting evolutionary model. For each diffusion model, three independent MCMC runs of 1 million steps in length were performed, sampling every 100 steps. Convergence was assessed in Tracer v1.5, ensuring ESS > 200 for each estimated parameter. Removing the first 10% of each run as burn-in and re-sampling every 300 steps, log and tree files were combined using LogCombiner in the BEAST v1.10 software suite. Statistical support for transition rates in the discrete trait diffusion model and the covariate coefficients of the GLM were inferred using Bayesian stochastic search variable selection (BSSVS). Briefly, for each estimated parameter, an indicator variable (I) is stochastically turned on (I = 1) or off (I = 0) at each step of the MCMC [16,61]. The posterior distribution of indicator values can be used to calculate a Bayes factor (BF), indicating the level of statistical support for the inclusion of that parameter in the model. BF support was defined in the following categories: no support (BF < 3.0), substantial support (3.0 ≤ BF < 10.0), strong support (10.0 ≤ BF < 30.0), very strong support (30.0 ≤ BF < 100.0), and decisive support (BF ≥ 100.0). Median transition rates, median conditional coefficients, 95% highest posterior density (HPD) and BF were calculated using personalized Python scripts that incorporate functions from the PyMC3 module [70]. BEAST code and customized analytical scripts have been uploaded to a public GitHub depository, https://github.com/jt-hicks/Hicks-et-al_2019_HPH5.

## Supporting information

S1 Text Mathematical equations that parameterize the four investigated epidemiologic compartmental models. (PDF)

S1 Table Estimates of viral transmission between poultry industries during the 2015 highly pathogenic avian influenza virus H5N2 outbreak within the midwestern United States. (PDF)

S2 Table Akaike’s information criteria for Markov chain Monte Carlo (AICM) for the epidemiological compartment-based coalescent models. (PDF)

S3 Table Parameter estimates of the Bayesian framework epidemiologic compartmental models. Posterior probability, likelihood, structured tree (ST) likelihood, and prior probability are provided. Each model was performed under two different molecular clock assumptions (lognormal relaxed and strict). Median values with corresponding 95% highest posterior density (HPD) are displayed. Refer to Appendix B: Text S3.1 for parameter definitions.
(PDF)

S4 Table Discrete trait diffusion matrix of the midwestern highly pathogenic avian influenza (HPAI) H5N2 outbreak, 2015. Median rates and associated 95% highest posterior density intervals (in brackets) are presented in each cell. The diffusion model is asymmetrical, and therefore, rates have directionality from a source county group (indicated on the left) to a sink county group (indicated across the top). County groups were defined by state (IA—Iowa, MN—Minnesota, ND—North Dakota, NE—Nebraska, SD—South Dakota, WI—Wisconsin) and composition of poultry type (T—turkey exclusive, CM—layer chicken exclusive and mixed poultry). Rates are colored by the level of Bayes factor support: no support (BF < 3.0), substantial support (3.0 ≤ BF < 10.0), strong support (10.0 ≤ BF < 30.0), very strong support (30.0 ≤ BF < 100.0), and decisive support (BF ≥ 100.0).
(PDF)

S5 Table Discrete trait diffusion matrix, by epoch, of the midwestern highly pathogenic avian influenza (HPAI) H5N2 outbreak, 2015. Two diffusion matrices were estimated: before and after April 10, 2015. Median rates and associated 95% highest posterior density intervals (in brackets) are presented in each cell. The diffusion model is asymmetrical, and therefore, rates have directionality from a source county group (indicated on the left) to a sink county group (indicated across the top). County groups were defined by state (IA—Iowa, MN—Minnesota, ND—North Dakota, NE—Nebraska, SD—South Dakota, WI—Wisconsin) and composition of poultry type (T—turkey exclusive, CM—layer chicken exclusive and mixed poultry). Rates are colored by the level of Bayes factor support: no support (BF < 3.0), substantial support (3.0 ≤ BF < 10.0), strong support (10.0 ≤ BF < 30.0), very strong support (30.0 ≤ BF < 100.0), and decisive support (BF ≥ 100.0).
(PDF)

S6 Table Demographic and geographic characteristics of the 49 United States counties with HPAI-positive commercial poultry premises during the H5N2 outbreak, 2015. (PDF)

S7 Table Generalized linear model (GLM) conditional effect sizes and statistical support for agricultural and geographic covariates of the dispersal of highly pathogenic avian influenza (HPAI) H5N2 among midwestern county groups. Sample sizes of origin and destination county groups have been included to estimate the influence of sampling bias. Conditional effect size and 95% highest posterior density (HPD) were calculated based on the estimated GLM coefficients given the Bayesian stochastic search variable selection (BSSVS) indicator = 1. The posterior probability (PP) refers to the proportion of Markov chain Monte Carlo (MCMC) samples in which the BSSVS indicator = 1. Bayes factor (BF) > 3.0 indicates statistical support for the inclusion of the covariate within the GLM.
(PDF)

S8 Table Generalized linear model (GLM) conditional effect sizes and statistical support for agricultural and geographic covariates of the dispersal of highly pathogenic avian influenza (HPAI) H5N2 among midwestern county groups. Conditional effect size and 95% highest posterior density (HPD) were calculated based on the estimated GLM coefficients given the Bayesian stochastic search variable selection (BSSVS) indicator = 1. The posterior probability (PP) refers to the proportion of Markov chain Monte Carlo (MCMC) samples in which the BSSVS indicator = 1. Bayes factor (BF) > 3.0 indicates statistical support for the inclusion of the covariate within the GLM.
(PDF)

S9 Table Median conditional effect sizes of environmental and geographic covariates within the generalized linear model (GLM), with highly correlated variables removed. Conditional effect size represents the effect size of the variable coefficient given inclusion in the GLM. Supported covariates (Bayes factor > 3) are bolded. Covariates are ordered by Bayes factor.
(PDF)

S10 Table Accession number and names of 182 included HPAI H5N2 full genome sequences. (PDF)

S11 Table Prior settings of the compartmental coalescent models. All parameters were specified to be log normally distributed with a lower bound of 0. Mean (M) and standard deviation (S) are specified in the log distributed space. Initial values are in real space.
(PDF)

S1 Fig Root-to-tip regression of 182 included HPAI H5N2 full genome sequences. (PNG)

S2 Fig Diffusion rate summary among county groups, by epoch. Epochs were defined as before (A) and after (B) April 10, 2015. Counties with only turkey cases (turkey exclusive; T) were grouped separately from counties with at least one layer chicken case (mixed poultry; C). Arrows represent transition rates with strong support (Bayes factor > 10) with arrow thickness proportional to the magnitude of transition rate.
(PDF)

S3 Fig Correlation matrix of investigated environmental covariates. Variables have been log-transformed and standardized. Lower half of the matrix represents scatterplots between pairs of variables. Upper half of the matrix communicate the Pearson correlation coefficient. Level of statistical significance (p = 0.05, p = 0.01, p = 0.001) is denoted by asterisks (*, **, and ***, respectively).
(PDF)
