# Supplementary material for: Agricultural and geographic factors shaped the North American 2015 highly pathogenic avian influenza H5N2 outbreak
Source: PLoS Pathog. 2020 Jan 21;16(1):e1007857. doi: 10.1371/journal.ppat.1007857 (PMC7004387; doi:10.1371/journal.ppat.1007857)
Supplement: S1 Text — (PDF) [file ppat.1007857.s001.pdf]

Text S1. Mathematical equations that parameterize the four investigated epidemiologic compartmental models.

Model 1

$$\frac{dS}{dt} = -\beta SI$$

$$\frac{dI}{dt} = \beta SI - \gamma I$$

Model 2

$$\frac{dS}{dt} = -\beta SI - \eta SU$$

$$\frac{dI}{dt} = \beta SI + \eta SU - \gamma I$$

Model 3

$$\frac{dS_T}{dt} = -\beta_T S_T T - \beta_{CT} S_T C$$

$$\frac{dS_C}{dt} = -\beta_C S_C C - \beta_{TC} S_C T$$

$$\frac{dT}{dt} = \beta_T S_T T + \beta_{CT} S_T C - \gamma_T T$$

$$\frac{dC}{dt} = \beta_C S_C C + \beta_{TC} S_C T - \gamma_C C$$

Model 4

$$\frac{dS_T}{dt} = -\beta_T S_T T - \beta_{CT} S_T C - \eta_T S_T U$$

$$\frac{dS_C}{dt} = -\beta_C S_C C - \beta_{TC} S_C T - \eta_C S_C U$$

$$\frac{dT}{dt} = \beta_T S_T T + \beta_{CT} S_T C + \eta_T S_T U - \gamma_T T$$

$$\frac{dC}{dt} = \beta_C S_C C + \beta_{TC} S_C T + \eta_C S_C U - \gamma_C$$

Parameter Definitions

S: homogenous susceptible farm population
I: homogenous infected farm population
β: transmission rate among poultry farms
γ: recovery rate (i.e., depopulation rate)
η: introduction rate from external source
U: external source population
S_T: susceptible turkey farm population
S_C: susceptible layer chicken farm population
T: infected turkey farm population
C: infected layer chicken farm population
β_T: transmission rate among turkey farms
β_C: transmission rate among layer chicken farms
β_{TC}: transmission rate of infection from turkey farms to layer chicken farms
β_{CT}: transmission rate of infection from layer chicken farms to turkey farms
γ_T: recovery rate (i.e., depopulation rate) of turkey farms
γ_C: recovery rate (i.e., depopulation rate) of layer chicken farms
η_T: introduction rate from external source into turkey farms
η_C: introduction rate from external source into layer chicken farms
