# Supplementary material for: Agricultural and geographic factors shaped the North American 2015 highly pathogenic avian influenza H5N2 outbreak
Source: PLoS Pathog. 2020 Jan 21;16(1):e1007857. doi: 10.1371/journal.ppat.1007857 (PMC7004387; doi:10.1371/journal.ppat.1007857)
Supplement: S1 Table — (PDF) [file ppat.1007857.s002.pdf]

Supplemental Table S1. Estimates of viral transmission between poultry industries during the 2015 highly pathogenic avian influenza virus H5N2 outbreak within the midwestern United States.

			Turkey → Layer Chicken		Layer Chicken → Turkey	
**Demographic Model**	**Parameter**	**Clock Model**	**Median**	**95% HPD***	**Median**	**95% HPD***
**Structured Coalescent**	Migration Rate	Strict	0.69	0.00001 – 2.2	12.56	6.2 – 18.7
		Relaxed	0.59	0.00001 – 2.1	12.84	6.4 – 18.8
**Discrete Trait Model**	Transition Rate	Strict	0.28	0.003 – 0.9	1.37	0.04 – 3.9
		Relaxed	0.28	0.004 – 0.9	1.38	0.04 – 3.9
**Compartmental Model 3**	β	Strict	0.10	0.02 – 0.2	4.91	0.7 – 9.6
		Relaxed	0.09	0.02 – 0.2	3.03	0.5 – 8.5

*95% Highest posterior density
