# Supplementary material for: Agricultural and geographic factors shaped the North American 2015 highly pathogenic avian influenza H5N2 outbreak
Source: PLoS Pathog. 2020 Jan 21;16(1):e1007857. doi: 10.1371/journal.ppat.1007857 (PMC7004387; doi:10.1371/journal.ppat.1007857)
Supplement: S2 Table — (PDF) [file ppat.1007857.s003.pdf]

Table S2. Akaike's information criteria for Markov chain Monte Carlo (AICM) for the epidemiological compartment-based coalescent models.

Molecular Clock	Coalescent Model	Structured Tree Likelihood AICM
Relaxed	Model 1	468.5112
Relaxed	Model 2	427.4448
Relaxed	Model 3	376.342
Relaxed	Model 4	401.958
Strict	Model 1	418.9891
Strict	Model 2	391.7703
Strict	Model 3	330.0827
Strict	Model 4	360.35
