# Supplementary material for: Agricultural and geographic factors shaped the North American 2015 highly pathogenic avian influenza H5N2 outbreak
Source: PLoS Pathog. 2020 Jan 21;16(1):e1007857. doi: 10.1371/journal.ppat.1007857 (PMC7004387; doi:10.1371/journal.ppat.1007857)
Supplement: S3 Table — Posterior probability, likelihood, structured tree (ST) likelihood, and prior probability are provided. Each model was performed under two different molecular clock assumptions (lognormal relaxed and strict). Median values with corresponding 95% highest posterior density (HPD) are displayed. Refer to Appendix B: Text S3.1 for parameter definitions. (PDF) [file ppat.1007857.s004.pdf]

Table S3. Parameter estimates of the Bayesian framework epidemiologic compartmental models. Posterior probability, likelihood, structured tree (ST) likelihood, and prior probability are provided. Each model was performed under two different molecular clock assumptions (lognormal relaxed and strict). Median values with corresponding 95% highest posterior density (HPD) are displayed. Refer to Appendix B: Text S3.1 for parameter definitions.

		Relaxed Molecular Clock			Strict Molecular Clock		
		median	95% HPD		median	95% HPD	
Model 1	Posterior	-25269.76	-25304.27	-25233.23	-25317.28	-25344.60	-25289.86
	Likelihood	-25154.38	-25183.26	-25126.72	-25196.55	-25219.74	-25174.49
	ST likelihood	-96.71	-119.50	-73.80	-102.58	-123.07	-82.81
	Prior	-115.01	-138.21	-91.83	-120.66	-141.29	-100.45
	β	0.66	0.09	2.51	0.57	0.11	2.05
	γ	22.51	16.44	29.93	22.16	16.26	29.80
	Initial S	92.67	11.55	261.45	98.99	10.51	284.38
	Initial I	6.09	2.71	10.28	7.50	4.01	12.02
Model 2	Posterior	-25271.81	-25305.67	-25237.50	-25310.55	-25337.55	-25284.12
	Likelihood	-25154.79	-25183.67	-25126.43	-25192.12	-25214.66	-25170.75
	ST likelihood	-92.08	-114.20	-70.99	-94.05	-113.33	-73.54
	Prior	-116.85	-138.65	-95.64	-118.28	-137.55	-98.79
	β	10.43	1.44	23.05	9.95	2.55	21.66
	η	1.22	0.06	7.35	1.30	0.07	6.40
	γ	39.53	22.96	57.66	41.17	24.81	60.39
	Initial S	8.69	3.97	16.26	9.02	4.30	15.18
	Initial I	0.45	0.00	1.62	0.46	0.00	1.67
	U size	29.04	2.07	132.74	28.70	1.94	165.55
Model 3	Posterior	-25253.80	-25290.07	-25219.58	-25288.03	-25316.55	-25260.85
	Likelihood	-25157.48	-25188.14	-25128.55	-25191.99	-25214.16	-25170.02
	ST likelihood	-56.28	-78.37	-33.62	-55.42	-75.46	-34.64
	Prior	-96.18	-118.38	-74.05	-95.95	-115.43	-75.21
	β_T	9.69	1.40	19.60	11.60	2.04	21.84
	β_C	1.55	0.37	3.59	1.73	0.48	3.79
	β_{TC}	0.09	0.02	0.21	0.10	0.02	0.22
	β_{CT}	4.03	0.55	8.46	4.91	0.65	9.60
	γ_T	58.24	32.82	80.04	64.24	34.58	83.66
	γ_C	11.65	7.46	16.64	11.39	7.44	16.21
	Initial S_T	30.06	12.09	56.11	28.31	11.68	50.41
	Initial S_C	31.81	11.03	72.85	29.03	10.14	60.95
	Initial T	1.83	0.05	7.59	1.94	0.09	7.94
	Initial C	0.12	0.00	0.65	0.12	0.00	0.63
Model 4	Posterior	-25260.67	-25297.46	-25226.45	-25295.52	-25325.08	-25268.14
	Likelihood	-25157.34	-25187.61	-25129.40	-25191.32	-25214.60	-25170.05
	ST likelihood	-58.61	-82.14	-35.36	-58.99	-80.94	-37.77
	Prior	-103.31	-126.47	-79.90	-104.32	-125.65	-83.58
	β_T	9.32	1.66	19.17	9.75	1.40	20.69
	β_C	1.87	0.35	8.45	2.26	0.47	8.38
	β_{TC}	0.12	0.02	0.49	0.14	0.02	0.50
	β_{CT}	4.20	0.72	8.74	4.43	0.59	9.38
	η_T	1.25	0.01	6.41	1.34	0.00	6.40
	η_C	0.11	0.00	0.72	0.09	0.00	0.68
	γ_T	60.81	36.10	81.56	63.51	36.83	84.40
	γ_C	11.47	7.44	16.33	11.16	7.41	15.80
	Initial S_T	26.80	10.37	48.13	25.62	10.45	46.27
	Initial S_C	24.30	2.96	56.95	20.73	4.59	48.07
	Initial T	0.99	0.05	2.90	1.04	0.05	3.10
	Initial C	0.22	0.01	0.76	0.18	0.01	0.68
	U size	2.69	0.03	20.80	4.07	0.02	32.09
