# Supplementary material for: Agricultural and geographic factors shaped the North American 2015 highly pathogenic avian influenza H5N2 outbreak
Source: PLoS Pathog. 2020 Jan 21;16(1):e1007857. doi: 10.1371/journal.ppat.1007857 (PMC7004387; doi:10.1371/journal.ppat.1007857)
Supplement: S4 Table — Median rates and associated 95% highest posterior density intervals (in brackets) are presented in each cell. The diffusion model is asymmetrical, and therefore, rates have directionality from a source county group (indicated on the left) to a sink county group (indicated across the top). County groups were defined by state (IA—Iowa, MN—Minnesota, ND—North Dakota, NE—Nebraska, SD—South Dakota, WI—Wisconsin) and composition of poultry type (T—turkey exclusive, CM—layer chicken exclusive and mixed poultry). Rates are colored by the level of Bayes factor support: no support (BF < 3.0), substantial support (3.0 ≤ BF < 10.0), strong support (10.0 ≤ BF < 30.0), very strong support (30.0 ≤ BF < 100.0), and decisive support (BF ≥ 100.0). (PDF) [file ppat.1007857.s005.pdf]

Supplemental Table S4. Discrete trait diffusion matrix of the midwestern highly pathogenic avian influenza (HPAI) H5N2 outbreak, 2015. Median rates and associated 95% highest posterior density intervals (in brackets) are presented in each cell. The diffusion model is asymmetrical, and therefore, rates have directionality from a source county group (indicated on the left) to a sink county group (indicated across the top). County groups were defined by state (IA - Iowa, MN - Minnesota, ND - North Dakota, NE - Nebraska, SD - South Dakota, WI - Wisconsin) and composition of poultry type (T - turkey exclusive, CM - layer chicken exclusive and mixed poultry). Rates are colored by the level of Bayes factor support. Gray rates represent no support.

	Sink									
Source	IA-CM	IA-T	MN-CM	MN-T	ND-T	NE-CM	SD-CM	SD-T	WI-CM	WI-T
IA-CM		3.3<br>[1.4, 5.7]	0.1<br>[0.0, 0.7]	0.9<br>[0.2, 2.2]	0.1<br>[0.0, 0.7]	0.5<br>[0.1, 1.4]	0.5<br>[0.0, 1.3]	0.5<br>[0.0, 1.4]	0.2<br>[0.0, 0.8]	0.2<br>[0.0, 0.9]
IA-T	0.7<br>[0.0, 2.1]		0.2<br>[0.0, 1.4]	0.3<br>[0.0, 1.3]	0.3<br>[0.0, 1.3]	0.6<br>[0.0, 2.0]	0.8<br>[0.0, 2.1]	0.5<br>[0.0, 1.9]	0.3<br>[0.0, 1.5]	0.3<br>[0.0, 1.2]
MN-CM	0.7<br>[0.0, 2.0]	0.5<br>[0.0, 1.5]		3.3<br>[0.7, 6.4]	0.6<br>[0.0, 1.8]	0.2<br>[0.0, 0.8]	0.2<br>[0.0, 1.1]	0.4<br>[0.0, 2.0]	0.4<br>[0.0, 1.5]	0.3<br>[0.0, 1.5]
MN-T	0.6<br>[0.0, 1.4]	0.3<br>[0.0, 1.1]	2.3<br>[0.6, 4.6]		0.5<br>[0.0, 1.3]	0.1<br>[0.0, 0.8]	0.1<br>[0.0, 0.5]	0.8<br>[0.1, 1.8]	0.3<br>[0.0, 1.0]	0.7<br>[0.1, 1.8]
ND-T	0.4<br>[0.0, 1.5]	0.5<br>[0.0, 1.7]	0.4<br>[0.0, 1.6]	0.3<br>[0.0, 1.6]		0.4<br>[0.0, 1.7]	0.3<br>[0.0, 1.4]	0.4<br>[0.0, 1.9]	0.5<br>[0.0, 1.9]	0.3<br>[0.0, 1.4]
NE-CM	0.3<br>[0.0, 1.5]	0.3<br>[0.0, 1.2]	0.3<br>[0.0, 1.4]	0.3<br>[0.0, 1.2]	0.3<br>[0.0, 1.3]		0.3<br>[0.0, 1.5]	0.4<br>[0.0, 1.5]	0.3<br>[0.0, 1.3]	0.4<br>[0.0, 1.6]
SD-CM	0.4<br>[0.0, 1.6]	0.4<br>[0.0, 1.5]	0.3<br>[0.0, 1.5]	0.3<br>[0.0, 1.8]	0.4<br>[0.0, 1.5]	0.4<br>[0.0, 1.7]		0.5<br>[0.0, 1.7]	0.4<br>[0.0, 1.5]	0.3<br>[0.0, 1.3]
SD-T	0.5<br>[0.0, 2.1]	0.3<br>[0.0, 1.4]	0.4<br>[0.0, 1.2]	0.4<br>[0.0, 1.4]	0.8<br>[0.0, 2.4]	0.4<br>[0.0, 2.1]	0.3<br>[0.0, 1.3]		0.6<br>[0.0, 2.1]	0.4<br>[0.0, 1.6]
WI-CM	0.3<br>[0.0, 1.4]	0.4<br>[0.0, 1.5]	0.4<br>[0.0, 1.4]	0.4<br>[0.0, 1.5]	0.4<br>[0.0, 1.7]	0.3<br>[0.0, 1.5]	0.3<br>[0.0, 1.3]	0.4<br>[0.0, 1.6]		0.3<br>[0.0, 1.2]
WI-T	0.9<br>[0.0, 2.6]	0.5<br>[0.0, 1.4]	0.3<br>[0.0, 1.4]	0.3<br>[0.0, 1.4]	0.5<br>[0.0, 2.1]	0.3<br>[0.0, 1.5]	0.2<br>[0.0, 1.0]	0.4<br>[0.0, 1.8]	0.4<br>[0.0, 1.6]	

Bayes Factor

Decisive Very Strong Strong Substantial No Support
