# Supplementary material for: Agricultural and geographic factors shaped the North American 2015 highly pathogenic avian influenza H5N2 outbreak
Source: PLoS Pathog. 2020 Jan 21;16(1):e1007857. doi: 10.1371/journal.ppat.1007857 (PMC7004387; doi:10.1371/journal.ppat.1007857)
Supplement: S5 Table — Two diffusion matrices were estimated: before and after April 10, 2015. Median rates and associated 95% highest posterior density intervals (in brackets) are presented in each cell. The diffusion model is asymmetrical, and therefore, rates have directionality from a source county group (indicated on the left) to a sink county group (indicated across the top). County groups were defined by state (IA—Iowa, MN—Minnesota, ND—North Dakota, NE—Nebraska, SD—South Dakota, WI—Wisconsin) and composition of poultry type (T—turkey exclusive, CM—layer chicken exclusive and mixed poultry). Rates are colored by the level of Bayes factor support: no support (BF < 3.0), substantial support (3.0 ≤ BF < 10.0), strong support (10.0 ≤ BF < 30.0), very strong support (30.0 ≤ BF < 100.0), and decisive support (BF ≥ 100.0). (PDF) [file ppat.1007857.s006.pdf]

| | | Sink | | | | | | | | | |
|---|---|---|---|---|---|---|---|---|---|---|---|
| Source | Epoch | IA-CM | IA-T | MN-CM | MN-T | ND-T | NE-CM | SD-CM | SD-T | WI-CM | WI-T |
| IA-CM | Before April 10, 2015 | | 0.5 [0.0, 2.4] | 0.6 [0.0, 2.7] | 0.5 [0.0, 2.1] | 0.4 [0.0, 2.2] | 0.4 [0.0, 2.3] | 0.5 [0.0, 2.4] | 0.8 [0.0, 3.4] | 0.6 [0.0, 2.7] | 1.0 [0.0, 3.7] |
| | After April 10, 2015 | | 2.0 [0.1, 5.1] | 0.2 [0.0, 1.1] | 0.3 [0.0, 0.9] | 0.4 [0.0, 1.9] | 0.3 [0.0, 2.4] | 0.8 [0.0, 3.4] | 1.0 [0.0, 3.0] | 0.2 [0.0, 1.6] | 0.3 [0.0, 1.4] |
| IA-T | Before April 10, 2015 | 0.5 [0.0, 2.7] | | 0.5 [0.0, 2.4] | 0.4 [0.0, 2.1] | 0.4 [0.0, 2.2] | 0.4 [0.0, 2.1] | 0.5 [0.0, 2.2] | 0.5 [0.0, 2.4] | 0.6 [0.0, 2.5] | 0.5 [0.0, 2.3] |
| | After April 10, 2015 | 1.0 [0.0, 3.2] | | 0.4 [0.0, 1.6] | 0.2 [0.0, 1.0] | 0.4 [0.0, 1.6] | 0.3 [0.0, 1.7] | 0.4 [0.0, 2.6] | 0.8 [0.0, 2.9] | 0.4 [0.0, 2.2] | 0.3 [0.0, 1.8] |
| MN-CM | Before April 10, 2015 | 0.7 [0.0, 3.0] | 0.3 [0.0, 1.7] | | 0.7 [0.0, 3.4] | 0.5 [0.0, 2.6] | 0.3 [0.0, 2.0] | 0.4 [0.0, 1.9] | 0.9 [0.0, 3.4] | 0.6 [0.0, 2.3] | 0.7 [0.0, 2.7] |
| | After April 10, 2015 | 0.6 [0.0, 2.4] | 0.4 [0.0, 1.6] | | 2.9 [0.6, 6.5] | 0.5 [0.0, 2.7] | 0.2 [0.0, 1.6] | 0.5 [0.0, 2.1] | 0.5 [0.0, 2.0] | 0.3 [0.0, 1.7] | 0.4 [0.0, 2.1] |
| MN-T | Before April 10, 2015 | 1.1 [0.0, 3.3] | 0.3 [0.0, 1.6] | 1.8 [0.2, 4.6] | | 0.6 [0.0, 2.6] | 0.3 [0.0, 1.4] | 0.3 [0.0, 1.9] | 1.3 [0.0, 3.7] | 0.6 [0.0, 2.5] | 0.8 [0.0, 2.7] |
| | After April 10, 2015 | 0.6 [0.0, 2.2] | 0.4 [0.0, 1.5] | 1.6 [0.0, 4.3] | | 0.4 [0.0, 2.1] | 0.3 [0.0, 1.4] | 0.4 [0.0, 1.6] | 0.4 [0.0, 1.9] | 0.3 [0.0, 1.4] | 0.5 [0.0, 2.2] |
| ND-T | Before April 10, 2015 | 0.5 [0.0, 2.6] | 0.3 [0.0, 1.8] | 0.5 [0.0, 2.9] | 0.3 [0.0, 1.9] | | 0.2 [0.0, 2.2] | 0.3 [0.0, 2.1] | 0.5 [0.0, 2.7] | 0.6 [0.0, 2.5] | 0.5 [0.0, 2.3] |
| | After April 10, 2015 | 0.4 [0.0, 2.3] | 0.3 [0.0, 1.7] | 0.4 [0.0, 2.2] | 0.3 [0.0, 1.6] | | 0.3 [0.0, 2.1] | 0.4 [0.0, 2.1] | 0.4 [0.0, 2.2] | 0.4 [0.0, 2.6] | 0.4 [0.0, 2.1] |
| NE-CM | Before April 10, 2015 | 0.4 [0.0, 2.2] | 0.3 [0.0, 1.7] | 0.4 [0.0, 2.1] | 0.3 [0.0, 1.8] | 0.3 [0.0, 1.8] | | 0.4 [0.0, 2.1] | 0.4 [0.0, 2.1] | 0.4 [0.0, 2.2] | 0.4 [0.0, 1.9] |
| | After April 10, 2015 | 0.4 [0.0, 1.9] | 0.2 [0.0, 1.5] | 0.3 [0.0, 1.7] | 0.2 [0.0, 1.1] | 0.3 [0.0, 1.9] | | 0.4 [0.0, 2.2] | 0.4 [0.0, 1.7] | 0.3 [0.0, 1.7] | 0.3 [0.0, 1.6] |
| SD-CM | Before April 10, 2015 | 0.5 [0.0, 2.6] | 0.4 [0.0, 2.3] | 0.5 [0.0, 2.6] | 0.3 [0.0, 2.5] | 0.5 [0.0, 2.4] | 0.5 [0.0, 2.1] | | 0.5 [0.0, 2.7] | 0.5 [0.0, 2.6] | 0.5 [0.0, 2.5] |
| | After April 10, 2015 | 0.5 [0.0, 2.4] | 0.4 [0.0, 2.7] | 0.4 [0.0, 2.1] | 0.3 [0.0, 1.9] | 0.4 [0.0, 1.8] | 0.6 [0.0, 2.3] | | 0.6 [0.0, 2.8] | 0.4 [0.0, 2.3] | 0.5 [0.0, 2.2] |
| SD-T | Before April 10, 2015 | 0.8 [0.0, 3.9] | 0.5 [0.0, 2.1] | 0.8 [0.0, 3.2] | 0.8 [0.0, 3.4] | 0.5 [0.0, 2.9] | 0.4 [0.0, 2.0] | 0.6 [0.0, 2.6] | | 0.8 [0.0, 3.1] | 0.8 [0.0, 3.1] |
| | After April 10, 2015 | 0.7 [0.0, 3.1] | 0.6 [0.0, 2.3] | 0.7 [0.0, 2.6] | 0.4 [0.0, 2.1] | 0.4 [0.0, 2.2] | 0.7 [0.0, 2.3] | 0.6 [0.0, 2.5] | | 0.6 [0.0, 2.3] | 0.6 [0.0, 2.5] |
| WI-CM | Before April 10, 2015 | 0.6 [0.0, 2.6] | 0.4 [0.0, 2.0] | 0.5 [0.0, 2.3] | 0.4 [0.0, 2.1] | 0.4 [0.0, 2.3] | 0.4 [0.0, 2.2] | 0.4 [0.0, 2.2] | 0.6 [0.0, 2.7] | | 0.6 [0.0, 2.9] |
| | After April 10, 2015 | 0.6 [0.0, 2.6] | 0.4 [0.0, 2.2] | 0.5 [0.0, 2.4] | 0.4 [0.0, 1.7] | 0.4 [0.0, 1.9] | 0.5 [0.0, 2.2] | 0.4 [0.0, 2.3] | 0.5 [0.0, 2.5] | | 0.4 [0.0, 2.0] |
| WI-T | Before April 10, 2015 | 1.3 [0.0, 4.0] | 0.4 [0.0, 2.4] | 0.6 [0.0, 2.8] | 0.4 [0.0, 1.8] | 0.4 [0.0, 2.1] | 0.3 [0.0, 2.0] | 0.4 [0.0, 2.2] | 0.6 [0.0, 2.9] | 0.5 [0.0, 2.4] | |
| | After April 10, 2015 | 0.8 [0.0, 3.3] | 0.5 [0.0, 2.2] | 0.5 [0.0, 2.4] | 0.3 [0.0, 1.6] | 0.4 [0.0, 2.2] | 0.4 [0.0, 2.0] | 0.5 [0.0, 2.1] | 0.5 [0.0, 2.3] | 0.4 [0.0, 2.4] | |

Decisive · Very Strong · Strong · Substantial · No Support
