# Supplementary material for: Agricultural and geographic factors shaped the North American 2015 highly pathogenic avian influenza H5N2 outbreak
Source: PLoS Pathog. 2020 Jan 21;16(1):e1007857. doi: 10.1371/journal.ppat.1007857 (PMC7004387; doi:10.1371/journal.ppat.1007857)
Supplement: S6 Table — (PDF) [file ppat.1007857.s007.pdf]

Table S6. Demographic and geographic characteristics of the 49 United States counties with HPAI-positive commercial poultry premises during the H5N2 outbreak, 2015.

	**Mean**	**Standard Deviation**	**Minimum**	**Maximum**
**Distance between counties** (km)	265.95	153.45	30.18	861.99
**Layer Chicken Farm Density** (farms/km^2)	0.02	0.02	0.001	0.09
**Turkey Farm Density** (farms/km^2)	0.004	0.004	3.97×10^{-6}	0.01
**Human Population Density** (humans/km^2)	12.53	11.21	1.39	58.07
**Road Density** (km/km^2)	1.89	0.32	1.18	2.78
**Water Coverage** (%)	1.56	2.21	0.02	11.34
**Important Bird Area** (%)	4.23	7.60	0.0	30.96
**Agricultural Land Use** (%)	78.07	13.28	32.46	90.62
**Frozen days**	19.82	4.93	12	38
