# Supplementary material for: Agricultural and geographic factors shaped the North American 2015 highly pathogenic avian influenza H5N2 outbreak
Source: PLoS Pathog. 2020 Jan 21;16(1):e1007857. doi: 10.1371/journal.ppat.1007857 (PMC7004387; doi:10.1371/journal.ppat.1007857)
Supplement: S8 Table — Conditional effect size and 95% highest posterior density (HPD) were calculated based on the estimated GLM coefficients given the Bayesian stochastic search variable selection (BSSVS) indicator = 1. The posterior probability (PP) refers to the proportion of Markov chain Monte Carlo (MCMC) samples in which the BSSVS indicator = 1. Bayes factor (BF) > 3.0 indicates statistical support for the inclusion of the covariate within the GLM. (PDF) [file ppat.1007857.s009.pdf]

Supplemental Table S7. Generalized linear model (GLM) conditional effect sizes and statistical support for agricultural and geographic covariates of the dispersal of highly pathogenic avian influenza (HPAI) H5N2 among midwestern county groups. Conditional effect size and 95% highest posterior density (HPD) were calculated based on the estimated GLM coefficients given the Bayesian stochastic search variable selection (BSSVS) indicator = 1. The posterior probability (PP) refers to the proportion of Markov chain Monte Carlo (MCMC) samples in which the BSSVS indicator = 1. Bayes factor (BF) > 3.0 indicates statistical support for the inclusion of the covariate within the GLM.

	Conditional Effect Size	95% HPD	PP	BF
Geographic distance	-1.02	(-1.25, -0.79)	1.00	216262.92
Layer Farm Density – Origin	-1.11	(-3.17, 1.97)	0.01	0.32
Layer Farm Density – Destination	-0.21	(-0.66, 0.18)	0.002	0.04
Turkey Farm Density – Origin	0.55	(-0.64, 1.32)	0.01	0.30
Turkey Farm Density – Destination	0.36	(-0.21, 0.90)	0.004	0.10
Human Population Density – Origin	1.21	(0.16, 2.87)	0.09	2.24
Human Population Density – Destination	0.22	(-0.28, 0.81)	0.001	0.03
IBA Proportion – Origin	0.22	(-0.76, 0.81)	0.004	0.10
IBA Proportion – Destination	0.53	(0.18, 0.87)	0.06	1.41
Road Density – Origin	1.23	(0.61, 1.72)	0.64	42.79
Road Density – Destination	0.25	(-0.19, 0.62)	0.002	0.05
Water Coverage – Origin	0.47	(-0.27, 0.97)	0.01	0.27
Proportion Surface Water – Destination	0.57	(0.18, 0.93)	0.14	3.85
Frozen Days – Origin	-0.32	(-0.99, 0.59)	0.001	0.03
Frozen Days – Destination	-0.21	(-0.35, -0.14)	0.001	0.01
Agriculture Land Use – Origin	0.41	(-0.54, 1.02)	0.002	0.04
Agriculture Land Use – Destination	-0.08	(-0.32, 0.32)	0.001	0.03
