# Supplementary material for: Agricultural and geographic factors shaped the North American 2015 highly pathogenic avian influenza H5N2 outbreak
Source: PLoS Pathog. 2020 Jan 21;16(1):e1007857. doi: 10.1371/journal.ppat.1007857 (PMC7004387; doi:10.1371/journal.ppat.1007857)
Supplement: S9 Table — Conditional effect size represents the effect size of the variable coefficient given inclusion in the GLM. Supported covariates (Bayes factor > 3) are bolded. Covariates are ordered by Bayes factor. (PDF) [file ppat.1007857.s010.pdf]

Supplemental Table S8. Median conditional effect sizes of environmental and geographic covariates within the generalized linear model (GLM), with highly correlated variables removed. Conditional effect size represents the effect size of the variable coefficient given inclusion in the GLM. Supported covariates (Bayes factor > 3) are bolded. Covariates are ordered by Bayes factor.

	Median	95% HPD[1]		pp[2]	BF[3]
**Geographic distance**	-1.03	-1.24	-0.79	1.00	164335.31
**Road density - Origin**	1.24	0.58	1.71	0.76	58.76
**Water coverage - Destination**	0.56	0.21	0.95	0.16	3.58
Water coverage - Origin	0.44	-0.36	0.80	0.02	0.31
Turkey farm density - Origin	0.50	-1.17	1.27	0.01	0.27
Layer farm density - Origin	-0.60	-2.09	1.58	0.01	0.24
Turkey farm density - Destination	0.34	0.03	0.81	0.01	0.11
Road density - Destination	0.27	-0.12	0.60	0.00	0.06
Agriculture land use - Origin	0.10	-0.50	0.74	0.00	0.06
Frozen days - Origin	0.04	-0.65	0.91	0.00	0.05
Agriculture land use - Destination	-0.14	-0.47	0.33	0.00	0.05
Layer farm density - Destination	-0.11	-0.60	0.34	0.00	0.04
Frozen days - Destination	-0.06	-0.24	0.46	0.00	0.03

[1]95% HPD: 95% highest posterior density
[2]pp: posterior probability of covariate inclusion
[3]BF: Bayes factor
