# Supplementary material for: Agricultural and geographic factors shaped the North American 2015 highly pathogenic avian influenza H5N2 outbreak
Source: PLoS Pathog. 2020 Jan 21;16(1):e1007857. doi: 10.1371/journal.ppat.1007857 (PMC7004387; doi:10.1371/journal.ppat.1007857)
Supplement: S10 Table — (PDF) [file ppat.1007857.s011.pdf]

Sequence Accession	Subtype	Collection Date	Strain Name	Segment	Gene
KR233979	H5N2	03/26/2015	A/turkey/Minnesota/9892-2/2015	1	PB2
KR233980	H5N2	03/26/2015	A/turkey/Minnesota/9892-2/2015	2	PB1
KR233981	H5N2	03/26/2015	A/turkey/Minnesota/9892-2/2015	3	PA
KR233982	H5N2	03/26/2015	A/turkey/Minnesota/9892-2/2015	4	HA
KR233983	H5N2	03/26/2015	A/turkey/Minnesota/9892-2/2015	5	NP
KR233984	H5N2	03/26/2015	A/turkey/Minnesota/9892-2/2015	6	NA
KR233985	H5N2	03/26/2015	A/turkey/Minnesota/9892-2/2015	7	M
KR233986	H5N2	03/26/2015	A/turkey/Minnesota/9892-2/2015	8	NS
KR234035	H5N2	03/25/2015	A/turkey/Minnesota/9845-4/2015	1	PB2
KR234036	H5N2	03/25/2015	A/turkey/Minnesota/9845-4/2015	2	PB1
KR234037	H5N2	03/25/2015	A/turkey/Minnesota/9845-4/2015	3	PA
KR234038	H5N2	03/25/2015	A/turkey/Minnesota/9845-4/2015	4	HA
KR234039	H5N2	03/25/2015	A/turkey/Minnesota/9845-4/2015	5	NP
KR234040	H5N2	03/25/2015	A/turkey/Minnesota/9845-4/2015	6	NA
KR234041	H5N2	03/25/2015	A/turkey/Minnesota/9845-4/2015	7	M
KR234042	H5N2	03/25/2015	A/turkey/Minnesota/9845-4/2015	8	NS
KT002470	H5N2	04/12/2015	A/turkey/Iowa/11762-1/2015	1	PB2
KT002471	H5N2	04/12/2015	A/turkey/Iowa/11762-1/2015	2	PB1
KT002472	H5N2	04/12/2015	A/turkey/Iowa/11762-1/2015	3	PA
KT002473	H5N2	04/12/2015	A/turkey/Iowa/11762-1/2015	4	HA
KT002474	H5N2	04/12/2015	A/turkey/Iowa/11762-1/2015	5	NP
KT002475	H5N2	04/12/2015	A/turkey/Iowa/11762-1/2015	6	NA
KT002476	H5N2	04/12/2015	A/turkey/Iowa/11762-1/2015	7	M
KT002477	H5N2	04/12/2015	A/turkey/Iowa/11762-1/2015	8	NS
KT002478	H5N2	04/25/2015	A/turkey/Iowa/13541-1/2015	1	PB2
KT002479	H5N2	04/25/2015	A/turkey/Iowa/13541-1/2015	2	PB1
KT002480	H5N2	04/25/2015	A/turkey/Iowa/13541-1/2015	3	PA
KT002481	H5N2	04/25/2015	A/turkey/Iowa/13541-1/2015	4	HA
KT002482	H5N2	04/25/2015	A/turkey/Iowa/13541-1/2015	5	NP
KT002483	H5N2	04/25/2015	A/turkey/Iowa/13541-1/2015	6	NA
KT002484	H5N2	04/25/2015	A/turkey/Iowa/13541-1/2015	7	M
KT002485	H5N2	04/25/2015	A/turkey/Iowa/13541-1/2015	8	NS
KT002486	H5N2	04/25/2015	A/chicken/Iowa/13542-2/2015	1	PB2
KT002487	H5N2	04/25/2015	A/chicken/Iowa/13542-2/2015	2	PB1
KT002488	H5N2	04/25/2015	A/chicken/Iowa/13542-2/2015	3	PA
KT002489	H5N2	04/25/2015	A/chicken/Iowa/13542-2/2015	4	HA
KT002490	H5N2	04/25/2015	A/chicken/Iowa/13542-2/2015	5	NP
KT002491	H5N2	04/25/2015	A/chicken/Iowa/13542-2/2015	6	NA
KT002492	H5N2	04/25/2015	A/chicken/Iowa/13542-2/2015	7	M
KT002493	H5N2	04/25/2015	A/chicken/Iowa/13542-2/2015	8	NS
KT002494	H5N2	04/30/2015	A/turkey/Iowa/14318-1/2015	1	PB2
KT002495	H5N2	04/30/2015	A/turkey/Iowa/14318-1/2015	2	PB1
KT002496	H5N2	04/30/2015	A/turkey/Iowa/14318-1/2015	3	PA
KT002497	H5N2	04/30/2015	A/turkey/Iowa/14318-1/2015	4	HA
KT002498	H5N2	04/30/2015	A/turkey/Iowa/14318-1/2015	5	NP
KT002499	H5N2	04/30/2015	A/turkey/Iowa/14318-1/2015	6	NA
KT002500	H5N2	04/30/2015	A/turkey/Iowa/14318-1/2015	7	M
KT002501	H5N2	04/30/2015	A/turkey/Iowa/14318-1/2015	8	NS
KT002502	H5N2	05/01/2015	A/turkey/Iowa/14319-1/2015	1	PB2
KT002503	H5N2	05/01/2015	A/turkey/Iowa/14319-1/2015	2	PB1
KT002504	H5N2	05/01/2015	A/turkey/Iowa/14319-1/2015	3	PA
KT002505	H5N2	05/01/2015	A/turkey/Iowa/14319-1/2015	4	HA
KT002506	H5N2	05/01/2015	A/turkey/Iowa/14319-1/2015	5	NP
KT002507	H5N2	05/01/2015	A/turkey/Iowa/14319-1/2015	6	NA
KT002508	H5N2	05/01/2015	A/turkey/Iowa/14319-1/2015	7	M
KT002509	H5N2	05/01/2015	A/turkey/Iowa/14319-1/2015	8	NS
KT002518	H5N2	04/30/2015	A/chicken/Iowa/14399-4/2015	1	PB2
KT002519	H5N2	04/30/2015	A/chicken/Iowa/14399-4/2015	2	PB1
KT002520	H5N2	04/30/2015	A/chicken/Iowa/14399-4/2015	3	PA
KT002521	H5N2	04/30/2015	A/chicken/Iowa/14399-4/2015	4	HA
KT002522	H5N2	04/30/2015	A/chicken/Iowa/14399-4/2015	5	NP
KT002523	H5N2	04/30/2015	A/chicken/Iowa/14399-4/2015	6	NA
KT002524	H5N2	04/30/2015	A/chicken/Iowa/14399-4/2015	7	M
KT002525	H5N2	04/30/2015	A/chicken/Iowa/14399-4/2015	8	NS
KT762889	H5N2	04/10/2015	A/turkey/North Dakota/15-011420-13/2015	1	PB2
KT762890	H5N2	04/10/2015	A/turkey/North Dakota/15-011420-13/2015	2	PB1
KT762891	H5N2	04/10/2015	A/turkey/North Dakota/15-011420-13/2015	3	PA
KT762892	H5N2	04/10/2015	A/turkey/North Dakota/15-011420-13/2015	4	HA
KT762893	H5N2	04/10/2015	A/turkey/North Dakota/15-011420-13/2015	5	NP
KT762894	H5N2	04/10/2015	A/turkey/North Dakota/15-011420-13/2015	6	NA
KT762895	H5N2	04/10/2015	A/turkey/North Dakota/15-011420-13/2015	7	M
KT762896	H5N2	04/10/2015	A/turkey/North Dakota/15-011420-13/2015	8	NS
KT762897	H5N2	06/01/2015	A/chicken/Nebraska/15-017897-1/2015	1	PB2
KT762898	H5N2	06/01/2015	A/chicken/Nebraska/15-017897-1/2015	2	PB1
KT762899	H5N2	06/01/2015	A/chicken/Nebraska/15-017897-1/2015	3	PA
KT762900	H5N2	06/01/2015	A/chicken/Nebraska/15-017897-1/2015	4	HA
KT762901	H5N2	06/01/2015	A/chicken/Nebraska/15-017897-1/2015	5	NP
KT762902	H5N2	06/01/2015	A/chicken/Nebraska/15-017897-1/2015	6	NA
KT762903	H5N2	06/01/2015	A/chicken/Nebraska/15-017897-1/2015	7	M
KT762904	H5N2	06/01/2015	A/chicken/Nebraska/15-017897-1/2015	8	NS
KT762905	H5N2	04/14/2015	A/turkey/Wisconsin/15-012012-2/2015	1	PB2
KT762906	H5N2	04/14/2015	A/turkey/Wisconsin/15-012012-2/2015	2	PB1
KT762907	H5N2	04/14/2015	A/turkey/Wisconsin/15-012012-2/2015	3	PA
KT762908	H5N2	04/14/2015	A/turkey/Wisconsin/15-012012-2/2015	4	HA
KT762909	H5N2	04/14/2015	A/turkey/Wisconsin/15-012012-2/2015	5	NP
KT762910	H5N2	04/14/2015	A/turkey/Wisconsin/15-012012-2/2015	6	NA
KT762911	H5N2	04/14/2015	A/turkey/Wisconsin/15-012012-2/2015	7	M
KT762912	H5N2	04/14/2015	A/turkey/Wisconsin/15-012012-2/2015	8	NS
KT762913	H5N2	04/21/2015	A/turkey/North Dakota/15-013049-1/2015	1	PB2

KT762914	H5N2	04/21/2015	A/turkey/North Dakota/15-013049-1/2015	2	PB1
KT762915	H5N2	04/21/2015	A/turkey/North Dakota/15-013049-1/2015	3	PA
KT762916	H5N2	04/21/2015	A/turkey/North Dakota/15-013049-1/2015	4	HA
KT762917	H5N2	04/21/2015	A/turkey/North Dakota/15-013049-1/2015	5	NP
KT762918	H5N2	04/21/2015	A/turkey/North Dakota/15-013049-1/2015	6	NA
KT762919	H5N2	04/21/2015	A/turkey/North Dakota/15-013049-1/2015	7	M
KT762920	H5N2	04/21/2015	A/turkey/North Dakota/15-013049-1/2015	8	NS
KT762921	H5N2	04/26/2015	A/chicken/Minnesota/15-013533-1/2015	1	PB2
KT762922	H5N2	04/26/2015	A/chicken/Minnesota/15-013533-1/2015	2	PB1
KT762923	H5N2	04/26/2015	A/chicken/Minnesota/15-013533-1/2015	3	PA
KT762924	H5N2	04/26/2015	A/chicken/Minnesota/15-013533-1/2015	4	HA
KT762925	H5N2	04/26/2015	A/chicken/Minnesota/15-013533-1/2015	5	NP
KT762926	H5N2	04/26/2015	A/chicken/Minnesota/15-013533-1/2015	6	NA
KT762927	H5N2	04/26/2015	A/chicken/Minnesota/15-013533-1/2015	7	M
KT762928	H5N2	04/26/2015	A/chicken/Minnesota/15-013533-1/2015	8	NS
KT762929	H5N2	03/30/2015	A/turkey/South Dakota/15-010371/2015	1	PB2
KT762930	H5N2	03/30/2015	A/turkey/South Dakota/15-010371/2015	2	PB1
KT762931	H5N2	03/30/2015	A/turkey/South Dakota/15-010371/2015	3	PA
KT762932	H5N2	03/30/2015	A/turkey/South Dakota/15-010371/2015	4	HA
KT762933	H5N2	03/30/2015	A/turkey/South Dakota/15-010371/2015	5	NP
KT762934	H5N2	03/30/2015	A/turkey/South Dakota/15-010371/2015	6	NA
KT762935	H5N2	03/30/2015	A/turkey/South Dakota/15-010371/2015	7	M
KT762936	H5N2	03/30/2015	A/turkey/South Dakota/15-010371/2015	8	NS
KT762945	H5N2	04/08/2015	A/chicken/Wisconsin/15-011595-1/2015	1	PB2
KT762946	H5N2	04/08/2015	A/chicken/Wisconsin/15-011595-1/2015	2	PB1
KT762947	H5N2	04/08/2015	A/chicken/Wisconsin/15-011595-1/2015	3	PA
KT762948	H5N2	04/08/2015	A/chicken/Wisconsin/15-011595-1/2015	4	HA
KT762949	H5N2	04/08/2015	A/chicken/Wisconsin/15-011595-1/2015	5	NP
KT762950	H5N2	04/08/2015	A/chicken/Wisconsin/15-011595-1/2015	6	NA
KT762951	H5N2	04/08/2015	A/chicken/Wisconsin/15-011595-1/2015	7	M
KT762952	H5N2	04/08/2015	A/chicken/Wisconsin/15-011595-1/2015	8	NS
KX351768	H5N2	04/16/2015	A/turkey/South Dakota/15-012511-2/2015	1	PB2
KX351769	H5N2	04/16/2015	A/turkey/South Dakota/15-012511-2/2015	2	PB1
KX351770	H5N2	04/16/2015	A/turkey/South Dakota/15-012511-2/2015	3	PA
KX351771	H5N2	04/16/2015	A/turkey/South Dakota/15-012511-2/2015	4	HA
KX351772	H5N2	04/16/2015	A/turkey/South Dakota/15-012511-2/2015	5	NP
KX351773	H5N2	04/16/2015	A/turkey/South Dakota/15-012511-2/2015	6	NA
KX351774	H5N2	04/16/2015	A/turkey/South Dakota/15-012511-2/2015	7	M
KX351775	H5N2	04/16/2015	A/turkey/South Dakota/15-012511-2/2015	8	NS
KX351776	H5N2	04/18/2015	A/turkey/Minnesota/15-012582-1/2015	1	PB2
KX351777	H5N2	04/18/2015	A/turkey/Minnesota/15-012582-1/2015	2	PB1
KX351778	H5N2	04/18/2015	A/turkey/Minnesota/15-012582-1/2015	3	PA
KX351779	H5N2	04/18/2015	A/turkey/Minnesota/15-012582-1/2015	4	HA
KX351780	H5N2	04/18/2015	A/turkey/Minnesota/15-012582-1/2015	5	NP
KX351781	H5N2	04/18/2015	A/turkey/Minnesota/15-012582-1/2015	6	NA
KX351782	H5N2	04/18/2015	A/turkey/Minnesota/15-012582-1/2015	7	M
KX351783	H5N2	04/18/2015	A/turkey/Minnesota/15-012582-1/2015	8	NS
KX351784	H5N2	04/26/2015	A/chicken/Iowa/15-013388-1/2015	1	PB2
KX351785	H5N2	04/26/2015	A/chicken/Iowa/15-013388-1/2015	2	PB1
KX351786	H5N2	04/26/2015	A/chicken/Iowa/15-013388-1/2015	3	PA
KX351787	H5N2	04/26/2015	A/chicken/Iowa/15-013388-1/2015	4	HA
KX351788	H5N2	04/26/2015	A/chicken/Iowa/15-013388-1/2015	5	NP
KX351789	H5N2	04/26/2015	A/chicken/Iowa/15-013388-1/2015	6	NA
KX351790	H5N2	04/26/2015	A/chicken/Iowa/15-013388-1/2015	7	M
KX351791	H5N2	04/26/2015	A/chicken/Iowa/15-013388-1/2015	8	NS
MG964373	H5N2	04/19/2015	A/chicken/Iowa/15-012564-7/2015	1	PB2
MG964374	H5N2	04/19/2015	A/chicken/Iowa/15-012564-7/2015	2	PB1
MG964375	H5N2	04/19/2015	A/chicken/Iowa/15-012564-7/2015	3	PA
MG964376	H5N2	04/19/2015	A/chicken/Iowa/15-012564-7/2015	4	HA
MG964377	H5N2	04/19/2015	A/chicken/Iowa/15-012564-7/2015	5	NP
MG964378	H5N2	04/19/2015	A/chicken/Iowa/15-012564-7/2015	6	NA
MG964379	H5N2	04/19/2015	A/chicken/Iowa/15-012564-7/2015	7	M
MG964380	H5N2	04/19/2015	A/chicken/Iowa/15-012564-7/2015	8	NS
MG964381	H5N2	2015	A/chicken/Iowa/15-013408-3/2015	1	PB2
MG964382	H5N2	2015	A/chicken/Iowa/15-013408-3/2015	2	PB1
MG964383	H5N2	2015	A/chicken/Iowa/15-013408-3/2015	3	PA
MG964384	H5N2	2015	A/chicken/Iowa/15-013408-3/2015	4	HA
MG964385	H5N2	2015	A/chicken/Iowa/15-013408-3/2015	5	NP
MG964386	H5N2	2015	A/chicken/Iowa/15-013408-3/2015	6	NA
MG964387	H5N2	2015	A/chicken/Iowa/15-013408-3/2015	7	M
MG964388	H5N2	2015	A/chicken/Iowa/15-013408-3/2015	8	NS
MG964389	H5N2	04/26/2015	A/chicken/Iowa/15-013430-16/2015	1	PB2
MG964390	H5N2	04/26/2015	A/chicken/Iowa/15-013430-16/2015	2	PB1
MG964391	H5N2	04/26/2015	A/chicken/Iowa/15-013430-16/2015	3	PA
MG964392	H5N2	04/26/2015	A/chicken/Iowa/15-013430-16/2015	4	HA
MG964393	H5N2	04/26/2015	A/chicken/Iowa/15-013430-16/2015	5	NP
MG964394	H5N2	04/26/2015	A/chicken/Iowa/15-013430-16/2015	6	NA
MG964395	H5N2	04/26/2015	A/chicken/Iowa/15-013430-16/2015	7	M
MG964396	H5N2	04/26/2015	A/chicken/Iowa/15-013430-16/2015	8	NS
MG964397	H5N2	04/27/2015	A/chicken/Iowa/15-013540-6/2015	1	PB2
MG964398	H5N2	04/27/2015	A/chicken/Iowa/15-013540-6/2015	2	PB1
MG964399	H5N2	04/27/2015	A/chicken/Iowa/15-013540-6/2015	3	PA
MG964400	H5N2	04/27/2015	A/chicken/Iowa/15-013540-6/2015	4	HA
MG964401	H5N2	04/27/2015	A/chicken/Iowa/15-013540-6/2015	5	NP
MG964402	H5N2	04/27/2015	A/chicken/Iowa/15-013540-6/2015	6	NA
MG964403	H5N2	04/27/2015	A/chicken/Iowa/15-013540-6/2015	7	M
MG964404	H5N2	04/27/2015	A/chicken/Iowa/15-013540-6/2015	8	NS
MG964405	H5N2	2015	A/chicken/Iowa/15-013784-2/2015	1	PB2
MG964406	H5N2	2015	A/chicken/Iowa/15-013784-2/2015	2	PB1
MG964407	H5N2	2015	A/chicken/Iowa/15-013784-2/2015	3	PA

MG964408	H5N2	2015	A/chicken/Iowa/15-013784-2/2015	4	HA
MG964409	H5N2	2015	A/chicken/Iowa/15-013784-2/2015	5	NP
MG964410	H5N2	2015	A/chicken/Iowa/15-013784-2/2015	6	NA
MG964411	H5N2	2015	A/chicken/Iowa/15-013784-2/2015	7	M
MG964412	H5N2	2015	A/chicken/Iowa/15-013784-2/2015	8	NS
MG964413	H5N2	04/29/2015	A/chicken/Iowa/15-013972-1/2015	1	PB2
MG964414	H5N2	04/29/2015	A/chicken/Iowa/15-013972-1/2015	2	PB1
MG964415	H5N2	04/29/2015	A/chicken/Iowa/15-013972-1/2015	3	PA
MG964416	H5N2	04/29/2015	A/chicken/Iowa/15-013972-1/2015	4	HA
MG964417	H5N2	04/29/2015	A/chicken/Iowa/15-013972-1/2015	5	NP
MG964418	H5N2	04/29/2015	A/chicken/Iowa/15-013972-1/2015	6	NA
MG964419	H5N2	04/29/2015	A/chicken/Iowa/15-013972-1/2015	7	M
MG964420	H5N2	04/29/2015	A/chicken/Iowa/15-013972-1/2015	8	NS
MG964421	H5N2	2015	A/chicken/Iowa/15-013976-6/2015	1	PB2
MG964422	H5N2	2015	A/chicken/Iowa/15-013976-6/2015	2	PB1
MG964423	H5N2	2015	A/chicken/Iowa/15-013976-6/2015	3	PA
MG964424	H5N2	2015	A/chicken/Iowa/15-013976-6/2015	4	HA
MG964425	H5N2	2015	A/chicken/Iowa/15-013976-6/2015	5	NP
MG964426	H5N2	2015	A/chicken/Iowa/15-013976-6/2015	6	NA
MG964427	H5N2	2015	A/chicken/Iowa/15-013976-6/2015	7	M
MG964428	H5N2	2015	A/chicken/Iowa/15-013976-6/2015	8	NS
MG964429	H5N2	04/29/2015	A/chicken/Iowa/15-013982-1/2015	1	PB2
MG964430	H5N2	04/29/2015	A/chicken/Iowa/15-013982-1/2015	2	PB1
MG964431	H5N2	04/29/2015	A/chicken/Iowa/15-013982-1/2015	3	PA
MG964432	H5N2	04/29/2015	A/chicken/Iowa/15-013982-1/2015	4	HA
MG964433	H5N2	04/29/2015	A/chicken/Iowa/15-013982-1/2015	5	NP
MG964434	H5N2	04/29/2015	A/chicken/Iowa/15-013982-1/2015	6	NA
MG964435	H5N2	04/29/2015	A/chicken/Iowa/15-013982-1/2015	7	M
MG964436	H5N2	04/29/2015	A/chicken/Iowa/15-013982-1/2015	8	NS
MG964437	H5N2	05/02/2015	A/chicken/Iowa/15-014283-1/2015	1	PB2
MG964438	H5N2	05/02/2015	A/chicken/Iowa/15-014283-1/2015	2	PB1
MG964439	H5N2	05/02/2015	A/chicken/Iowa/15-014283-1/2015	3	PA
MG964440	H5N2	05/02/2015	A/chicken/Iowa/15-014283-1/2015	4	HA
MG964441	H5N2	05/02/2015	A/chicken/Iowa/15-014283-1/2015	5	NP
MG964442	H5N2	05/02/2015	A/chicken/Iowa/15-014283-1/2015	6	NA
MG964443	H5N2	05/02/2015	A/chicken/Iowa/15-014283-1/2015	7	M
MG964444	H5N2	05/02/2015	A/chicken/Iowa/15-014283-1/2015	8	NS
MG964445	H5N2	05/03/2015	A/chicken/Iowa/15-014294-1/2015	1	PB2
MG964446	H5N2	05/03/2015	A/chicken/Iowa/15-014294-1/2015	2	PB1
MG964447	H5N2	05/03/2015	A/chicken/Iowa/15-014294-1/2015	3	PA
MG964448	H5N2	05/03/2015	A/chicken/Iowa/15-014294-1/2015	4	HA
MG964449	H5N2	05/03/2015	A/chicken/Iowa/15-014294-1/2015	5	NP
MG964450	H5N2	05/03/2015	A/chicken/Iowa/15-014294-1/2015	6	NA
MG964451	H5N2	05/03/2015	A/chicken/Iowa/15-014294-1/2015	7	M
MG964452	H5N2	05/03/2015	A/chicken/Iowa/15-014294-1/2015	8	NS
MG964453	H5N2	05/04/2015	A/chicken/Iowa/15-014572-1/2015	1	PB2
MG964454	H5N2	05/04/2015	A/chicken/Iowa/15-014572-1/2015	2	PB1
MG964455	H5N2	05/04/2015	A/chicken/Iowa/15-014572-1/2015	3	PA
MG964456	H5N2	05/04/2015	A/chicken/Iowa/15-014572-1/2015	4	HA
MG964457	H5N2	05/04/2015	A/chicken/Iowa/15-014572-1/2015	5	NP
MG964458	H5N2	05/04/2015	A/chicken/Iowa/15-014572-1/2015	6	NA
MG964459	H5N2	05/04/2015	A/chicken/Iowa/15-014572-1/2015	7	M
MG964460	H5N2	05/04/2015	A/chicken/Iowa/15-014572-1/2015	8	NS
MG964461	H5N2	2015	A/chicken/Iowa/15-014586-1/2015	1	PB2
MG964462	H5N2	2015	A/chicken/Iowa/15-014586-1/2015	2	PB1
MG964463	H5N2	2015	A/chicken/Iowa/15-014586-1/2015	3	PA
MG964464	H5N2	2015	A/chicken/Iowa/15-014586-1/2015	4	HA
MG964465	H5N2	2015	A/chicken/Iowa/15-014586-1/2015	5	NP
MG964466	H5N2	2015	A/chicken/Iowa/15-014586-1/2015	6	NA
MG964467	H5N2	2015	A/chicken/Iowa/15-014586-1/2015	7	M
MG964468	H5N2	2015	A/chicken/Iowa/15-014586-1/2015	8	NS
MG964469	H5N2	05/02/2015	A/chicken/Iowa/15-014591-1/2015	1	PB2
MG964470	H5N2	05/02/2015	A/chicken/Iowa/15-014591-1/2015	2	PB1
MG964471	H5N2	05/02/2015	A/chicken/Iowa/15-014591-1/2015	3	PA
MG964472	H5N2	05/02/2015	A/chicken/Iowa/15-014591-1/2015	4	HA
MG964473	H5N2	05/02/2015	A/chicken/Iowa/15-014591-1/2015	5	NP
MG964474	H5N2	05/02/2015	A/chicken/Iowa/15-014591-1/2015	6	NA
MG964475	H5N2	05/02/2015	A/chicken/Iowa/15-014591-1/2015	7	M
MG964476	H5N2	05/02/2015	A/chicken/Iowa/15-014591-1/2015	8	NS
MG964477	H5N2	05/05/2015	A/chicken/Iowa/15-014719-1/2015	1	PB2
MG964478	H5N2	05/05/2015	A/chicken/Iowa/15-014719-1/2015	2	PB1
MG964479	H5N2	05/05/2015	A/chicken/Iowa/15-014719-1/2015	3	PA
MG964480	H5N2	05/05/2015	A/chicken/Iowa/15-014719-1/2015	4	HA
MG964481	H5N2	05/05/2015	A/chicken/Iowa/15-014719-1/2015	5	NP
MG964482	H5N2	05/05/2015	A/chicken/Iowa/15-014719-1/2015	6	NA
MG964483	H5N2	05/05/2015	A/chicken/Iowa/15-014719-1/2015	7	M
MG964484	H5N2	05/05/2015	A/chicken/Iowa/15-014719-1/2015	8	NS
MG964485	H5N2	05/05/2015	A/chicken/Iowa/15-014769-1/2015	1	PB2
MG964486	H5N2	05/05/2015	A/chicken/Iowa/15-014769-1/2015	2	PB1
MG964487	H5N2	05/05/2015	A/chicken/Iowa/15-014769-1/2015	3	PA
MG964488	H5N2	05/05/2015	A/chicken/Iowa/15-014769-1/2015	4	HA
MG964489	H5N2	05/05/2015	A/chicken/Iowa/15-014769-1/2015	5	NP
MG964490	H5N2	05/05/2015	A/chicken/Iowa/15-014769-1/2015	6	NA
MG964491	H5N2	05/05/2015	A/chicken/Iowa/15-014769-1/2015	7	M
MG964492	H5N2	05/05/2015	A/chicken/Iowa/15-014769-1/2015	8	NS
MG964493	H5N2	05/05/2015	A/chicken/Iowa/15-014774-1/2015	1	PB2
MG964494	H5N2	05/05/2015	A/chicken/Iowa/15-014774-1/2015	2	PB1
MG964495	H5N2	05/05/2015	A/chicken/Iowa/15-014774-1/2015	3	PA
MG964496	H5N2	05/05/2015	A/chicken/Iowa/15-014774-1/2015	4	HA
MG964497	H5N2	05/05/2015	A/chicken/Iowa/15-014774-1/2015	5	NP

MG964498	H5N2	05/05/2015	A/chicken/Iowa/15-014774-1/2015	6	NA
MG964499	H5N2	05/05/2015	A/chicken/Iowa/15-014774-1/2015	7	M
MG964500	H5N2	05/05/2015	A/chicken/Iowa/15-014774-1/2015	8	NS
MG964501	H5N2	05/07/2015	A/chicken/Iowa/15-015016-1/2015	1	PB2
MG964502	H5N2	05/07/2015	A/chicken/Iowa/15-015016-1/2015	2	PB1
MG964503	H5N2	05/07/2015	A/chicken/Iowa/15-015016-1/2015	3	PA
MG964504	H5N2	05/07/2015	A/chicken/Iowa/15-015016-1/2015	4	HA
MG964505	H5N2	05/07/2015	A/chicken/Iowa/15-015016-1/2015	5	NP
MG964506	H5N2	05/07/2015	A/chicken/Iowa/15-015016-1/2015	6	NA
MG964507	H5N2	05/07/2015	A/chicken/Iowa/15-015016-1/2015	7	M
MG964508	H5N2	05/07/2015	A/chicken/Iowa/15-015016-1/2015	8	NS
MG964509	H5N2	05/09/2015	A/chicken/Iowa/15-015018-1/2015	1	PB2
MG964510	H5N2	05/09/2015	A/chicken/Iowa/15-015018-1/2015	2	PB1
MG964511	H5N2	05/09/2015	A/chicken/Iowa/15-015018-1/2015	3	PA
MG964512	H5N2	05/09/2015	A/chicken/Iowa/15-015018-1/2015	4	HA
MG964513	H5N2	05/09/2015	A/chicken/Iowa/15-015018-1/2015	5	NP
MG964514	H5N2	05/09/2015	A/chicken/Iowa/15-015018-1/2015	6	NA
MG964515	H5N2	05/09/2015	A/chicken/Iowa/15-015018-1/2015	7	M
MG964516	H5N2	05/09/2015	A/chicken/Iowa/15-015018-1/2015	8	NS
MG964517	H5N2	05/10/2015	A/chicken/Iowa/15-015073-1/2015	1	PB2
MG964518	H5N2	05/10/2015	A/chicken/Iowa/15-015073-1/2015	2	PB1
MG964519	H5N2	05/10/2015	A/chicken/Iowa/15-015073-1/2015	3	PA
MG964520	H5N2	05/10/2015	A/chicken/Iowa/15-015073-1/2015	4	HA
MG964521	H5N2	05/10/2015	A/chicken/Iowa/15-015073-1/2015	5	NP
MG964522	H5N2	05/10/2015	A/chicken/Iowa/15-015073-1/2015	6	NA
MG964523	H5N2	05/10/2015	A/chicken/Iowa/15-015073-1/2015	7	M
MG964524	H5N2	05/10/2015	A/chicken/Iowa/15-015073-1/2015	8	NS
MG964525	H5N2	05/11/2015	A/chicken/Iowa/15-015117-1/2015	1	PB2
MG964526	H5N2	05/11/2015	A/chicken/Iowa/15-015117-1/2015	2	PB1
MG964527	H5N2	05/11/2015	A/chicken/Iowa/15-015117-1/2015	3	PA
MG964528	H5N2	05/11/2015	A/chicken/Iowa/15-015117-1/2015	4	HA
MG964529	H5N2	05/11/2015	A/chicken/Iowa/15-015117-1/2015	5	NP
MG964530	H5N2	05/11/2015	A/chicken/Iowa/15-015117-1/2015	6	NA
MG964531	H5N2	05/11/2015	A/chicken/Iowa/15-015117-1/2015	7	M
MG964532	H5N2	05/11/2015	A/chicken/Iowa/15-015117-1/2015	8	NS
MG964533	H5N2	05/11/2015	A/chicken/Iowa/15-015300-1/2015	1	PB2
MG964534	H5N2	05/11/2015	A/chicken/Iowa/15-015300-1/2015	2	PB1
MG964535	H5N2	05/11/2015	A/chicken/Iowa/15-015300-1/2015	3	PA
MG964536	H5N2	05/11/2015	A/chicken/Iowa/15-015300-1/2015	4	HA
MG964537	H5N2	05/11/2015	A/chicken/Iowa/15-015300-1/2015	5	NP
MG964538	H5N2	05/11/2015	A/chicken/Iowa/15-015300-1/2015	6	NA
MG964539	H5N2	05/11/2015	A/chicken/Iowa/15-015300-1/2015	7	M
MG964540	H5N2	05/11/2015	A/chicken/Iowa/15-015300-1/2015	8	NS
MG964541	H5N2	05/12/2015	A/chicken/Iowa/15-015544-2/2015	1	PB2
MG964542	H5N2	05/12/2015	A/chicken/Iowa/15-015544-2/2015	2	PB1
MG964543	H5N2	05/12/2015	A/chicken/Iowa/15-015544-2/2015	3	PA
MG964544	H5N2	05/12/2015	A/chicken/Iowa/15-015544-2/2015	4	HA
MG964545	H5N2	05/12/2015	A/chicken/Iowa/15-015544-2/2015	5	NP
MG964546	H5N2	05/12/2015	A/chicken/Iowa/15-015544-2/2015	6	NA
MG964547	H5N2	05/12/2015	A/chicken/Iowa/15-015544-2/2015	7	M
MG964548	H5N2	05/12/2015	A/chicken/Iowa/15-015544-2/2015	8	NS
MG964549	H5N2	05/12/2015	A/chicken/Iowa/15-015555-1/2015	1	PB2
MG964550	H5N2	05/12/2015	A/chicken/Iowa/15-015555-1/2015	2	PB1
MG964551	H5N2	05/12/2015	A/chicken/Iowa/15-015555-1/2015	3	PA
MG964552	H5N2	05/12/2015	A/chicken/Iowa/15-015555-1/2015	4	HA
MG964553	H5N2	05/12/2015	A/chicken/Iowa/15-015555-1/2015	5	NP
MG964554	H5N2	05/12/2015	A/chicken/Iowa/15-015555-1/2015	6	NA
MG964555	H5N2	05/12/2015	A/chicken/Iowa/15-015555-1/2015	7	M
MG964556	H5N2	05/12/2015	A/chicken/Iowa/15-015555-1/2015	8	NS
MG964557	H5N2	05/12/2015	A/chicken/Iowa/15-015556-1/2015	1	PB2
MG964558	H5N2	05/12/2015	A/chicken/Iowa/15-015556-1/2015	2	PB1
MG964559	H5N2	05/12/2015	A/chicken/Iowa/15-015556-1/2015	3	PA
MG964560	H5N2	05/12/2015	A/chicken/Iowa/15-015556-1/2015	4	HA
MG964561	H5N2	05/12/2015	A/chicken/Iowa/15-015556-1/2015	5	NP
MG964562	H5N2	05/12/2015	A/chicken/Iowa/15-015556-1/2015	6	NA
MG964563	H5N2	05/12/2015	A/chicken/Iowa/15-015556-1/2015	7	M
MG964564	H5N2	05/12/2015	A/chicken/Iowa/15-015556-1/2015	8	NS
MG964565	H5N2	05/14/2015	A/chicken/Iowa/15-016078-1/2015	1	PB2
MG964566	H5N2	05/14/2015	A/chicken/Iowa/15-016078-1/2015	2	PB1
MG964567	H5N2	05/14/2015	A/chicken/Iowa/15-016078-1/2015	3	PA
MG964568	H5N2	05/14/2015	A/chicken/Iowa/15-016078-1/2015	4	HA
MG964569	H5N2	05/14/2015	A/chicken/Iowa/15-016078-1/2015	5	NP
MG964570	H5N2	05/14/2015	A/chicken/Iowa/15-016078-1/2015	6	NA
MG964571	H5N2	05/14/2015	A/chicken/Iowa/15-016078-1/2015	7	M
MG964572	H5N2	05/14/2015	A/chicken/Iowa/15-016078-1/2015	8	NS
MG964573	H5N2	05/14/2015	A/chicken/Iowa/15-016158-1/2015	1	PB2
MG964574	H5N2	05/14/2015	A/chicken/Iowa/15-016158-1/2015	2	PB1
MG964575	H5N2	05/14/2015	A/chicken/Iowa/15-016158-1/2015	3	PA
MG964576	H5N2	05/14/2015	A/chicken/Iowa/15-016158-1/2015	4	HA
MG964577	H5N2	05/14/2015	A/chicken/Iowa/15-016158-1/2015	5	NP
MG964578	H5N2	05/14/2015	A/chicken/Iowa/15-016158-1/2015	6	NA
MG964579	H5N2	05/14/2015	A/chicken/Iowa/15-016158-1/2015	7	M
MG964580	H5N2	05/14/2015	A/chicken/Iowa/15-016158-1/2015	8	NS
MG964581	H5N2	05/18/2015	A/chicken/Iowa/15-016159-1/2015	1	PB2
MG964582	H5N2	05/18/2015	A/chicken/Iowa/15-016159-1/2015	2	PB1
MG964583	H5N2	05/18/2015	A/chicken/Iowa/15-016159-1/2015	3	PA
MG964584	H5N2	05/18/2015	A/chicken/Iowa/15-016159-1/2015	4	HA
MG964585	H5N2	05/18/2015	A/chicken/Iowa/15-016159-1/2015	5	NP
MG964586	H5N2	05/18/2015	A/chicken/Iowa/15-016159-1/2015	6	NA
MG964587	H5N2	05/18/2015	A/chicken/Iowa/15-016159-1/2015	7	M

MG964588	H5N2	05/18/2015	A/chicken/Iowa/15-016159-1/2015	8	NS
MG964589	H5N2	05/18/2015	A/chicken/Iowa/15-016160-1/2015	1	PB2
MG964590	H5N2	05/18/2015	A/chicken/Iowa/15-016160-1/2015	2	PB1
MG964591	H5N2	05/18/2015	A/chicken/Iowa/15-016160-1/2015	3	PA
MG964592	H5N2	05/18/2015	A/chicken/Iowa/15-016160-1/2015	4	HA
MG964593	H5N2	05/18/2015	A/chicken/Iowa/15-016160-1/2015	5	NP
MG964594	H5N2	05/18/2015	A/chicken/Iowa/15-016160-1/2015	6	NA
MG964595	H5N2	05/18/2015	A/chicken/Iowa/15-016160-1/2015	7	M
MG964596	H5N2	05/18/2015	A/chicken/Iowa/15-016160-1/2015	8	NS
MG964597	H5N2	05/24/2015	A/chicken/Iowa/15-016887-1/2015	1	PB2
MG964598	H5N2	05/24/2015	A/chicken/Iowa/15-016887-1/2015	2	PB1
MG964599	H5N2	05/24/2015	A/chicken/Iowa/15-016887-1/2015	3	PA
MG964600	H5N2	05/24/2015	A/chicken/Iowa/15-016887-1/2015	4	HA
MG964601	H5N2	05/24/2015	A/chicken/Iowa/15-016887-1/2015	5	NP
MG964602	H5N2	05/24/2015	A/chicken/Iowa/15-016887-1/2015	6	NA
MG964603	H5N2	05/24/2015	A/chicken/Iowa/15-016887-1/2015	7	M
MG964604	H5N2	05/24/2015	A/chicken/Iowa/15-016887-1/2015	8	NS
MG964605	H5N2	05/25/2015	A/chicken/Iowa/15-016888-1/2015	1	PB2
MG964606	H5N2	05/25/2015	A/chicken/Iowa/15-016888-1/2015	2	PB1
MG964607	H5N2	05/25/2015	A/chicken/Iowa/15-016888-1/2015	3	PA
MG964608	H5N2	05/25/2015	A/chicken/Iowa/15-016888-1/2015	4	HA
MG964609	H5N2	05/25/2015	A/chicken/Iowa/15-016888-1/2015	5	NP
MG964610	H5N2	05/25/2015	A/chicken/Iowa/15-016888-1/2015	6	NA
MG964611	H5N2	05/25/2015	A/chicken/Iowa/15-016888-1/2015	7	M
MG964612	H5N2	05/25/2015	A/chicken/Iowa/15-016888-1/2015	8	NS
MG964613	H5N2	05/25/2015	A/chicken/Iowa/15-017038-1/2015	1	PB2
MG964614	H5N2	05/25/2015	A/chicken/Iowa/15-017038-1/2015	2	PB1
MG964615	H5N2	05/25/2015	A/chicken/Iowa/15-017038-1/2015	3	PA
MG964616	H5N2	05/25/2015	A/chicken/Iowa/15-017038-1/2015	4	HA
MG964617	H5N2	05/25/2015	A/chicken/Iowa/15-017038-1/2015	5	NP
MG964618	H5N2	05/25/2015	A/chicken/Iowa/15-017038-1/2015	6	NA
MG964619	H5N2	05/25/2015	A/chicken/Iowa/15-017038-1/2015	7	M
MG964620	H5N2	05/25/2015	A/chicken/Iowa/15-017038-1/2015	8	NS
MG964621	H5N2	05/27/2015	A/chicken/Iowa/15-017419-1/2015	1	PB2
MG964622	H5N2	05/27/2015	A/chicken/Iowa/15-017419-1/2015	2	PB1
MG964623	H5N2	05/27/2015	A/chicken/Iowa/15-017419-1/2015	3	PA
MG964624	H5N2	05/27/2015	A/chicken/Iowa/15-017419-1/2015	4	HA
MG964625	H5N2	05/27/2015	A/chicken/Iowa/15-017419-1/2015	5	NP
MG964626	H5N2	05/27/2015	A/chicken/Iowa/15-017419-1/2015	6	NA
MG964627	H5N2	05/27/2015	A/chicken/Iowa/15-017419-1/2015	7	M
MG964628	H5N2	05/27/2015	A/chicken/Iowa/15-017419-1/2015	8	NS
MG964629	H5N2	06/01/2015	A/chicken/Iowa/15-017656-1/2015	1	PB2
MG964630	H5N2	06/01/2015	A/chicken/Iowa/15-017656-1/2015	2	PB1
MG964631	H5N2	06/01/2015	A/chicken/Iowa/15-017656-1/2015	3	PA
MG964632	H5N2	06/01/2015	A/chicken/Iowa/15-017656-1/2015	4	HA
MG964633	H5N2	06/01/2015	A/chicken/Iowa/15-017656-1/2015	5	NP
MG964634	H5N2	06/01/2015	A/chicken/Iowa/15-017656-1/2015	6	NA
MG964635	H5N2	06/01/2015	A/chicken/Iowa/15-017656-1/2015	7	M
MG964636	H5N2	06/01/2015	A/chicken/Iowa/15-017656-1/2015	8	NS
MG964637	H5N2	06/15/2015	A/chicken/Iowa/15-019293-1/2015	1	PB2
MG964638	H5N2	06/15/2015	A/chicken/Iowa/15-019293-1/2015	2	PB1
MG964639	H5N2	06/15/2015	A/chicken/Iowa/15-019293-1/2015	3	PA
MG964640	H5N2	06/15/2015	A/chicken/Iowa/15-019293-1/2015	4	HA
MG964641	H5N2	06/15/2015	A/chicken/Iowa/15-019293-1/2015	5	NP
MG964642	H5N2	06/15/2015	A/chicken/Iowa/15-019293-1/2015	6	NA
MG964643	H5N2	06/15/2015	A/chicken/Iowa/15-019293-1/2015	7	M
MG964644	H5N2	06/15/2015	A/chicken/Iowa/15-019293-1/2015	8	NS
MG964661	H5N2	05/01/2015	A/chicken/Minnesota/15-014297-1/2015	1	PB2
MG964662	H5N2	05/01/2015	A/chicken/Minnesota/15-014297-1/2015	2	PB1
MG964663	H5N2	05/01/2015	A/chicken/Minnesota/15-014297-1/2015	3	PA
MG964664	H5N2	05/01/2015	A/chicken/Minnesota/15-014297-1/2015	4	HA
MG964665	H5N2	05/01/2015	A/chicken/Minnesota/15-014297-1/2015	5	NP
MG964666	H5N2	05/01/2015	A/chicken/Minnesota/15-014297-1/2015	6	NA
MG964667	H5N2	05/01/2015	A/chicken/Minnesota/15-014297-1/2015	7	M
MG964668	H5N2	05/01/2015	A/chicken/Minnesota/15-014297-1/2015	8	NS
MG964669	H5N2	05/13/2015	A/chicken/Minnesota/15-015844-1/2015	1	PB2
MG964670	H5N2	05/13/2015	A/chicken/Minnesota/15-015844-1/2015	2	PB1
MG964671	H5N2	05/13/2015	A/chicken/Minnesota/15-015844-1/2015	3	PA
MG964672	H5N2	05/13/2015	A/chicken/Minnesota/15-015844-1/2015	4	HA
MG964673	H5N2	05/13/2015	A/chicken/Minnesota/15-015844-1/2015	5	NP
MG964674	H5N2	05/13/2015	A/chicken/Minnesota/15-015844-1/2015	6	NA
MG964675	H5N2	05/13/2015	A/chicken/Minnesota/15-015844-1/2015	7	M
MG964676	H5N2	05/13/2015	A/chicken/Minnesota/15-015844-1/2015	8	NS
MG964677	H5N2	06/01/2015	A/chicken/Minnesota/15-017817-1/2015	1	PB2
MG964678	H5N2	06/01/2015	A/chicken/Minnesota/15-017817-1/2015	2	PB1
MG964679	H5N2	06/01/2015	A/chicken/Minnesota/15-017817-1/2015	3	PA
MG964680	H5N2	06/01/2015	A/chicken/Minnesota/15-017817-1/2015	4	HA
MG964681	H5N2	06/01/2015	A/chicken/Minnesota/15-017817-1/2015	5	NP
MG964682	H5N2	06/01/2015	A/chicken/Minnesota/15-017817-1/2015	6	NA
MG964683	H5N2	06/01/2015	A/chicken/Minnesota/15-017817-1/2015	7	M
MG964684	H5N2	06/01/2015	A/chicken/Minnesota/15-017817-1/2015	8	NS
MG964693	H5N2	05/11/2015	A/chicken/Nebraska/15-015085-1/2015	1	PB2
MG964694	H5N2	05/11/2015	A/chicken/Nebraska/15-015085-1/2015	2	PB1
MG964695	H5N2	05/11/2015	A/chicken/Nebraska/15-015085-1/2015	3	PA
MG964696	H5N2	05/11/2015	A/chicken/Nebraska/15-015085-1/2015	4	HA
MG964697	H5N2	05/11/2015	A/chicken/Nebraska/15-015085-1/2015	5	NP
MG964698	H5N2	05/11/2015	A/chicken/Nebraska/15-015085-1/2015	6	NA
MG964699	H5N2	05/11/2015	A/chicken/Nebraska/15-015085-1/2015	7	M
MG964700	H5N2	05/11/2015	A/chicken/Nebraska/15-015085-1/2015	8	NS
MG964701	H5N2	05/12/2015	A/chicken/Nebraska/15-015543-1/2015	1	PB2

MG964702	H5N2	05/12/2015	A/chicken/Nebraska/15-015543-1/2015	2	PB1
MG964703	H5N2	05/12/2015	A/chicken/Nebraska/15-015543-1/2015	3	PA
MG964704	H5N2	05/12/2015	A/chicken/Nebraska/15-015543-1/2015	4	HA
MG964705	H5N2	05/12/2015	A/chicken/Nebraska/15-015543-1/2015	5	NP
MG964706	H5N2	05/12/2015	A/chicken/Nebraska/15-015543-1/2015	6	NA
MG964707	H5N2	05/12/2015	A/chicken/Nebraska/15-015543-1/2015	7	M
MG964708	H5N2	05/12/2015	A/chicken/Nebraska/15-015543-1/2015	8	NS
MG964709	H5N2	05/21/2015	A/chicken/Nebraska/15-016865-1/2015	1	PB2
MG964710	H5N2	05/21/2015	A/chicken/Nebraska/15-016865-1/2015	2	PB1
MG964711	H5N2	05/21/2015	A/chicken/Nebraska/15-016865-1/2015	3	PA
MG964712	H5N2	05/21/2015	A/chicken/Nebraska/15-016865-1/2015	4	HA
MG964713	H5N2	05/21/2015	A/chicken/Nebraska/15-016865-1/2015	5	NP
MG964714	H5N2	05/21/2015	A/chicken/Nebraska/15-016865-1/2015	6	NA
MG964715	H5N2	05/21/2015	A/chicken/Nebraska/15-016865-1/2015	7	M
MG964716	H5N2	05/21/2015	A/chicken/Nebraska/15-016865-1/2015	8	NS
MG964717	H5N2	06/11/2015	A/chicken/Nebraska/15-019197-1/2015	1	PB2
MG964718	H5N2	06/11/2015	A/chicken/Nebraska/15-019197-1/2015	2	PB1
MG964719	H5N2	06/11/2015	A/chicken/Nebraska/15-019197-1/2015	3	PA
MG964720	H5N2	06/11/2015	A/chicken/Nebraska/15-019197-1/2015	4	HA
MG964721	H5N2	06/11/2015	A/chicken/Nebraska/15-019197-1/2015	5	NP
MG964722	H5N2	06/11/2015	A/chicken/Nebraska/15-019197-1/2015	6	NA
MG964723	H5N2	06/11/2015	A/chicken/Nebraska/15-019197-1/2015	7	M
MG964724	H5N2	06/11/2015	A/chicken/Nebraska/15-019197-1/2015	8	NS
MG964733	H5N2	05/13/2015	A/chicken/South Dakota/15-015847-3/2015	1	PB2
MG964734	H5N2	05/13/2015	A/chicken/South Dakota/15-015847-3/2015	2	PB1
MG964735	H5N2	05/13/2015	A/chicken/South Dakota/15-015847-3/2015	3	PA
MG964736	H5N2	05/13/2015	A/chicken/South Dakota/15-015847-3/2015	4	HA
MG964737	H5N2	05/13/2015	A/chicken/South Dakota/15-015847-3/2015	5	NP
MG964738	H5N2	05/13/2015	A/chicken/South Dakota/15-015847-3/2015	6	NA
MG964739	H5N2	05/13/2015	A/chicken/South Dakota/15-015847-3/2015	7	M
MG964740	H5N2	05/13/2015	A/chicken/South Dakota/15-015847-3/2015	8	NS
MG964741	H5N2	04/21/2015	A/chicken/Wisconsin/15-013062-1/2015	1	PB2
MG964742	H5N2	04/21/2015	A/chicken/Wisconsin/15-013062-1/2015	2	PB1
MG964743	H5N2	04/21/2015	A/chicken/Wisconsin/15-013062-1/2015	3	PA
MG964744	H5N2	04/21/2015	A/chicken/Wisconsin/15-013062-1/2015	4	HA
MG964745	H5N2	04/21/2015	A/chicken/Wisconsin/15-013062-1/2015	5	NP
MG964746	H5N2	04/21/2015	A/chicken/Wisconsin/15-013062-1/2015	6	NA
MG964747	H5N2	04/21/2015	A/chicken/Wisconsin/15-013062-1/2015	7	M
MG964748	H5N2	04/21/2015	A/chicken/Wisconsin/15-013062-1/2015	8	NS
MG964749	H5N2	05/04/2015	A/chicken/Wisconsin/15-014400-1/2015	1	PB2
MG964750	H5N2	05/04/2015	A/chicken/Wisconsin/15-014400-1/2015	2	PB1
MG964751	H5N2	05/04/2015	A/chicken/Wisconsin/15-014400-1/2015	3	PA
MG964752	H5N2	05/04/2015	A/chicken/Wisconsin/15-014400-1/2015	4	HA
MG964753	H5N2	05/04/2015	A/chicken/Wisconsin/15-014400-1/2015	5	NP
MG964754	H5N2	05/04/2015	A/chicken/Wisconsin/15-014400-1/2015	6	NA
MG964755	H5N2	05/04/2015	A/chicken/Wisconsin/15-014400-1/2015	7	M
MG964756	H5N2	05/04/2015	A/chicken/Wisconsin/15-014400-1/2015	8	NS
MG964829	H5N2	2015	A/turkey/Iowa/15-013179-4/2015	1	PB2
MG964830	H5N2	2015	A/turkey/Iowa/15-013179-4/2015	2	PB1
MG964831	H5N2	2015	A/turkey/Iowa/15-013179-4/2015	3	PA
MG964832	H5N2	2015	A/turkey/Iowa/15-013179-4/2015	4	HA
MG964833	H5N2	2015	A/turkey/Iowa/15-013179-4/2015	5	NP
MG964834	H5N2	2015	A/turkey/Iowa/15-013179-4/2015	6	NA
MG964835	H5N2	2015	A/turkey/Iowa/15-013179-4/2015	7	M
MG964836	H5N2	2015	A/turkey/Iowa/15-013179-4/2015	8	NS
MG964837	H5N2	04/27/2015	A/turkey/Iowa/15-013773-1/2015	1	PB2
MG964838	H5N2	04/27/2015	A/turkey/Iowa/15-013773-1/2015	2	PB1
MG964839	H5N2	04/27/2015	A/turkey/Iowa/15-013773-1/2015	3	PA
MG964840	H5N2	04/27/2015	A/turkey/Iowa/15-013773-1/2015	4	HA
MG964841	H5N2	04/27/2015	A/turkey/Iowa/15-013773-1/2015	5	NP
MG964842	H5N2	04/27/2015	A/turkey/Iowa/15-013773-1/2015	6	NA
MG964843	H5N2	04/27/2015	A/turkey/Iowa/15-013773-1/2015	7	M
MG964844	H5N2	04/27/2015	A/turkey/Iowa/15-013773-1/2015	8	NS
MG964845	H5N2	04/29/2015	A/turkey/Iowa/15-013926-1/2015	1	PB2
MG964846	H5N2	04/29/2015	A/turkey/Iowa/15-013926-1/2015	2	PB1
MG964847	H5N2	04/29/2015	A/turkey/Iowa/15-013926-1/2015	3	PA
MG964848	H5N2	04/29/2015	A/turkey/Iowa/15-013926-1/2015	4	HA
MG964849	H5N2	04/29/2015	A/turkey/Iowa/15-013926-1/2015	5	NP
MG964850	H5N2	04/29/2015	A/turkey/Iowa/15-013926-1/2015	6	NA
MG964851	H5N2	04/29/2015	A/turkey/Iowa/15-013926-1/2015	7	M
MG964852	H5N2	04/29/2015	A/turkey/Iowa/15-013926-1/2015	8	NS
MG964853	H5N2	04/29/2015	A/turkey/Iowa/15-014119-1/2015	1	PB2
MG964854	H5N2	04/29/2015	A/turkey/Iowa/15-014119-1/2015	2	PB1
MG964855	H5N2	04/29/2015	A/turkey/Iowa/15-014119-1/2015	3	PA
MG964856	H5N2	04/29/2015	A/turkey/Iowa/15-014119-1/2015	4	HA
MG964857	H5N2	04/29/2015	A/turkey/Iowa/15-014119-1/2015	5	NP
MG964858	H5N2	04/29/2015	A/turkey/Iowa/15-014119-1/2015	6	NA
MG964859	H5N2	04/29/2015	A/turkey/Iowa/15-014119-1/2015	7	M
MG964860	H5N2	04/29/2015	A/turkey/Iowa/15-014119-1/2015	8	NS
MG964861	H5N2	04/30/2015	A/turkey/Iowa/15-014120-1/2015	1	PB2
MG964862	H5N2	04/30/2015	A/turkey/Iowa/15-014120-1/2015	2	PB1
MG964863	H5N2	04/30/2015	A/turkey/Iowa/15-014120-1/2015	3	PA
MG964864	H5N2	04/30/2015	A/turkey/Iowa/15-014120-1/2015	4	HA
MG964865	H5N2	04/30/2015	A/turkey/Iowa/15-014120-1/2015	5	NP
MG964866	H5N2	04/30/2015	A/turkey/Iowa/15-014120-1/2015	6	NA
MG964867	H5N2	04/30/2015	A/turkey/Iowa/15-014120-1/2015	7	M
MG964868	H5N2	04/30/2015	A/turkey/Iowa/15-014120-1/2015	8	NS
MG964869	H5N2	05/02/2015	A/turkey/Iowa/15-014562-2/2015	1	PB2
MG964870	H5N2	05/02/2015	A/turkey/Iowa/15-014562-2/2015	2	PB1
MG964871	H5N2	05/02/2015	A/turkey/Iowa/15-014562-2/2015	3	PA

MG964872	H5N2	05/02/2015	A/turkey/Iowa/15-014562-2/2015	4	HA
MG964873	H5N2	05/02/2015	A/turkey/Iowa/15-014562-2/2015	5	NP
MG964874	H5N2	05/02/2015	A/turkey/Iowa/15-014562-2/2015	6	NA
MG964875	H5N2	05/02/2015	A/turkey/Iowa/15-014562-2/2015	7	M
MG964876	H5N2	05/02/2015	A/turkey/Iowa/15-014562-2/2015	8	NS
MG964877	H5N2	05/03/2015	A/turkey/Iowa/15-014570-1/2015	1	PB2
MG964878	H5N2	05/03/2015	A/turkey/Iowa/15-014570-1/2015	2	PB1
MG964879	H5N2	05/03/2015	A/turkey/Iowa/15-014570-1/2015	3	PA
MG964880	H5N2	05/03/2015	A/turkey/Iowa/15-014570-1/2015	4	HA
MG964881	H5N2	05/03/2015	A/turkey/Iowa/15-014570-1/2015	5	NP
MG964882	H5N2	05/03/2015	A/turkey/Iowa/15-014570-1/2015	6	NA
MG964883	H5N2	05/03/2015	A/turkey/Iowa/15-014570-1/2015	7	M
MG964884	H5N2	05/03/2015	A/turkey/Iowa/15-014570-1/2015	8	NS
MG964885	H5N2	05/05/2015	A/turkey/Iowa/15-014772-1/2015	1	PB2
MG964886	H5N2	05/05/2015	A/turkey/Iowa/15-014772-1/2015	2	PB1
MG964887	H5N2	05/05/2015	A/turkey/Iowa/15-014772-1/2015	3	PA
MG964888	H5N2	05/05/2015	A/turkey/Iowa/15-014772-1/2015	4	HA
MG964889	H5N2	05/05/2015	A/turkey/Iowa/15-014772-1/2015	5	NP
MG964890	H5N2	05/05/2015	A/turkey/Iowa/15-014772-1/2015	6	NA
MG964891	H5N2	05/05/2015	A/turkey/Iowa/15-014772-1/2015	7	M
MG964892	H5N2	05/05/2015	A/turkey/Iowa/15-014772-1/2015	8	NS
MG964893	H5N2	05/05/2015	A/turkey/Iowa/15-014789-1/2015	1	PB2
MG964894	H5N2	05/05/2015	A/turkey/Iowa/15-014789-1/2015	2	PB1
MG964895	H5N2	05/05/2015	A/turkey/Iowa/15-014789-1/2015	3	PA
MG964896	H5N2	05/05/2015	A/turkey/Iowa/15-014789-1/2015	4	HA
MG964897	H5N2	05/05/2015	A/turkey/Iowa/15-014789-1/2015	5	NP
MG964898	H5N2	05/05/2015	A/turkey/Iowa/15-014789-1/2015	6	NA
MG964899	H5N2	05/05/2015	A/turkey/Iowa/15-014789-1/2015	7	M
MG964900	H5N2	05/05/2015	A/turkey/Iowa/15-014789-1/2015	8	NS
MG964901	H5N2	05/06/2015	A/turkey/Iowa/15-014862-1/2015	1	PB2
MG964902	H5N2	05/06/2015	A/turkey/Iowa/15-014862-1/2015	2	PB1
MG964903	H5N2	05/06/2015	A/turkey/Iowa/15-014862-1/2015	3	PA
MG964904	H5N2	05/06/2015	A/turkey/Iowa/15-014862-1/2015	4	HA
MG964905	H5N2	05/06/2015	A/turkey/Iowa/15-014862-1/2015	5	NP
MG964906	H5N2	05/06/2015	A/turkey/Iowa/15-014862-1/2015	6	NA
MG964907	H5N2	05/06/2015	A/turkey/Iowa/15-014862-1/2015	7	M
MG964908	H5N2	05/06/2015	A/turkey/Iowa/15-014862-1/2015	8	NS
MG964909	H5N2	05/06/2015	A/turkey/Iowa/15-014863-1/2015	1	PB2
MG964910	H5N2	05/06/2015	A/turkey/Iowa/15-014863-1/2015	2	PB1
MG964911	H5N2	05/06/2015	A/turkey/Iowa/15-014863-1/2015	3	PA
MG964912	H5N2	05/06/2015	A/turkey/Iowa/15-014863-1/2015	4	HA
MG964913	H5N2	05/06/2015	A/turkey/Iowa/15-014863-1/2015	5	NP
MG964914	H5N2	05/06/2015	A/turkey/Iowa/15-014863-1/2015	6	NA
MG964915	H5N2	05/06/2015	A/turkey/Iowa/15-014863-1/2015	7	M
MG964916	H5N2	05/06/2015	A/turkey/Iowa/15-014863-1/2015	8	NS
MG964917	H5N2	05/06/2015	A/turkey/Iowa/15-014864-1/2015	1	PB2
MG964918	H5N2	05/06/2015	A/turkey/Iowa/15-014864-1/2015	2	PB1
MG964919	H5N2	05/06/2015	A/turkey/Iowa/15-014864-1/2015	3	PA
MG964920	H5N2	05/06/2015	A/turkey/Iowa/15-014864-1/2015	4	HA
MG964921	H5N2	05/06/2015	A/turkey/Iowa/15-014864-1/2015	5	NP
MG964922	H5N2	05/06/2015	A/turkey/Iowa/15-014864-1/2015	6	NA
MG964923	H5N2	05/06/2015	A/turkey/Iowa/15-014864-1/2015	7	M
MG964924	H5N2	05/06/2015	A/turkey/Iowa/15-014864-1/2015	8	NS
MG964925	H5N2	05/07/2015	A/turkey/Iowa/15-014868-1/2015	1	PB2
MG964926	H5N2	05/07/2015	A/turkey/Iowa/15-014868-1/2015	2	PB1
MG964927	H5N2	05/07/2015	A/turkey/Iowa/15-014868-1/2015	3	PA
MG964928	H5N2	05/07/2015	A/turkey/Iowa/15-014868-1/2015	4	HA
MG964929	H5N2	05/07/2015	A/turkey/Iowa/15-014868-1/2015	5	NP
MG964930	H5N2	05/07/2015	A/turkey/Iowa/15-014868-1/2015	6	NA
MG964931	H5N2	05/07/2015	A/turkey/Iowa/15-014868-1/2015	7	M
MG964932	H5N2	05/07/2015	A/turkey/Iowa/15-014868-1/2015	8	NS
MG964933	H5N2	05/07/2015	A/turkey/Iowa/15-014869-1/2015	1	PB2
MG964934	H5N2	05/07/2015	A/turkey/Iowa/15-014869-1/2015	2	PB1
MG964935	H5N2	05/07/2015	A/turkey/Iowa/15-014869-1/2015	3	PA
MG964936	H5N2	05/07/2015	A/turkey/Iowa/15-014869-1/2015	4	HA
MG964937	H5N2	05/07/2015	A/turkey/Iowa/15-014869-1/2015	5	NP
MG964938	H5N2	05/07/2015	A/turkey/Iowa/15-014869-1/2015	6	NA
MG964939	H5N2	05/07/2015	A/turkey/Iowa/15-014869-1/2015	7	M
MG964940	H5N2	05/07/2015	A/turkey/Iowa/15-014869-1/2015	8	NS
MG964941	H5N2	05/08/2015	A/turkey/Iowa/15-014954-1/2015	1	PB2
MG964942	H5N2	05/08/2015	A/turkey/Iowa/15-014954-1/2015	2	PB1
MG964943	H5N2	05/08/2015	A/turkey/Iowa/15-014954-1/2015	3	PA
MG964944	H5N2	05/08/2015	A/turkey/Iowa/15-014954-1/2015	4	HA
MG964945	H5N2	05/08/2015	A/turkey/Iowa/15-014954-1/2015	5	NP
MG964946	H5N2	05/08/2015	A/turkey/Iowa/15-014954-1/2015	6	NA
MG964947	H5N2	05/08/2015	A/turkey/Iowa/15-014954-1/2015	7	M
MG964948	H5N2	05/08/2015	A/turkey/Iowa/15-014954-1/2015	8	NS
MG964949	H5N2	05/10/2015	A/turkey/Iowa/15-015071-3/2015	1	PB2
MG964950	H5N2	05/10/2015	A/turkey/Iowa/15-015071-3/2015	2	PB1
MG964951	H5N2	05/10/2015	A/turkey/Iowa/15-015071-3/2015	3	PA
MG964952	H5N2	05/10/2015	A/turkey/Iowa/15-015071-3/2015	4	HA
MG964953	H5N2	05/10/2015	A/turkey/Iowa/15-015071-3/2015	5	NP
MG964954	H5N2	05/10/2015	A/turkey/Iowa/15-015071-3/2015	6	NA
MG964955	H5N2	05/10/2015	A/turkey/Iowa/15-015071-3/2015	7	M
MG964956	H5N2	05/10/2015	A/turkey/Iowa/15-015071-3/2015	8	NS
MG964957	H5N2	05/10/2015	A/turkey/Iowa/15-015116-1/2015	1	PB2
MG964958	H5N2	05/10/2015	A/turkey/Iowa/15-015116-1/2015	2	PB1
MG964959	H5N2	05/10/2015	A/turkey/Iowa/15-015116-1/2015	3	PA
MG964960	H5N2	05/10/2015	A/turkey/Iowa/15-015116-1/2015	4	HA
MG964961	H5N2	05/10/2015	A/turkey/Iowa/15-015116-1/2015	5	NP

MG964962	H5N2	05/10/2015	A/turkey/Iowa/15-015116-1/2015	6	NA
MG964963	H5N2	05/10/2015	A/turkey/Iowa/15-015116-1/2015	7	M
MG964964	H5N2	05/10/2015	A/turkey/Iowa/15-015116-1/2015	8	NS
MG964965	H5N2	05/15/2015	A/turkey/Iowa/15-016077-1/2015	1	PB2
MG964966	H5N2	05/15/2015	A/turkey/Iowa/15-016077-1/2015	2	PB1
MG964967	H5N2	05/15/2015	A/turkey/Iowa/15-016077-1/2015	3	PA
MG964968	H5N2	05/15/2015	A/turkey/Iowa/15-016077-1/2015	4	HA
MG964969	H5N2	05/15/2015	A/turkey/Iowa/15-016077-1/2015	5	NP
MG964970	H5N2	05/15/2015	A/turkey/Iowa/15-016077-1/2015	6	NA
MG964971	H5N2	05/15/2015	A/turkey/Iowa/15-016077-1/2015	7	M
MG964972	H5N2	05/15/2015	A/turkey/Iowa/15-016077-1/2015	8	NS
MG964973	H5N2	05/15/2015	A/turkey/Iowa/15-016082-1/2015	1	PB2
MG964974	H5N2	05/15/2015	A/turkey/Iowa/15-016082-1/2015	2	PB1
MG964975	H5N2	05/15/2015	A/turkey/Iowa/15-016082-1/2015	3	PA
MG964976	H5N2	05/15/2015	A/turkey/Iowa/15-016082-1/2015	4	HA
MG964977	H5N2	05/15/2015	A/turkey/Iowa/15-016082-1/2015	5	NP
MG964978	H5N2	05/15/2015	A/turkey/Iowa/15-016082-1/2015	6	NA
MG964979	H5N2	05/15/2015	A/turkey/Iowa/15-016082-1/2015	7	M
MG964980	H5N2	05/15/2015	A/turkey/Iowa/15-016082-1/2015	8	NS
MG964981	H5N2	05/19/2015	A/turkey/Iowa/15-016365-1/2015	1	PB2
MG964982	H5N2	05/19/2015	A/turkey/Iowa/15-016365-1/2015	2	PB1
MG964983	H5N2	05/19/2015	A/turkey/Iowa/15-016365-1/2015	3	PA
MG964984	H5N2	05/19/2015	A/turkey/Iowa/15-016365-1/2015	4	HA
MG964985	H5N2	05/19/2015	A/turkey/Iowa/15-016365-1/2015	5	NP
MG964986	H5N2	05/19/2015	A/turkey/Iowa/15-016365-1/2015	6	NA
MG964987	H5N2	05/19/2015	A/turkey/Iowa/15-016365-1/2015	7	M
MG964988	H5N2	05/19/2015	A/turkey/Iowa/15-016365-1/2015	8	NS
MG964989	H5N2	05/20/2015	A/turkey/Iowa/15-016743-1/2015	1	PB2
MG964990	H5N2	05/20/2015	A/turkey/Iowa/15-016743-1/2015	2	PB1
MG964991	H5N2	05/20/2015	A/turkey/Iowa/15-016743-1/2015	3	PA
MG964992	H5N2	05/20/2015	A/turkey/Iowa/15-016743-1/2015	4	HA
MG964993	H5N2	05/20/2015	A/turkey/Iowa/15-016743-1/2015	5	NP
MG964994	H5N2	05/20/2015	A/turkey/Iowa/15-016743-1/2015	6	NA
MG964995	H5N2	05/20/2015	A/turkey/Iowa/15-016743-1/2015	7	M
MG964996	H5N2	05/20/2015	A/turkey/Iowa/15-016743-1/2015	8	NS
MG964997	H5N2	05/27/2015	A/turkey/Iowa/15-017224-1/2015	1	PB2
MG964998	H5N2	05/27/2015	A/turkey/Iowa/15-017224-1/2015	2	PB1
MG964999	H5N2	05/27/2015	A/turkey/Iowa/15-017224-1/2015	3	PA
MG965000	H5N2	05/27/2015	A/turkey/Iowa/15-017224-1/2015	4	HA
MG965001	H5N2	05/27/2015	A/turkey/Iowa/15-017224-1/2015	5	NP
MG965002	H5N2	05/27/2015	A/turkey/Iowa/15-017224-1/2015	6	NA
MG965003	H5N2	05/27/2015	A/turkey/Iowa/15-017224-1/2015	7	M
MG965004	H5N2	05/27/2015	A/turkey/Iowa/15-017224-1/2015	8	NS
MG965005	H5N2	05/28/2015	A/turkey/Iowa/15-017421-1/2015	1	PB2
MG965006	H5N2	05/28/2015	A/turkey/Iowa/15-017421-1/2015	2	PB1
MG965007	H5N2	05/28/2015	A/turkey/Iowa/15-017421-1/2015	3	PA
MG965008	H5N2	05/28/2015	A/turkey/Iowa/15-017421-1/2015	4	HA
MG965009	H5N2	05/28/2015	A/turkey/Iowa/15-017421-1/2015	5	NP
MG965010	H5N2	05/28/2015	A/turkey/Iowa/15-017421-1/2015	6	NA
MG965011	H5N2	05/28/2015	A/turkey/Iowa/15-017421-1/2015	7	M
MG965012	H5N2	05/28/2015	A/turkey/Iowa/15-017421-1/2015	8	NS
MG965013	H5N2	05/31/2015	A/turkey/Iowa/15-017556-1/2015	1	PB2
MG965014	H5N2	05/31/2015	A/turkey/Iowa/15-017556-1/2015	2	PB1
MG965015	H5N2	05/31/2015	A/turkey/Iowa/15-017556-1/2015	3	PA
MG965016	H5N2	05/31/2015	A/turkey/Iowa/15-017556-1/2015	4	HA
MG965017	H5N2	05/31/2015	A/turkey/Iowa/15-017556-1/2015	5	NP
MG965018	H5N2	05/31/2015	A/turkey/Iowa/15-017556-1/2015	6	NA
MG965019	H5N2	05/31/2015	A/turkey/Iowa/15-017556-1/2015	7	M
MG965020	H5N2	05/31/2015	A/turkey/Iowa/15-017556-1/2015	8	NS
MG965021	H5N2	05/29/2015	A/turkey/Iowa/15-017557-1/2015	1	PB2
MG965022	H5N2	05/29/2015	A/turkey/Iowa/15-017557-1/2015	2	PB1
MG965023	H5N2	05/29/2015	A/turkey/Iowa/15-017557-1/2015	3	PA
MG965024	H5N2	05/29/2015	A/turkey/Iowa/15-017557-1/2015	4	HA
MG965025	H5N2	05/29/2015	A/turkey/Iowa/15-017557-1/2015	5	NP
MG965026	H5N2	05/29/2015	A/turkey/Iowa/15-017557-1/2015	6	NA
MG965027	H5N2	05/29/2015	A/turkey/Iowa/15-017557-1/2015	7	M
MG965028	H5N2	05/29/2015	A/turkey/Iowa/15-017557-1/2015	8	NS
MG965029	H5N2	06/01/2015	A/turkey/Iowa/15-017655-1/2015	1	PB2
MG965030	H5N2	06/01/2015	A/turkey/Iowa/15-017655-1/2015	2	PB1
MG965031	H5N2	06/01/2015	A/turkey/Iowa/15-017655-1/2015	3	PA
MG965032	H5N2	06/01/2015	A/turkey/Iowa/15-017655-1/2015	4	HA
MG965033	H5N2	06/01/2015	A/turkey/Iowa/15-017655-1/2015	5	NP
MG965034	H5N2	06/01/2015	A/turkey/Iowa/15-017655-1/2015	6	NA
MG965035	H5N2	06/01/2015	A/turkey/Iowa/15-017655-1/2015	7	M
MG965036	H5N2	06/01/2015	A/turkey/Iowa/15-017655-1/2015	8	NS
MG965037	H5N2	06/02/2015	A/turkey/Iowa/15-017824-1/2015	1	PB2
MG965038	H5N2	06/02/2015	A/turkey/Iowa/15-017824-1/2015	2	PB1
MG965039	H5N2	06/02/2015	A/turkey/Iowa/15-017824-1/2015	3	PA
MG965040	H5N2	06/02/2015	A/turkey/Iowa/15-017824-1/2015	4	HA
MG965041	H5N2	06/02/2015	A/turkey/Iowa/15-017824-1/2015	5	NP
MG965042	H5N2	06/02/2015	A/turkey/Iowa/15-017824-1/2015	6	NA
MG965043	H5N2	06/02/2015	A/turkey/Iowa/15-017824-1/2015	7	M
MG965044	H5N2	06/02/2015	A/turkey/Iowa/15-017824-1/2015	8	NS
MG965045	H5N2	03/31/2015	A/turkey/Minnesota/15-010375-2/2015	1	PB2
MG965046	H5N2	03/31/2015	A/turkey/Minnesota/15-010375-2/2015	2	PB1
MG965047	H5N2	03/31/2015	A/turkey/Minnesota/15-010375-2/2015	3	PA
MG965048	H5N2	03/31/2015	A/turkey/Minnesota/15-010375-2/2015	4	HA
MG965049	H5N2	03/31/2015	A/turkey/Minnesota/15-010375-2/2015	5	NP
MG965050	H5N2	03/31/2015	A/turkey/Minnesota/15-010375-2/2015	6	NA
MG965051	H5N2	03/31/2015	A/turkey/Minnesota/15-010375-2/2015	7	M

MG965052	H5N2	03/31/2015	A/turkey/Minnesota/15-010375-2/2015	8	NS
MG965053	H5N2	03/30/2015	A/turkey/Minnesota/15-010560-1/2015	1	PB2
MG965054	H5N2	03/30/2015	A/turkey/Minnesota/15-010560-1/2015	2	PB1
MG965055	H5N2	03/30/2015	A/turkey/Minnesota/15-010560-1/2015	3	PA
MG965056	H5N2	03/30/2015	A/turkey/Minnesota/15-010560-1/2015	4	HA
MG965057	H5N2	03/30/2015	A/turkey/Minnesota/15-010560-1/2015	5	NP
MG965058	H5N2	03/30/2015	A/turkey/Minnesota/15-010560-1/2015	6	NA
MG965059	H5N2	03/30/2015	A/turkey/Minnesota/15-010560-1/2015	7	M
MG965060	H5N2	03/30/2015	A/turkey/Minnesota/15-010560-1/2015	8	NS
MG965061	H5N2	04/02/2015	A/turkey/Minnesota/15-010777-1/2015	1	PB2
MG965062	H5N2	04/02/2015	A/turkey/Minnesota/15-010777-1/2015	2	PB1
MG965063	H5N2	04/02/2015	A/turkey/Minnesota/15-010777-1/2015	3	PA
MG965064	H5N2	04/02/2015	A/turkey/Minnesota/15-010777-1/2015	4	HA
MG965065	H5N2	04/02/2015	A/turkey/Minnesota/15-010777-1/2015	5	NP
MG965066	H5N2	04/02/2015	A/turkey/Minnesota/15-010777-1/2015	6	NA
MG965067	H5N2	04/02/2015	A/turkey/Minnesota/15-010777-1/2015	7	M
MG965068	H5N2	04/02/2015	A/turkey/Minnesota/15-010777-1/2015	8	NS
MG965077	H5N2	04/05/2015	A/turkey/Minnesota/15-010915-1/2015	1	PB2
MG965078	H5N2	04/05/2015	A/turkey/Minnesota/15-010915-1/2015	2	PB1
MG965079	H5N2	04/05/2015	A/turkey/Minnesota/15-010915-1/2015	3	PA
MG965080	H5N2	04/05/2015	A/turkey/Minnesota/15-010915-1/2015	4	HA
MG965081	H5N2	04/05/2015	A/turkey/Minnesota/15-010915-1/2015	5	NP
MG965082	H5N2	04/05/2015	A/turkey/Minnesota/15-010915-1/2015	6	NA
MG965083	H5N2	04/05/2015	A/turkey/Minnesota/15-010915-1/2015	7	M
MG965084	H5N2	04/05/2015	A/turkey/Minnesota/15-010915-1/2015	8	NS
MG965085	H5N2	04/06/2015	A/turkey/Minnesota/15-011079-1/2015	1	PB2
MG965086	H5N2	04/06/2015	A/turkey/Minnesota/15-011079-1/2015	2	PB1
MG965087	H5N2	04/06/2015	A/turkey/Minnesota/15-011079-1/2015	3	PA
MG965088	H5N2	04/06/2015	A/turkey/Minnesota/15-011079-1/2015	4	HA
MG965089	H5N2	04/06/2015	A/turkey/Minnesota/15-011079-1/2015	5	NP
MG965090	H5N2	04/06/2015	A/turkey/Minnesota/15-011079-1/2015	6	NA
MG965091	H5N2	04/06/2015	A/turkey/Minnesota/15-011079-1/2015	7	M
MG965092	H5N2	04/06/2015	A/turkey/Minnesota/15-011079-1/2015	8	NS
MG965101	H5N2	2015	A/turkey/Minnesota/15-011202-1/2015	1	PB2
MG965102	H5N2	2015	A/turkey/Minnesota/15-011202-1/2015	2	PB1
MG965103	H5N2	2015	A/turkey/Minnesota/15-011202-1/2015	3	PA
MG965104	H5N2	2015	A/turkey/Minnesota/15-011202-1/2015	4	HA
MG965105	H5N2	2015	A/turkey/Minnesota/15-011202-1/2015	5	NP
MG965106	H5N2	2015	A/turkey/Minnesota/15-011202-1/2015	6	NA
MG965107	H5N2	2015	A/turkey/Minnesota/15-011202-1/2015	7	M
MG965108	H5N2	2015	A/turkey/Minnesota/15-011202-1/2015	8	NS
MG965109	H5N2	04/09/2015	A/turkey/Minnesota/15-011591-1/2015	1	PB2
MG965110	H5N2	04/09/2015	A/turkey/Minnesota/15-011591-1/2015	2	PB1
MG965111	H5N2	04/09/2015	A/turkey/Minnesota/15-011591-1/2015	3	PA
MG965112	H5N2	04/09/2015	A/turkey/Minnesota/15-011591-1/2015	4	HA
MG965113	H5N2	04/09/2015	A/turkey/Minnesota/15-011591-1/2015	5	NP
MG965114	H5N2	04/09/2015	A/turkey/Minnesota/15-011591-1/2015	6	NA
MG965115	H5N2	04/09/2015	A/turkey/Minnesota/15-011591-1/2015	7	M
MG965116	H5N2	04/09/2015	A/turkey/Minnesota/15-011591-1/2015	8	NS
MG965117	H5N2	2015	A/turkey/Minnesota/15-011593-1/2015	1	PB2
MG965118	H5N2	2015	A/turkey/Minnesota/15-011593-1/2015	2	PB1
MG965119	H5N2	2015	A/turkey/Minnesota/15-011593-1/2015	3	PA
MG965120	H5N2	2015	A/turkey/Minnesota/15-011593-1/2015	4	HA
MG965121	H5N2	2015	A/turkey/Minnesota/15-011593-1/2015	5	NP
MG965122	H5N2	2015	A/turkey/Minnesota/15-011593-1/2015	6	NA
MG965123	H5N2	2015	A/turkey/Minnesota/15-011593-1/2015	7	M
MG965124	H5N2	2015	A/turkey/Minnesota/15-011593-1/2015	8	NS
MG965125	H5N2	04/10/2015	A/turkey/Minnesota/15-011596-1/2015	1	PB2
MG965126	H5N2	04/10/2015	A/turkey/Minnesota/15-011596-1/2015	2	PB1
MG965127	H5N2	04/10/2015	A/turkey/Minnesota/15-011596-1/2015	3	PA
MG965128	H5N2	04/10/2015	A/turkey/Minnesota/15-011596-1/2015	4	HA
MG965129	H5N2	04/10/2015	A/turkey/Minnesota/15-011596-1/2015	5	NP
MG965130	H5N2	04/10/2015	A/turkey/Minnesota/15-011596-1/2015	6	NA
MG965131	H5N2	04/10/2015	A/turkey/Minnesota/15-011596-1/2015	7	M
MG965132	H5N2	04/10/2015	A/turkey/Minnesota/15-011596-1/2015	8	NS
MG965133	H5N2	04/10/2015	A/turkey/Minnesota/15-011602-1/2015	1	PB2
MG965134	H5N2	04/10/2015	A/turkey/Minnesota/15-011602-1/2015	2	PB1
MG965135	H5N2	04/10/2015	A/turkey/Minnesota/15-011602-1/2015	3	PA
MG965136	H5N2	04/10/2015	A/turkey/Minnesota/15-011602-1/2015	4	HA
MG965137	H5N2	04/10/2015	A/turkey/Minnesota/15-011602-1/2015	5	NP
MG965138	H5N2	04/10/2015	A/turkey/Minnesota/15-011602-1/2015	6	NA
MG965139	H5N2	04/10/2015	A/turkey/Minnesota/15-011602-1/2015	7	M
MG965140	H5N2	04/10/2015	A/turkey/Minnesota/15-011602-1/2015	8	NS
MG965141	H5N2	04/10/2015	A/turkey/Minnesota/15-011603-1/2015	1	PB2
MG965142	H5N2	04/10/2015	A/turkey/Minnesota/15-011603-1/2015	2	PB1
MG965143	H5N2	04/10/2015	A/turkey/Minnesota/15-011603-1/2015	3	PA
MG965144	H5N2	04/10/2015	A/turkey/Minnesota/15-011603-1/2015	4	HA
MG965145	H5N2	04/10/2015	A/turkey/Minnesota/15-011603-1/2015	5	NP
MG965146	H5N2	04/10/2015	A/turkey/Minnesota/15-011603-1/2015	6	NA
MG965147	H5N2	04/10/2015	A/turkey/Minnesota/15-011603-1/2015	7	M
MG965148	H5N2	04/10/2015	A/turkey/Minnesota/15-011603-1/2015	8	NS
MG965149	H5N2	04/12/2015	A/turkey/Minnesota/15-011656-1/2015	1	PB2
MG965150	H5N2	04/12/2015	A/turkey/Minnesota/15-011656-1/2015	2	PB1
MG965151	H5N2	04/12/2015	A/turkey/Minnesota/15-011656-1/2015	3	PA
MG965152	H5N2	04/12/2015	A/turkey/Minnesota/15-011656-1/2015	4	HA
MG965153	H5N2	04/12/2015	A/turkey/Minnesota/15-011656-1/2015	5	NP
MG965154	H5N2	04/12/2015	A/turkey/Minnesota/15-011656-1/2015	6	NA
MG965155	H5N2	04/12/2015	A/turkey/Minnesota/15-011656-1/2015	7	M
MG965156	H5N2	04/12/2015	A/turkey/Minnesota/15-011656-1/2015	8	NS
MG965157	H5N2	04/12/2015	A/turkey/Minnesota/15-011661-1/2015	1	PB2

MG965158	H5N2	04/12/2015	A/turkey/Minnesota/15-011661-1/2015	2	PB1
MG965159	H5N2	04/12/2015	A/turkey/Minnesota/15-011661-1/2015	3	PA
MG965160	H5N2	04/12/2015	A/turkey/Minnesota/15-011661-1/2015	4	HA
MG965161	H5N2	04/12/2015	A/turkey/Minnesota/15-011661-1/2015	5	NP
MG965162	H5N2	04/12/2015	A/turkey/Minnesota/15-011661-1/2015	6	NA
MG965163	H5N2	04/12/2015	A/turkey/Minnesota/15-011661-1/2015	7	M
MG965164	H5N2	04/12/2015	A/turkey/Minnesota/15-011661-1/2015	8	NS
MG965165	H5N2	04/11/2015	A/turkey/Minnesota/15-011666-1/2015	1	PB2
MG965166	H5N2	04/11/2015	A/turkey/Minnesota/15-011666-1/2015	2	PB1
MG965167	H5N2	04/11/2015	A/turkey/Minnesota/15-011666-1/2015	3	PA
MG965168	H5N2	04/11/2015	A/turkey/Minnesota/15-011666-1/2015	4	HA
MG965169	H5N2	04/11/2015	A/turkey/Minnesota/15-011666-1/2015	5	NP
MG965170	H5N2	04/11/2015	A/turkey/Minnesota/15-011666-1/2015	6	NA
MG965171	H5N2	04/11/2015	A/turkey/Minnesota/15-011666-1/2015	7	M
MG965172	H5N2	04/11/2015	A/turkey/Minnesota/15-011666-1/2015	8	NS
MG965173	H5N2	04/11/2015	A/turkey/Minnesota/15-011668-1/2015	1	PB2
MG965174	H5N2	04/11/2015	A/turkey/Minnesota/15-011668-1/2015	2	PB1
MG965175	H5N2	04/11/2015	A/turkey/Minnesota/15-011668-1/2015	3	PA
MG965176	H5N2	04/11/2015	A/turkey/Minnesota/15-011668-1/2015	4	HA
MG965177	H5N2	04/11/2015	A/turkey/Minnesota/15-011668-1/2015	5	NP
MG965178	H5N2	04/11/2015	A/turkey/Minnesota/15-011668-1/2015	6	NA
MG965179	H5N2	04/11/2015	A/turkey/Minnesota/15-011668-1/2015	7	M
MG965180	H5N2	04/11/2015	A/turkey/Minnesota/15-011668-1/2015	8	NS
MG965189	H5N2	04/13/2015	A/turkey/Minnesota/15-011833-1/2015	1	PB2
MG965190	H5N2	04/13/2015	A/turkey/Minnesota/15-011833-1/2015	2	PB1
MG965191	H5N2	04/13/2015	A/turkey/Minnesota/15-011833-1/2015	3	PA
MG965192	H5N2	04/13/2015	A/turkey/Minnesota/15-011833-1/2015	4	HA
MG965193	H5N2	04/13/2015	A/turkey/Minnesota/15-011833-1/2015	5	NP
MG965194	H5N2	04/13/2015	A/turkey/Minnesota/15-011833-1/2015	6	NA
MG965195	H5N2	04/13/2015	A/turkey/Minnesota/15-011833-1/2015	7	M
MG965196	H5N2	04/13/2015	A/turkey/Minnesota/15-011833-1/2015	8	NS
MG965197	H5N2	04/13/2015	A/turkey/Minnesota/15-011834-1/2015	1	PB2
MG965198	H5N2	04/13/2015	A/turkey/Minnesota/15-011834-1/2015	2	PB1
MG965199	H5N2	04/13/2015	A/turkey/Minnesota/15-011834-1/2015	3	PA
MG965200	H5N2	04/13/2015	A/turkey/Minnesota/15-011834-1/2015	4	HA
MG965201	H5N2	04/13/2015	A/turkey/Minnesota/15-011834-1/2015	5	NP
MG965202	H5N2	04/13/2015	A/turkey/Minnesota/15-011834-1/2015	6	NA
MG965203	H5N2	04/13/2015	A/turkey/Minnesota/15-011834-1/2015	7	M
MG965204	H5N2	04/13/2015	A/turkey/Minnesota/15-011834-1/2015	8	NS
MG965205	H5N2	04/13/2015	A/turkey/Minnesota/15-011849-1/2015	1	PB2
MG965206	H5N2	04/13/2015	A/turkey/Minnesota/15-011849-1/2015	2	PB1
MG965207	H5N2	04/13/2015	A/turkey/Minnesota/15-011849-1/2015	3	PA
MG965208	H5N2	04/13/2015	A/turkey/Minnesota/15-011849-1/2015	4	HA
MG965209	H5N2	04/13/2015	A/turkey/Minnesota/15-011849-1/2015	5	NP
MG965210	H5N2	04/13/2015	A/turkey/Minnesota/15-011849-1/2015	6	NA
MG965211	H5N2	04/13/2015	A/turkey/Minnesota/15-011849-1/2015	7	M
MG965212	H5N2	04/13/2015	A/turkey/Minnesota/15-011849-1/2015	8	NS
MG965213	H5N2	04/13/2015	A/turkey/Minnesota/15-011923-1/2015	1	PB2
MG965214	H5N2	04/13/2015	A/turkey/Minnesota/15-011923-1/2015	2	PB1
MG965215	H5N2	04/13/2015	A/turkey/Minnesota/15-011923-1/2015	3	PA
MG965216	H5N2	04/13/2015	A/turkey/Minnesota/15-011923-1/2015	4	HA
MG965217	H5N2	04/13/2015	A/turkey/Minnesota/15-011923-1/2015	5	NP
MG965218	H5N2	04/13/2015	A/turkey/Minnesota/15-011923-1/2015	6	NA
MG965219	H5N2	04/13/2015	A/turkey/Minnesota/15-011923-1/2015	7	M
MG965220	H5N2	04/13/2015	A/turkey/Minnesota/15-011923-1/2015	8	NS
MG965221	H5N2	04/15/2015	A/turkey/Minnesota/15-012289-2/2015	1	PB2
MG965222	H5N2	04/15/2015	A/turkey/Minnesota/15-012289-2/2015	2	PB1
MG965223	H5N2	04/15/2015	A/turkey/Minnesota/15-012289-2/2015	3	PA
MG965224	H5N2	04/15/2015	A/turkey/Minnesota/15-012289-2/2015	4	HA
MG965225	H5N2	04/15/2015	A/turkey/Minnesota/15-012289-2/2015	5	NP
MG965226	H5N2	04/15/2015	A/turkey/Minnesota/15-012289-2/2015	6	NA
MG965227	H5N2	04/15/2015	A/turkey/Minnesota/15-012289-2/2015	7	M
MG965228	H5N2	04/15/2015	A/turkey/Minnesota/15-012289-2/2015	8	NS
MG965229	H5N2	04/15/2015	A/turkey/Minnesota/15-012508-2/2015	1	PB2
MG965230	H5N2	04/15/2015	A/turkey/Minnesota/15-012508-2/2015	2	PB1
MG965231	H5N2	04/15/2015	A/turkey/Minnesota/15-012508-2/2015	3	PA
MG965232	H5N2	04/15/2015	A/turkey/Minnesota/15-012508-2/2015	4	HA
MG965233	H5N2	04/15/2015	A/turkey/Minnesota/15-012508-2/2015	5	NP
MG965234	H5N2	04/15/2015	A/turkey/Minnesota/15-012508-2/2015	6	NA
MG965235	H5N2	04/15/2015	A/turkey/Minnesota/15-012508-2/2015	7	M
MG965236	H5N2	04/15/2015	A/turkey/Minnesota/15-012508-2/2015	8	NS
MG965245	H5N2	04/15/2015	A/turkey/Minnesota/15-012510-4/2015	1	PB2
MG965246	H5N2	04/15/2015	A/turkey/Minnesota/15-012510-4/2015	2	PB1
MG965247	H5N2	04/15/2015	A/turkey/Minnesota/15-012510-4/2015	3	PA
MG965248	H5N2	04/15/2015	A/turkey/Minnesota/15-012510-4/2015	4	HA
MG965249	H5N2	04/15/2015	A/turkey/Minnesota/15-012510-4/2015	5	NP
MG965250	H5N2	04/15/2015	A/turkey/Minnesota/15-012510-4/2015	6	NA
MG965251	H5N2	04/15/2015	A/turkey/Minnesota/15-012510-4/2015	7	M
MG965252	H5N2	04/15/2015	A/turkey/Minnesota/15-012510-4/2015	8	NS
MG965261	H5N2	04/17/2015	A/turkey/Minnesota/15-012575-1/2015	1	PB2
MG965262	H5N2	04/17/2015	A/turkey/Minnesota/15-012575-1/2015	2	PB1
MG965263	H5N2	04/17/2015	A/turkey/Minnesota/15-012575-1/2015	3	PA
MG965264	H5N2	04/17/2015	A/turkey/Minnesota/15-012575-1/2015	4	HA
MG965265	H5N2	04/17/2015	A/turkey/Minnesota/15-012575-1/2015	5	NP
MG965266	H5N2	04/17/2015	A/turkey/Minnesota/15-012575-1/2015	6	NA
MG965267	H5N2	04/17/2015	A/turkey/Minnesota/15-012575-1/2015	7	M
MG965268	H5N2	04/17/2015	A/turkey/Minnesota/15-012575-1/2015	8	NS
MG965269	H5N2	04/16/2015	A/turkey/Minnesota/15-012576-1/2015	1	PB2
MG965270	H5N2	04/16/2015	A/turkey/Minnesota/15-012576-1/2015	2	PB1
MG965271	H5N2	04/16/2015	A/turkey/Minnesota/15-012576-1/2015	3	PA

MG965272	H5N2	04/16/2015	A/turkey/Minnesota/15-012576-1/2015	4	HA
MG965273	H5N2	04/16/2015	A/turkey/Minnesota/15-012576-1/2015	5	NP
MG965274	H5N2	04/16/2015	A/turkey/Minnesota/15-012576-1/2015	6	NA
MG965275	H5N2	04/16/2015	A/turkey/Minnesota/15-012576-1/2015	7	M
MG965276	H5N2	04/16/2015	A/turkey/Minnesota/15-012576-1/2015	8	NS
MG965277	H5N2	04/18/2015	A/turkey/Minnesota/15-012577-1/2015	1	PB2
MG965278	H5N2	04/18/2015	A/turkey/Minnesota/15-012577-1/2015	2	PB1
MG965279	H5N2	04/18/2015	A/turkey/Minnesota/15-012577-1/2015	3	PA
MG965280	H5N2	04/18/2015	A/turkey/Minnesota/15-012577-1/2015	4	HA
MG965281	H5N2	04/18/2015	A/turkey/Minnesota/15-012577-1/2015	5	NP
MG965282	H5N2	04/18/2015	A/turkey/Minnesota/15-012577-1/2015	6	NA
MG965283	H5N2	04/18/2015	A/turkey/Minnesota/15-012577-1/2015	7	M
MG965284	H5N2	04/18/2015	A/turkey/Minnesota/15-012577-1/2015	8	NS
MG965285	H5N2	04/18/2015	A/turkey/Minnesota/15-012578-4/2015	1	PB2
MG965286	H5N2	04/18/2015	A/turkey/Minnesota/15-012578-4/2015	2	PB1
MG965287	H5N2	04/18/2015	A/turkey/Minnesota/15-012578-4/2015	3	PA
MG965288	H5N2	04/18/2015	A/turkey/Minnesota/15-012578-4/2015	4	HA
MG965289	H5N2	04/18/2015	A/turkey/Minnesota/15-012578-4/2015	5	NP
MG965290	H5N2	04/18/2015	A/turkey/Minnesota/15-012578-4/2015	6	NA
MG965291	H5N2	04/18/2015	A/turkey/Minnesota/15-012578-4/2015	7	M
MG965292	H5N2	04/18/2015	A/turkey/Minnesota/15-012578-4/2015	8	NS
MG965293	H5N2	04/18/2015	A/turkey/Minnesota/15-012580-4/2015	1	PB2
MG965294	H5N2	04/18/2015	A/turkey/Minnesota/15-012580-4/2015	2	PB1
MG965295	H5N2	04/18/2015	A/turkey/Minnesota/15-012580-4/2015	3	PA
MG965296	H5N2	04/18/2015	A/turkey/Minnesota/15-012580-4/2015	4	HA
MG965297	H5N2	04/18/2015	A/turkey/Minnesota/15-012580-4/2015	5	NP
MG965298	H5N2	04/18/2015	A/turkey/Minnesota/15-012580-4/2015	6	NA
MG965299	H5N2	04/18/2015	A/turkey/Minnesota/15-012580-4/2015	7	M
MG965300	H5N2	04/18/2015	A/turkey/Minnesota/15-012580-4/2015	8	NS
MG965301	H5N2	04/17/2015	A/turkey/Minnesota/15-012583-1/2015	1	PB2
MG965302	H5N2	04/17/2015	A/turkey/Minnesota/15-012583-1/2015	2	PB1
MG965303	H5N2	04/17/2015	A/turkey/Minnesota/15-012583-1/2015	3	PA
MG965304	H5N2	04/17/2015	A/turkey/Minnesota/15-012583-1/2015	4	HA
MG965305	H5N2	04/17/2015	A/turkey/Minnesota/15-012583-1/2015	5	NP
MG965306	H5N2	04/17/2015	A/turkey/Minnesota/15-012583-1/2015	6	NA
MG965307	H5N2	04/17/2015	A/turkey/Minnesota/15-012583-1/2015	7	M
MG965308	H5N2	04/17/2015	A/turkey/Minnesota/15-012583-1/2015	8	NS
MG965309	H5N2	04/17/2015	A/turkey/Minnesota/15-012584-1/2015	1	PB2
MG965310	H5N2	04/17/2015	A/turkey/Minnesota/15-012584-1/2015	2	PB1
MG965311	H5N2	04/17/2015	A/turkey/Minnesota/15-012584-1/2015	3	PA
MG965312	H5N2	04/17/2015	A/turkey/Minnesota/15-012584-1/2015	4	HA
MG965313	H5N2	04/17/2015	A/turkey/Minnesota/15-012584-1/2015	5	NP
MG965314	H5N2	04/17/2015	A/turkey/Minnesota/15-012584-1/2015	6	NA
MG965315	H5N2	04/17/2015	A/turkey/Minnesota/15-012584-1/2015	7	M
MG965316	H5N2	04/17/2015	A/turkey/Minnesota/15-012584-1/2015	8	NS
MG965325	H5N2	04/18/2015	A/turkey/Minnesota/15-012658-1/2015	1	PB2
MG965326	H5N2	04/18/2015	A/turkey/Minnesota/15-012658-1/2015	2	PB1
MG965327	H5N2	04/18/2015	A/turkey/Minnesota/15-012658-1/2015	3	PA
MG965328	H5N2	04/18/2015	A/turkey/Minnesota/15-012658-1/2015	4	HA
MG965329	H5N2	04/18/2015	A/turkey/Minnesota/15-012658-1/2015	5	NP
MG965330	H5N2	04/18/2015	A/turkey/Minnesota/15-012658-1/2015	6	NA
MG965331	H5N2	04/18/2015	A/turkey/Minnesota/15-012658-1/2015	7	M
MG965332	H5N2	04/18/2015	A/turkey/Minnesota/15-012658-1/2015	8	NS
MG965333	H5N2	04/18/2015	A/turkey/Minnesota/15-012659-2/2015	1	PB2
MG965334	H5N2	04/18/2015	A/turkey/Minnesota/15-012659-2/2015	2	PB1
MG965335	H5N2	04/18/2015	A/turkey/Minnesota/15-012659-2/2015	3	PA
MG965336	H5N2	04/18/2015	A/turkey/Minnesota/15-012659-2/2015	4	HA
MG965337	H5N2	04/18/2015	A/turkey/Minnesota/15-012659-2/2015	5	NP
MG965338	H5N2	04/18/2015	A/turkey/Minnesota/15-012659-2/2015	6	NA
MG965339	H5N2	04/18/2015	A/turkey/Minnesota/15-012659-2/2015	7	M
MG965340	H5N2	04/18/2015	A/turkey/Minnesota/15-012659-2/2015	8	NS
MG965341	H5N2	04/19/2015	A/turkey/Minnesota/15-012661-1/2015	1	PB2
MG965342	H5N2	04/19/2015	A/turkey/Minnesota/15-012661-1/2015	2	PB1
MG965343	H5N2	04/19/2015	A/turkey/Minnesota/15-012661-1/2015	3	PA
MG965344	H5N2	04/19/2015	A/turkey/Minnesota/15-012661-1/2015	4	HA
MG965345	H5N2	04/19/2015	A/turkey/Minnesota/15-012661-1/2015	5	NP
MG965346	H5N2	04/19/2015	A/turkey/Minnesota/15-012661-1/2015	6	NA
MG965347	H5N2	04/19/2015	A/turkey/Minnesota/15-012661-1/2015	7	M
MG965348	H5N2	04/19/2015	A/turkey/Minnesota/15-012661-1/2015	8	NS
MG965349	H5N2	04/19/2015	A/turkey/Minnesota/15-012663-1/2015	1	PB2
MG965350	H5N2	04/19/2015	A/turkey/Minnesota/15-012663-1/2015	2	PB1
MG965351	H5N2	04/19/2015	A/turkey/Minnesota/15-012663-1/2015	3	PA
MG965352	H5N2	04/19/2015	A/turkey/Minnesota/15-012663-1/2015	4	HA
MG965353	H5N2	04/19/2015	A/turkey/Minnesota/15-012663-1/2015	5	NP
MG965354	H5N2	04/19/2015	A/turkey/Minnesota/15-012663-1/2015	6	NA
MG965355	H5N2	04/19/2015	A/turkey/Minnesota/15-012663-1/2015	7	M
MG965356	H5N2	04/19/2015	A/turkey/Minnesota/15-012663-1/2015	8	NS
MG965357	H5N2	04/19/2015	A/turkey/Minnesota/15-012666-1/2015	1	PB2
MG965358	H5N2	04/19/2015	A/turkey/Minnesota/15-012666-1/2015	2	PB1
MG965359	H5N2	04/19/2015	A/turkey/Minnesota/15-012666-1/2015	3	PA
MG965360	H5N2	04/19/2015	A/turkey/Minnesota/15-012666-1/2015	4	HA
MG965361	H5N2	04/19/2015	A/turkey/Minnesota/15-012666-1/2015	5	NP
MG965362	H5N2	04/19/2015	A/turkey/Minnesota/15-012666-1/2015	6	NA
MG965363	H5N2	04/19/2015	A/turkey/Minnesota/15-012666-1/2015	7	M
MG965364	H5N2	04/19/2015	A/turkey/Minnesota/15-012666-1/2015	8	NS
MG965365	H5N2	04/20/2015	A/turkey/Minnesota/15-012887-1/2015	1	PB2
MG965366	H5N2	04/20/2015	A/turkey/Minnesota/15-012887-1/2015	2	PB1
MG965367	H5N2	04/20/2015	A/turkey/Minnesota/15-012887-1/2015	3	PA
MG965368	H5N2	04/20/2015	A/turkey/Minnesota/15-012887-1/2015	4	HA
MG965369	H5N2	04/20/2015	A/turkey/Minnesota/15-012887-1/2015	5	NP

MG965370	H5N2	04/20/2015	A/turkey/Minnesota/15-012887-1/2015	6	NA
MG965371	H5N2	04/20/2015	A/turkey/Minnesota/15-012887-1/2015	7	M
MG965372	H5N2	04/20/2015	A/turkey/Minnesota/15-012887-1/2015	8	NS
MG965373	H5N2	04/20/2015	A/turkey/Minnesota/15-012888-1/2015	1	PB2
MG965374	H5N2	04/20/2015	A/turkey/Minnesota/15-012888-1/2015	2	PB1
MG965375	H5N2	04/20/2015	A/turkey/Minnesota/15-012888-1/2015	3	PA
MG965376	H5N2	04/20/2015	A/turkey/Minnesota/15-012888-1/2015	4	HA
MG965377	H5N2	04/20/2015	A/turkey/Minnesota/15-012888-1/2015	5	NP
MG965378	H5N2	04/20/2015	A/turkey/Minnesota/15-012888-1/2015	6	NA
MG965379	H5N2	04/20/2015	A/turkey/Minnesota/15-012888-1/2015	7	M
MG965380	H5N2	04/20/2015	A/turkey/Minnesota/15-012888-1/2015	8	NS
MG965381	H5N2	04/20/2015	A/turkey/Minnesota/15-012889-1/2015	1	PB2
MG965382	H5N2	04/20/2015	A/turkey/Minnesota/15-012889-1/2015	2	PB1
MG965383	H5N2	04/20/2015	A/turkey/Minnesota/15-012889-1/2015	3	PA
MG965384	H5N2	04/20/2015	A/turkey/Minnesota/15-012889-1/2015	4	HA
MG965385	H5N2	04/20/2015	A/turkey/Minnesota/15-012889-1/2015	5	NP
MG965386	H5N2	04/20/2015	A/turkey/Minnesota/15-012889-1/2015	6	NA
MG965387	H5N2	04/20/2015	A/turkey/Minnesota/15-012889-1/2015	7	M
MG965388	H5N2	04/20/2015	A/turkey/Minnesota/15-012889-1/2015	8	NS
MG965389	H5N2	04/20/2015	A/turkey/Minnesota/15-013050-1/2015	1	PB2
MG965390	H5N2	04/20/2015	A/turkey/Minnesota/15-013050-1/2015	2	PB1
MG965391	H5N2	04/20/2015	A/turkey/Minnesota/15-013050-1/2015	3	PA
MG965392	H5N2	04/20/2015	A/turkey/Minnesota/15-013050-1/2015	4	HA
MG965393	H5N2	04/20/2015	A/turkey/Minnesota/15-013050-1/2015	5	NP
MG965394	H5N2	04/20/2015	A/turkey/Minnesota/15-013050-1/2015	6	NA
MG965395	H5N2	04/20/2015	A/turkey/Minnesota/15-013050-1/2015	7	M
MG965396	H5N2	04/20/2015	A/turkey/Minnesota/15-013050-1/2015	8	NS
MG965397	H5N2	04/21/2015	A/turkey/Minnesota/15-013051-1/2015	1	PB2
MG965398	H5N2	04/21/2015	A/turkey/Minnesota/15-013051-1/2015	2	PB1
MG965399	H5N2	04/21/2015	A/turkey/Minnesota/15-013051-1/2015	3	PA
MG965400	H5N2	04/21/2015	A/turkey/Minnesota/15-013051-1/2015	4	HA
MG965401	H5N2	04/21/2015	A/turkey/Minnesota/15-013051-1/2015	5	NP
MG965402	H5N2	04/21/2015	A/turkey/Minnesota/15-013051-1/2015	6	NA
MG965403	H5N2	04/21/2015	A/turkey/Minnesota/15-013051-1/2015	7	M
MG965404	H5N2	04/21/2015	A/turkey/Minnesota/15-013051-1/2015	8	NS
MG965405	H5N2	04/21/2015	A/turkey/Minnesota/15-013052-1/2015	1	PB2
MG965406	H5N2	04/21/2015	A/turkey/Minnesota/15-013052-1/2015	2	PB1
MG965407	H5N2	04/21/2015	A/turkey/Minnesota/15-013052-1/2015	3	PA
MG965408	H5N2	04/21/2015	A/turkey/Minnesota/15-013052-1/2015	4	HA
MG965409	H5N2	04/21/2015	A/turkey/Minnesota/15-013052-1/2015	5	NP
MG965410	H5N2	04/21/2015	A/turkey/Minnesota/15-013052-1/2015	6	NA
MG965411	H5N2	04/21/2015	A/turkey/Minnesota/15-013052-1/2015	7	M
MG965412	H5N2	04/21/2015	A/turkey/Minnesota/15-013052-1/2015	8	NS
MG965413	H5N2	04/22/2015	A/turkey/Minnesota/15-013181-1/2015	1	PB2
MG965414	H5N2	04/22/2015	A/turkey/Minnesota/15-013181-1/2015	2	PB1
MG965415	H5N2	04/22/2015	A/turkey/Minnesota/15-013181-1/2015	3	PA
MG965416	H5N2	04/22/2015	A/turkey/Minnesota/15-013181-1/2015	4	HA
MG965417	H5N2	04/22/2015	A/turkey/Minnesota/15-013181-1/2015	5	NP
MG965418	H5N2	04/22/2015	A/turkey/Minnesota/15-013181-1/2015	6	NA
MG965419	H5N2	04/22/2015	A/turkey/Minnesota/15-013181-1/2015	7	M
MG965420	H5N2	04/22/2015	A/turkey/Minnesota/15-013181-1/2015	8	NS
MG965421	H5N2	04/24/2015	A/turkey/Minnesota/15-013391-1/2015	1	PB2
MG965422	H5N2	04/24/2015	A/turkey/Minnesota/15-013391-1/2015	2	PB1
MG965423	H5N2	04/24/2015	A/turkey/Minnesota/15-013391-1/2015	3	PA
MG965424	H5N2	04/24/2015	A/turkey/Minnesota/15-013391-1/2015	4	HA
MG965425	H5N2	04/24/2015	A/turkey/Minnesota/15-013391-1/2015	5	NP
MG965426	H5N2	04/24/2015	A/turkey/Minnesota/15-013391-1/2015	6	NA
MG965427	H5N2	04/24/2015	A/turkey/Minnesota/15-013391-1/2015	7	M
MG965428	H5N2	04/24/2015	A/turkey/Minnesota/15-013391-1/2015	8	NS
MG965429	H5N2	04/24/2015	A/turkey/Minnesota/15-013392-1/2015	1	PB2
MG965430	H5N2	04/24/2015	A/turkey/Minnesota/15-013392-1/2015	2	PB1
MG965431	H5N2	04/24/2015	A/turkey/Minnesota/15-013392-1/2015	3	PA
MG965432	H5N2	04/24/2015	A/turkey/Minnesota/15-013392-1/2015	4	HA
MG965433	H5N2	04/24/2015	A/turkey/Minnesota/15-013392-1/2015	5	NP
MG965434	H5N2	04/24/2015	A/turkey/Minnesota/15-013392-1/2015	6	NA
MG965435	H5N2	04/24/2015	A/turkey/Minnesota/15-013392-1/2015	7	M
MG965436	H5N2	04/24/2015	A/turkey/Minnesota/15-013392-1/2015	8	NS
MG965437	H5N2	04/24/2015	A/turkey/Minnesota/15-013393-1/2015	1	PB2
MG965438	H5N2	04/24/2015	A/turkey/Minnesota/15-013393-1/2015	2	PB1
MG965439	H5N2	04/24/2015	A/turkey/Minnesota/15-013393-1/2015	3	PA
MG965440	H5N2	04/24/2015	A/turkey/Minnesota/15-013393-1/2015	4	HA
MG965441	H5N2	04/24/2015	A/turkey/Minnesota/15-013393-1/2015	5	NP
MG965442	H5N2	04/24/2015	A/turkey/Minnesota/15-013393-1/2015	6	NA
MG965443	H5N2	04/24/2015	A/turkey/Minnesota/15-013393-1/2015	7	M
MG965444	H5N2	04/24/2015	A/turkey/Minnesota/15-013393-1/2015	8	NS
MG965445	H5N2	04/24/2015	A/turkey/Minnesota/15-013395-1/2015	1	PB2
MG965446	H5N2	04/24/2015	A/turkey/Minnesota/15-013395-1/2015	2	PB1
MG965447	H5N2	04/24/2015	A/turkey/Minnesota/15-013395-1/2015	3	PA
MG965448	H5N2	04/24/2015	A/turkey/Minnesota/15-013395-1/2015	4	HA
MG965449	H5N2	04/24/2015	A/turkey/Minnesota/15-013395-1/2015	5	NP
MG965450	H5N2	04/24/2015	A/turkey/Minnesota/15-013395-1/2015	6	NA
MG965451	H5N2	04/24/2015	A/turkey/Minnesota/15-013395-1/2015	7	M
MG965452	H5N2	04/24/2015	A/turkey/Minnesota/15-013395-1/2015	8	NS
MG965453	H5N2	04/23/2015	A/turkey/Minnesota/15-013398-1/2015	1	PB2
MG965454	H5N2	04/23/2015	A/turkey/Minnesota/15-013398-1/2015	2	PB1
MG965455	H5N2	04/23/2015	A/turkey/Minnesota/15-013398-1/2015	3	PA
MG965456	H5N2	04/23/2015	A/turkey/Minnesota/15-013398-1/2015	4	HA
MG965457	H5N2	04/23/2015	A/turkey/Minnesota/15-013398-1/2015	5	NP
MG965458	H5N2	04/23/2015	A/turkey/Minnesota/15-013398-1/2015	6	NA
MG965459	H5N2	04/23/2015	A/turkey/Minnesota/15-013398-1/2015	7	M

MG965460	H5N2	04/23/2015	A/turkey/Minnesota/15-013398-1/2015	8	NS
MG965461	H5N2	04/23/2015	A/turkey/Minnesota/15-013399-1/2015	1	PB2
MG965462	H5N2	04/23/2015	A/turkey/Minnesota/15-013399-1/2015	2	PB1
MG965463	H5N2	04/23/2015	A/turkey/Minnesota/15-013399-1/2015	3	PA
MG965464	H5N2	04/23/2015	A/turkey/Minnesota/15-013399-1/2015	4	HA
MG965465	H5N2	04/23/2015	A/turkey/Minnesota/15-013399-1/2015	5	NP
MG965466	H5N2	04/23/2015	A/turkey/Minnesota/15-013399-1/2015	6	NA
MG965467	H5N2	04/23/2015	A/turkey/Minnesota/15-013399-1/2015	7	M
MG965468	H5N2	04/23/2015	A/turkey/Minnesota/15-013399-1/2015	8	NS
MG965469	H5N2	04/23/2015	A/turkey/Minnesota/15-013401-1/2015	1	PB2
MG965470	H5N2	04/23/2015	A/turkey/Minnesota/15-013401-1/2015	2	PB1
MG965471	H5N2	04/23/2015	A/turkey/Minnesota/15-013401-1/2015	3	PA
MG965472	H5N2	04/23/2015	A/turkey/Minnesota/15-013401-1/2015	4	HA
MG965473	H5N2	04/23/2015	A/turkey/Minnesota/15-013401-1/2015	5	NP
MG965474	H5N2	04/23/2015	A/turkey/Minnesota/15-013401-1/2015	6	NA
MG965475	H5N2	04/23/2015	A/turkey/Minnesota/15-013401-1/2015	7	M
MG965476	H5N2	04/23/2015	A/turkey/Minnesota/15-013401-1/2015	8	NS
MG965477	H5N2	04/25/2015	A/turkey/Minnesota/15-013534-1/2015	1	PB2
MG965478	H5N2	04/25/2015	A/turkey/Minnesota/15-013534-1/2015	2	PB1
MG965479	H5N2	04/25/2015	A/turkey/Minnesota/15-013534-1/2015	3	PA
MG965480	H5N2	04/25/2015	A/turkey/Minnesota/15-013534-1/2015	4	HA
MG965481	H5N2	04/25/2015	A/turkey/Minnesota/15-013534-1/2015	5	NP
MG965482	H5N2	04/25/2015	A/turkey/Minnesota/15-013534-1/2015	6	NA
MG965483	H5N2	04/25/2015	A/turkey/Minnesota/15-013534-1/2015	7	M
MG965484	H5N2	04/25/2015	A/turkey/Minnesota/15-013534-1/2015	8	NS
MG965485	H5N2	04/27/2015	A/turkey/Minnesota/15-013755-2/2015	1	PB2
MG965486	H5N2	04/27/2015	A/turkey/Minnesota/15-013755-2/2015	2	PB1
MG965487	H5N2	04/27/2015	A/turkey/Minnesota/15-013755-2/2015	3	PA
MG965488	H5N2	04/27/2015	A/turkey/Minnesota/15-013755-2/2015	4	HA
MG965489	H5N2	04/27/2015	A/turkey/Minnesota/15-013755-2/2015	5	NP
MG965490	H5N2	04/27/2015	A/turkey/Minnesota/15-013755-2/2015	6	NA
MG965491	H5N2	04/27/2015	A/turkey/Minnesota/15-013755-2/2015	7	M
MG965492	H5N2	04/27/2015	A/turkey/Minnesota/15-013755-2/2015	8	NS
MG965493	H5N2	04/28/2015	A/turkey/Minnesota/15-013895-1/2015	1	PB2
MG965494	H5N2	04/28/2015	A/turkey/Minnesota/15-013895-1/2015	2	PB1
MG965495	H5N2	04/28/2015	A/turkey/Minnesota/15-013895-1/2015	3	PA
MG965496	H5N2	04/28/2015	A/turkey/Minnesota/15-013895-1/2015	4	HA
MG965497	H5N2	04/28/2015	A/turkey/Minnesota/15-013895-1/2015	5	NP
MG965498	H5N2	04/28/2015	A/turkey/Minnesota/15-013895-1/2015	6	NA
MG965499	H5N2	04/28/2015	A/turkey/Minnesota/15-013895-1/2015	7	M
MG965500	H5N2	04/28/2015	A/turkey/Minnesota/15-013895-1/2015	8	NS
MG965501	H5N2	04/28/2015	A/turkey/Minnesota/15-013896-1/2015	1	PB2
MG965502	H5N2	04/28/2015	A/turkey/Minnesota/15-013896-1/2015	2	PB1
MG965503	H5N2	04/28/2015	A/turkey/Minnesota/15-013896-1/2015	3	PA
MG965504	H5N2	04/28/2015	A/turkey/Minnesota/15-013896-1/2015	4	HA
MG965505	H5N2	04/28/2015	A/turkey/Minnesota/15-013896-1/2015	5	NP
MG965506	H5N2	04/28/2015	A/turkey/Minnesota/15-013896-1/2015	6	NA
MG965507	H5N2	04/28/2015	A/turkey/Minnesota/15-013896-1/2015	7	M
MG965508	H5N2	04/28/2015	A/turkey/Minnesota/15-013896-1/2015	8	NS
MG965509	H5N2	04/29/2015	A/turkey/Minnesota/15-014095-1/2015	1	PB2
MG965510	H5N2	04/29/2015	A/turkey/Minnesota/15-014095-1/2015	2	PB1
MG965511	H5N2	04/29/2015	A/turkey/Minnesota/15-014095-1/2015	3	PA
MG965512	H5N2	04/29/2015	A/turkey/Minnesota/15-014095-1/2015	4	HA
MG965513	H5N2	04/29/2015	A/turkey/Minnesota/15-014095-1/2015	5	NP
MG965514	H5N2	04/29/2015	A/turkey/Minnesota/15-014095-1/2015	6	NA
MG965515	H5N2	04/29/2015	A/turkey/Minnesota/15-014095-1/2015	7	M
MG965516	H5N2	04/29/2015	A/turkey/Minnesota/15-014095-1/2015	8	NS
MG965517	H5N2	04/29/2015	A/turkey/Minnesota/15-014097-1/2015	1	PB2
MG965518	H5N2	04/29/2015	A/turkey/Minnesota/15-014097-1/2015	2	PB1
MG965519	H5N2	04/29/2015	A/turkey/Minnesota/15-014097-1/2015	3	PA
MG965520	H5N2	04/29/2015	A/turkey/Minnesota/15-014097-1/2015	4	HA
MG965521	H5N2	04/29/2015	A/turkey/Minnesota/15-014097-1/2015	5	NP
MG965522	H5N2	04/29/2015	A/turkey/Minnesota/15-014097-1/2015	6	NA
MG965523	H5N2	04/29/2015	A/turkey/Minnesota/15-014097-1/2015	7	M
MG965524	H5N2	04/29/2015	A/turkey/Minnesota/15-014097-1/2015	8	NS
MG965525	H5N2	04/29/2015	A/turkey/Minnesota/15-014098-1/2015	1	PB2
MG965526	H5N2	04/29/2015	A/turkey/Minnesota/15-014098-1/2015	2	PB1
MG965527	H5N2	04/29/2015	A/turkey/Minnesota/15-014098-1/2015	3	PA
MG965528	H5N2	04/29/2015	A/turkey/Minnesota/15-014098-1/2015	4	HA
MG965529	H5N2	04/29/2015	A/turkey/Minnesota/15-014098-1/2015	5	NP
MG965530	H5N2	04/29/2015	A/turkey/Minnesota/15-014098-1/2015	6	NA
MG965531	H5N2	04/29/2015	A/turkey/Minnesota/15-014098-1/2015	7	M
MG965532	H5N2	04/29/2015	A/turkey/Minnesota/15-014098-1/2015	8	NS
MG965533	H5N2	04/29/2015	A/turkey/Minnesota/15-014110-1/2015	1	PB2
MG965534	H5N2	04/29/2015	A/turkey/Minnesota/15-014110-1/2015	2	PB1
MG965535	H5N2	04/29/2015	A/turkey/Minnesota/15-014110-1/2015	3	PA
MG965536	H5N2	04/29/2015	A/turkey/Minnesota/15-014110-1/2015	4	HA
MG965537	H5N2	04/29/2015	A/turkey/Minnesota/15-014110-1/2015	5	NP
MG965538	H5N2	04/29/2015	A/turkey/Minnesota/15-014110-1/2015	6	NA
MG965539	H5N2	04/29/2015	A/turkey/Minnesota/15-014110-1/2015	7	M
MG965540	H5N2	04/29/2015	A/turkey/Minnesota/15-014110-1/2015	8	NS
MG965541	H5N2	04/29/2015	A/turkey/Minnesota/15-014111-1/2015	1	PB2
MG965542	H5N2	04/29/2015	A/turkey/Minnesota/15-014111-1/2015	2	PB1
MG965543	H5N2	04/29/2015	A/turkey/Minnesota/15-014111-1/2015	3	PA
MG965544	H5N2	04/29/2015	A/turkey/Minnesota/15-014111-1/2015	4	HA
MG965545	H5N2	04/29/2015	A/turkey/Minnesota/15-014111-1/2015	5	NP
MG965546	H5N2	04/29/2015	A/turkey/Minnesota/15-014111-1/2015	6	NA
MG965547	H5N2	04/29/2015	A/turkey/Minnesota/15-014111-1/2015	7	M
MG965548	H5N2	04/29/2015	A/turkey/Minnesota/15-014111-1/2015	8	NS
MG965549	H5N2	04/30/2015	A/turkey/Minnesota/15-014293-1/2015	1	PB2

MG965550	H5N2	04/30/2015	A/turkey/Minnesota/15-014293-1/2015	2	PB1
MG965551	H5N2	04/30/2015	A/turkey/Minnesota/15-014293-1/2015	3	PA
MG965552	H5N2	04/30/2015	A/turkey/Minnesota/15-014293-1/2015	4	HA
MG965553	H5N2	04/30/2015	A/turkey/Minnesota/15-014293-1/2015	5	NP
MG965554	H5N2	04/30/2015	A/turkey/Minnesota/15-014293-1/2015	6	NA
MG965555	H5N2	04/30/2015	A/turkey/Minnesota/15-014293-1/2015	7	M
MG965556	H5N2	04/30/2015	A/turkey/Minnesota/15-014293-1/2015	8	NS
MG965557	H5N2	05/01/2015	A/turkey/Minnesota/15-014299-1/2015	1	PB2
MG965558	H5N2	05/01/2015	A/turkey/Minnesota/15-014299-1/2015	2	PB1
MG965559	H5N2	05/01/2015	A/turkey/Minnesota/15-014299-1/2015	3	PA
MG965560	H5N2	05/01/2015	A/turkey/Minnesota/15-014299-1/2015	4	HA
MG965561	H5N2	05/01/2015	A/turkey/Minnesota/15-014299-1/2015	5	NP
MG965562	H5N2	05/01/2015	A/turkey/Minnesota/15-014299-1/2015	6	NA
MG965563	H5N2	05/01/2015	A/turkey/Minnesota/15-014299-1/2015	7	M
MG965564	H5N2	05/01/2015	A/turkey/Minnesota/15-014299-1/2015	8	NS
MG965565	H5N2	05/02/2015	A/turkey/Minnesota/15-014301-1/2015	1	PB2
MG965566	H5N2	05/02/2015	A/turkey/Minnesota/15-014301-1/2015	2	PB1
MG965567	H5N2	05/02/2015	A/turkey/Minnesota/15-014301-1/2015	3	PA
MG965568	H5N2	05/02/2015	A/turkey/Minnesota/15-014301-1/2015	4	HA
MG965569	H5N2	05/02/2015	A/turkey/Minnesota/15-014301-1/2015	5	NP
MG965570	H5N2	05/02/2015	A/turkey/Minnesota/15-014301-1/2015	6	NA
MG965571	H5N2	05/02/2015	A/turkey/Minnesota/15-014301-1/2015	7	M
MG965572	H5N2	05/02/2015	A/turkey/Minnesota/15-014301-1/2015	8	NS
MG965573	H5N2	04/30/2015	A/turkey/Minnesota/15-014310-1/2015	1	PB2
MG965574	H5N2	04/30/2015	A/turkey/Minnesota/15-014310-1/2015	2	PB1
MG965575	H5N2	04/30/2015	A/turkey/Minnesota/15-014310-1/2015	3	PA
MG965576	H5N2	04/30/2015	A/turkey/Minnesota/15-014310-1/2015	4	HA
MG965577	H5N2	04/30/2015	A/turkey/Minnesota/15-014310-1/2015	5	NP
MG965578	H5N2	04/30/2015	A/turkey/Minnesota/15-014310-1/2015	6	NA
MG965579	H5N2	04/30/2015	A/turkey/Minnesota/15-014310-1/2015	7	M
MG965580	H5N2	04/30/2015	A/turkey/Minnesota/15-014310-1/2015	8	NS
MG965581	H5N2	04/30/2015	A/turkey/Minnesota/15-014311-1/2015	1	PB2
MG965582	H5N2	04/30/2015	A/turkey/Minnesota/15-014311-1/2015	2	PB1
MG965583	H5N2	04/30/2015	A/turkey/Minnesota/15-014311-1/2015	3	PA
MG965584	H5N2	04/30/2015	A/turkey/Minnesota/15-014311-1/2015	4	HA
MG965585	H5N2	04/30/2015	A/turkey/Minnesota/15-014311-1/2015	5	NP
MG965586	H5N2	04/30/2015	A/turkey/Minnesota/15-014311-1/2015	6	NA
MG965587	H5N2	04/30/2015	A/turkey/Minnesota/15-014311-1/2015	7	M
MG965588	H5N2	04/30/2015	A/turkey/Minnesota/15-014311-1/2015	8	NS
MG965589	H5N2	05/02/2015	A/turkey/Minnesota/15-014381-1/2015	1	PB2
MG965590	H5N2	05/02/2015	A/turkey/Minnesota/15-014381-1/2015	2	PB1
MG965591	H5N2	05/02/2015	A/turkey/Minnesota/15-014381-1/2015	3	PA
MG965592	H5N2	05/02/2015	A/turkey/Minnesota/15-014381-1/2015	4	HA
MG965593	H5N2	05/02/2015	A/turkey/Minnesota/15-014381-1/2015	5	NP
MG965594	H5N2	05/02/2015	A/turkey/Minnesota/15-014381-1/2015	6	NA
MG965595	H5N2	05/02/2015	A/turkey/Minnesota/15-014381-1/2015	7	M
MG965596	H5N2	05/02/2015	A/turkey/Minnesota/15-014381-1/2015	8	NS
MG965597	H5N2	05/06/2015	A/turkey/Minnesota/15-014850-1/2015	1	PB2
MG965598	H5N2	05/06/2015	A/turkey/Minnesota/15-014850-1/2015	2	PB1
MG965599	H5N2	05/06/2015	A/turkey/Minnesota/15-014850-1/2015	3	PA
MG965600	H5N2	05/06/2015	A/turkey/Minnesota/15-014850-1/2015	4	HA
MG965601	H5N2	05/06/2015	A/turkey/Minnesota/15-014850-1/2015	5	NP
MG965602	H5N2	05/06/2015	A/turkey/Minnesota/15-014850-1/2015	6	NA
MG965603	H5N2	05/06/2015	A/turkey/Minnesota/15-014850-1/2015	7	M
MG965604	H5N2	05/06/2015	A/turkey/Minnesota/15-014850-1/2015	8	NS
MG965605	H5N2	05/06/2015	A/turkey/Minnesota/15-014851-1/2015	1	PB2
MG965606	H5N2	05/06/2015	A/turkey/Minnesota/15-014851-1/2015	2	PB1
MG965607	H5N2	05/06/2015	A/turkey/Minnesota/15-014851-1/2015	3	PA
MG965608	H5N2	05/06/2015	A/turkey/Minnesota/15-014851-1/2015	4	HA
MG965609	H5N2	05/06/2015	A/turkey/Minnesota/15-014851-1/2015	5	NP
MG965610	H5N2	05/06/2015	A/turkey/Minnesota/15-014851-1/2015	6	NA
MG965611	H5N2	05/06/2015	A/turkey/Minnesota/15-014851-1/2015	7	M
MG965612	H5N2	05/06/2015	A/turkey/Minnesota/15-014851-1/2015	8	NS
MG965613	H5N2	05/06/2015	A/turkey/Minnesota/15-014972-1/2015	1	PB2
MG965614	H5N2	05/06/2015	A/turkey/Minnesota/15-014972-1/2015	2	PB1
MG965615	H5N2	05/06/2015	A/turkey/Minnesota/15-014972-1/2015	3	PA
MG965616	H5N2	05/06/2015	A/turkey/Minnesota/15-014972-1/2015	4	HA
MG965617	H5N2	05/06/2015	A/turkey/Minnesota/15-014972-1/2015	5	NP
MG965618	H5N2	05/06/2015	A/turkey/Minnesota/15-014972-1/2015	6	NA
MG965619	H5N2	05/06/2015	A/turkey/Minnesota/15-014972-1/2015	7	M
MG965620	H5N2	05/06/2015	A/turkey/Minnesota/15-014972-1/2015	8	NS
MG965621	H5N2	05/14/2015	A/turkey/Minnesota/15-016050-1/2015	1	PB2
MG965622	H5N2	05/14/2015	A/turkey/Minnesota/15-016050-1/2015	2	PB1
MG965623	H5N2	05/14/2015	A/turkey/Minnesota/15-016050-1/2015	3	PA
MG965624	H5N2	05/14/2015	A/turkey/Minnesota/15-016050-1/2015	4	HA
MG965625	H5N2	05/14/2015	A/turkey/Minnesota/15-016050-1/2015	5	NP
MG965626	H5N2	05/14/2015	A/turkey/Minnesota/15-016050-1/2015	6	NA
MG965627	H5N2	05/14/2015	A/turkey/Minnesota/15-016050-1/2015	7	M
MG965628	H5N2	05/14/2015	A/turkey/Minnesota/15-016050-1/2015	8	NS
MG965629	H5N2	05/24/2015	A/turkey/Minnesota/15-016876-1/2015	1	PB2
MG965630	H5N2	05/24/2015	A/turkey/Minnesota/15-016876-1/2015	2	PB1
MG965631	H5N2	05/24/2015	A/turkey/Minnesota/15-016876-1/2015	3	PA
MG965632	H5N2	05/24/2015	A/turkey/Minnesota/15-016876-1/2015	4	HA
MG965633	H5N2	05/24/2015	A/turkey/Minnesota/15-016876-1/2015	5	NP
MG965634	H5N2	05/24/2015	A/turkey/Minnesota/15-016876-1/2015	6	NA
MG965635	H5N2	05/24/2015	A/turkey/Minnesota/15-016876-1/2015	7	M
MG965636	H5N2	05/24/2015	A/turkey/Minnesota/15-016876-1/2015	8	NS
MG965637	H5N2	05/24/2015	A/turkey/Minnesota/15-016877-1/2015	1	PB2
MG965638	H5N2	05/24/2015	A/turkey/Minnesota/15-016877-1/2015	2	PB1
MG965639	H5N2	05/24/2015	A/turkey/Minnesota/15-016877-1/2015	3	PA

MG965640	H5N2	05/24/2015	A/turkey/Minnesota/15-016877-1/2015	4	HA
MG965641	H5N2	05/24/2015	A/turkey/Minnesota/15-016877-1/2015	5	NP
MG965642	H5N2	05/24/2015	A/turkey/Minnesota/15-016877-1/2015	6	NA
MG965643	H5N2	05/24/2015	A/turkey/Minnesota/15-016877-1/2015	7	M
MG965644	H5N2	05/24/2015	A/turkey/Minnesota/15-016877-1/2015	8	NS
MG965645	H5N2	05/25/2015	A/turkey/Minnesota/15-017039-1/2015	1	PB2
MG965646	H5N2	05/25/2015	A/turkey/Minnesota/15-017039-1/2015	2	PB1
MG965647	H5N2	05/25/2015	A/turkey/Minnesota/15-017039-1/2015	3	PA
MG965648	H5N2	05/25/2015	A/turkey/Minnesota/15-017039-1/2015	4	HA
MG965649	H5N2	05/25/2015	A/turkey/Minnesota/15-017039-1/2015	5	NP
MG965650	H5N2	05/25/2015	A/turkey/Minnesota/15-017039-1/2015	6	NA
MG965651	H5N2	05/25/2015	A/turkey/Minnesota/15-017039-1/2015	7	M
MG965652	H5N2	05/25/2015	A/turkey/Minnesota/15-017039-1/2015	8	NS
MG965653	H5N2	05/25/2015	A/turkey/Minnesota/15-017040-1/2015	1	PB2
MG965654	H5N2	05/25/2015	A/turkey/Minnesota/15-017040-1/2015	2	PB1
MG965655	H5N2	05/25/2015	A/turkey/Minnesota/15-017040-1/2015	3	PA
MG965656	H5N2	05/25/2015	A/turkey/Minnesota/15-017040-1/2015	4	HA
MG965657	H5N2	05/25/2015	A/turkey/Minnesota/15-017040-1/2015	5	NP
MG965658	H5N2	05/25/2015	A/turkey/Minnesota/15-017040-1/2015	6	NA
MG965659	H5N2	05/25/2015	A/turkey/Minnesota/15-017040-1/2015	7	M
MG965660	H5N2	05/25/2015	A/turkey/Minnesota/15-017040-1/2015	8	NS
MG965661	H5N2	05/25/2015	A/turkey/Minnesota/15-017044-1/2015	1	PB2
MG965662	H5N2	05/25/2015	A/turkey/Minnesota/15-017044-1/2015	2	PB1
MG965663	H5N2	05/25/2015	A/turkey/Minnesota/15-017044-1/2015	3	PA
MG965664	H5N2	05/25/2015	A/turkey/Minnesota/15-017044-1/2015	4	HA
MG965665	H5N2	05/25/2015	A/turkey/Minnesota/15-017044-1/2015	5	NP
MG965666	H5N2	05/25/2015	A/turkey/Minnesota/15-017044-1/2015	6	NA
MG965667	H5N2	05/25/2015	A/turkey/Minnesota/15-017044-1/2015	7	M
MG965668	H5N2	05/25/2015	A/turkey/Minnesota/15-017044-1/2015	8	NS
MG965669	H5N2	05/24/2015	A/turkey/Minnesota/15-017046-1/2015	1	PB2
MG965670	H5N2	05/24/2015	A/turkey/Minnesota/15-017046-1/2015	2	PB1
MG965671	H5N2	05/24/2015	A/turkey/Minnesota/15-017046-1/2015	3	PA
MG965672	H5N2	05/24/2015	A/turkey/Minnesota/15-017046-1/2015	4	HA
MG965673	H5N2	05/24/2015	A/turkey/Minnesota/15-017046-1/2015	5	NP
MG965674	H5N2	05/24/2015	A/turkey/Minnesota/15-017046-1/2015	6	NA
MG965675	H5N2	05/24/2015	A/turkey/Minnesota/15-017046-1/2015	7	M
MG965676	H5N2	05/24/2015	A/turkey/Minnesota/15-017046-1/2015	8	NS
MG965677	H5N2	05/26/2015	A/turkey/Minnesota/15-017217-1/2015	1	PB2
MG965678	H5N2	05/26/2015	A/turkey/Minnesota/15-017217-1/2015	2	PB1
MG965679	H5N2	05/26/2015	A/turkey/Minnesota/15-017217-1/2015	3	PA
MG965680	H5N2	05/26/2015	A/turkey/Minnesota/15-017217-1/2015	4	HA
MG965681	H5N2	05/26/2015	A/turkey/Minnesota/15-017217-1/2015	5	NP
MG965682	H5N2	05/26/2015	A/turkey/Minnesota/15-017217-1/2015	6	NA
MG965683	H5N2	05/26/2015	A/turkey/Minnesota/15-017217-1/2015	7	M
MG965684	H5N2	05/26/2015	A/turkey/Minnesota/15-017217-1/2015	8	NS
MG965685	H5N2	05/26/2015	A/turkey/Minnesota/15-017218-1/2015	1	PB2
MG965686	H5N2	05/26/2015	A/turkey/Minnesota/15-017218-1/2015	2	PB1
MG965687	H5N2	05/26/2015	A/turkey/Minnesota/15-017218-1/2015	3	PA
MG965688	H5N2	05/26/2015	A/turkey/Minnesota/15-017218-1/2015	4	HA
MG965689	H5N2	05/26/2015	A/turkey/Minnesota/15-017218-1/2015	5	NP
MG965690	H5N2	05/26/2015	A/turkey/Minnesota/15-017218-1/2015	6	NA
MG965691	H5N2	05/26/2015	A/turkey/Minnesota/15-017218-1/2015	7	M
MG965692	H5N2	05/26/2015	A/turkey/Minnesota/15-017218-1/2015	8	NS
MG965693	H5N2	05/26/2015	A/turkey/Minnesota/15-017219-1/2015	1	PB2
MG965694	H5N2	05/26/2015	A/turkey/Minnesota/15-017219-1/2015	2	PB1
MG965695	H5N2	05/26/2015	A/turkey/Minnesota/15-017219-1/2015	3	PA
MG965696	H5N2	05/26/2015	A/turkey/Minnesota/15-017219-1/2015	4	HA
MG965697	H5N2	05/26/2015	A/turkey/Minnesota/15-017219-1/2015	5	NP
MG965698	H5N2	05/26/2015	A/turkey/Minnesota/15-017219-1/2015	6	NA
MG965699	H5N2	05/26/2015	A/turkey/Minnesota/15-017219-1/2015	7	M
MG965700	H5N2	05/26/2015	A/turkey/Minnesota/15-017219-1/2015	8	NS
MG965701	H5N2	05/27/2015	A/turkey/Minnesota/15-017409-1/2015	1	PB2
MG965702	H5N2	05/27/2015	A/turkey/Minnesota/15-017409-1/2015	2	PB1
MG965703	H5N2	05/27/2015	A/turkey/Minnesota/15-017409-1/2015	3	PA
MG965704	H5N2	05/27/2015	A/turkey/Minnesota/15-017409-1/2015	4	HA
MG965705	H5N2	05/27/2015	A/turkey/Minnesota/15-017409-1/2015	5	NP
MG965706	H5N2	05/27/2015	A/turkey/Minnesota/15-017409-1/2015	6	NA
MG965707	H5N2	05/27/2015	A/turkey/Minnesota/15-017409-1/2015	7	M
MG965708	H5N2	05/27/2015	A/turkey/Minnesota/15-017409-1/2015	8	NS
MG965709	H5N2	05/27/2015	A/turkey/Minnesota/15-017410-1/2015	1	PB2
MG965710	H5N2	05/27/2015	A/turkey/Minnesota/15-017410-1/2015	2	PB1
MG965711	H5N2	05/27/2015	A/turkey/Minnesota/15-017410-1/2015	3	PA
MG965712	H5N2	05/27/2015	A/turkey/Minnesota/15-017410-1/2015	4	HA
MG965713	H5N2	05/27/2015	A/turkey/Minnesota/15-017410-1/2015	5	NP
MG965714	H5N2	05/27/2015	A/turkey/Minnesota/15-017410-1/2015	6	NA
MG965715	H5N2	05/27/2015	A/turkey/Minnesota/15-017410-1/2015	7	M
MG965716	H5N2	05/27/2015	A/turkey/Minnesota/15-017410-1/2015	8	NS
MG965717	H5N2	05/28/2015	A/turkey/Minnesota/15-017540-1/2015	1	PB2
MG965718	H5N2	05/28/2015	A/turkey/Minnesota/15-017540-1/2015	2	PB1
MG965719	H5N2	05/28/2015	A/turkey/Minnesota/15-017540-1/2015	3	PA
MG965720	H5N2	05/28/2015	A/turkey/Minnesota/15-017540-1/2015	4	HA
MG965721	H5N2	05/28/2015	A/turkey/Minnesota/15-017540-1/2015	5	NP
MG965722	H5N2	05/28/2015	A/turkey/Minnesota/15-017540-1/2015	6	NA
MG965723	H5N2	05/28/2015	A/turkey/Minnesota/15-017540-1/2015	7	M
MG965724	H5N2	05/28/2015	A/turkey/Minnesota/15-017540-1/2015	8	NS
MG965725	H5N2	05/31/2015	A/turkey/Minnesota/15-017647-1/2015	1	PB2
MG965726	H5N2	05/31/2015	A/turkey/Minnesota/15-017647-1/2015	2	PB1
MG965727	H5N2	05/31/2015	A/turkey/Minnesota/15-017647-1/2015	3	PA
MG965728	H5N2	05/31/2015	A/turkey/Minnesota/15-017647-1/2015	4	HA
MG965729	H5N2	05/31/2015	A/turkey/Minnesota/15-017647-1/2015	5	NP

MG965730	H5N2	05/31/2015	A/turkey/Minnesota/15-017647-1/2015	6	NA
MG965731	H5N2	05/31/2015	A/turkey/Minnesota/15-017647-1/2015	7	M
MG965732	H5N2	05/31/2015	A/turkey/Minnesota/15-017647-1/2015	8	NS
MG965733	H5N2	05/30/2015	A/turkey/Minnesota/15-017649-1/2015	1	PB2
MG965734	H5N2	05/30/2015	A/turkey/Minnesota/15-017649-1/2015	2	PB1
MG965735	H5N2	05/30/2015	A/turkey/Minnesota/15-017649-1/2015	3	PA
MG965736	H5N2	05/30/2015	A/turkey/Minnesota/15-017649-1/2015	4	HA
MG965737	H5N2	05/30/2015	A/turkey/Minnesota/15-017649-1/2015	5	NP
MG965738	H5N2	05/30/2015	A/turkey/Minnesota/15-017649-1/2015	6	NA
MG965739	H5N2	05/30/2015	A/turkey/Minnesota/15-017649-1/2015	7	M
MG965740	H5N2	05/30/2015	A/turkey/Minnesota/15-017649-1/2015	8	NS
MG965741	H5N2	06/01/2015	A/turkey/Minnesota/15-017814-1/2015	1	PB2
MG965742	H5N2	06/01/2015	A/turkey/Minnesota/15-017814-1/2015	2	PB1
MG965743	H5N2	06/01/2015	A/turkey/Minnesota/15-017814-1/2015	3	PA
MG965744	H5N2	06/01/2015	A/turkey/Minnesota/15-017814-1/2015	4	HA
MG965745	H5N2	06/01/2015	A/turkey/Minnesota/15-017814-1/2015	5	NP
MG965746	H5N2	06/01/2015	A/turkey/Minnesota/15-017814-1/2015	6	NA
MG965747	H5N2	06/01/2015	A/turkey/Minnesota/15-017814-1/2015	7	M
MG965748	H5N2	06/01/2015	A/turkey/Minnesota/15-017814-1/2015	8	NS
MG965749	H5N2	06/02/2015	A/turkey/Minnesota/15-017989-1/2015	1	PB2
MG965750	H5N2	06/02/2015	A/turkey/Minnesota/15-017989-1/2015	2	PB1
MG965751	H5N2	06/02/2015	A/turkey/Minnesota/15-017989-1/2015	3	PA
MG965752	H5N2	06/02/2015	A/turkey/Minnesota/15-017989-1/2015	4	HA
MG965753	H5N2	06/02/2015	A/turkey/Minnesota/15-017989-1/2015	5	NP
MG965754	H5N2	06/02/2015	A/turkey/Minnesota/15-017989-1/2015	6	NA
MG965755	H5N2	06/02/2015	A/turkey/Minnesota/15-017989-1/2015	7	M
MG965756	H5N2	06/02/2015	A/turkey/Minnesota/15-017989-1/2015	8	NS
MG965757	H5N2	06/03/2015	A/turkey/Minnesota/15-018201-1/2015	1	PB2
MG965758	H5N2	06/03/2015	A/turkey/Minnesota/15-018201-1/2015	2	PB1
MG965759	H5N2	06/03/2015	A/turkey/Minnesota/15-018201-1/2015	3	PA
MG965760	H5N2	06/03/2015	A/turkey/Minnesota/15-018201-1/2015	4	HA
MG965761	H5N2	06/03/2015	A/turkey/Minnesota/15-018201-1/2015	5	NP
MG965762	H5N2	06/03/2015	A/turkey/Minnesota/15-018201-1/2015	6	NA
MG965763	H5N2	06/03/2015	A/turkey/Minnesota/15-018201-1/2015	7	M
MG965764	H5N2	06/03/2015	A/turkey/Minnesota/15-018201-1/2015	8	NS
MG965773	H5N2	04/06/2015	A/turkey/South Dakota/15-011089-3/2015	1	PB2
MG965774	H5N2	04/06/2015	A/turkey/South Dakota/15-011089-3/2015	2	PB1
MG965775	H5N2	04/06/2015	A/turkey/South Dakota/15-011089-3/2015	3	PA
MG965776	H5N2	04/06/2015	A/turkey/South Dakota/15-011089-3/2015	4	HA
MG965777	H5N2	04/06/2015	A/turkey/South Dakota/15-011089-3/2015	5	NP
MG965778	H5N2	04/06/2015	A/turkey/South Dakota/15-011089-3/2015	6	NA
MG965779	H5N2	04/06/2015	A/turkey/South Dakota/15-011089-3/2015	7	M
MG965780	H5N2	04/06/2015	A/turkey/South Dakota/15-011089-3/2015	8	NS
MG965805	H5N2	05/09/2015	A/turkey/South Dakota/15-015102-5/2015	1	PB2
MG965806	H5N2	05/09/2015	A/turkey/South Dakota/15-015102-5/2015	2	PB1
MG965807	H5N2	05/09/2015	A/turkey/South Dakota/15-015102-5/2015	3	PA
MG965808	H5N2	05/09/2015	A/turkey/South Dakota/15-015102-5/2015	4	HA
MG965809	H5N2	05/09/2015	A/turkey/South Dakota/15-015102-5/2015	5	NP
MG965810	H5N2	05/09/2015	A/turkey/South Dakota/15-015102-5/2015	6	NA
MG965811	H5N2	05/09/2015	A/turkey/South Dakota/15-015102-5/2015	7	M
MG965812	H5N2	05/09/2015	A/turkey/South Dakota/15-015102-5/2015	8	NS
MG965813	H5N2	05/09/2015	A/turkey/South Dakota/15-015103-1/2015	1	PB2
MG965814	H5N2	05/09/2015	A/turkey/South Dakota/15-015103-1/2015	2	PB1
MG965815	H5N2	05/09/2015	A/turkey/South Dakota/15-015103-1/2015	3	PA
MG965816	H5N2	05/09/2015	A/turkey/South Dakota/15-015103-1/2015	4	HA
MG965817	H5N2	05/09/2015	A/turkey/South Dakota/15-015103-1/2015	5	NP
MG965818	H5N2	05/09/2015	A/turkey/South Dakota/15-015103-1/2015	6	NA
MG965819	H5N2	05/09/2015	A/turkey/South Dakota/15-015103-1/2015	7	M
MG965820	H5N2	05/09/2015	A/turkey/South Dakota/15-015103-1/2015	8	NS
MG965821	H5N2	05/27/2015	A/turkey/South Dakota/15-017406-1/2015	1	PB2
MG965822	H5N2	05/27/2015	A/turkey/South Dakota/15-017406-1/2015	2	PB1
MG965823	H5N2	05/27/2015	A/turkey/South Dakota/15-017406-1/2015	3	PA
MG965824	H5N2	05/27/2015	A/turkey/South Dakota/15-017406-1/2015	4	HA
MG965825	H5N2	05/27/2015	A/turkey/South Dakota/15-017406-1/2015	5	NP
MG965826	H5N2	05/27/2015	A/turkey/South Dakota/15-017406-1/2015	6	NA
MG965827	H5N2	05/27/2015	A/turkey/South Dakota/15-017406-1/2015	7	M
MG965828	H5N2	05/27/2015	A/turkey/South Dakota/15-017406-1/2015	8	NS
MG965829	H5N2	04/20/2015	A/turkey/Wisconsin/15-012886-1/2015	1	PB2
MG965830	H5N2	04/20/2015	A/turkey/Wisconsin/15-012886-1/2015	2	PB1
MG965831	H5N2	04/20/2015	A/turkey/Wisconsin/15-012886-1/2015	3	PA
MG965832	H5N2	04/20/2015	A/turkey/Wisconsin/15-012886-1/2015	4	HA
MG965833	H5N2	04/20/2015	A/turkey/Wisconsin/15-012886-1/2015	5	NP
MG965834	H5N2	04/20/2015	A/turkey/Wisconsin/15-012886-1/2015	6	NA
MG965835	H5N2	04/20/2015	A/turkey/Wisconsin/15-012886-1/2015	7	M
MG965836	H5N2	04/20/2015	A/turkey/Wisconsin/15-012886-1/2015	8	NS
MG965837	H5N2	04/22/2015	A/turkey/Wisconsin/15-013180-1/2015	1	PB2
MG965838	H5N2	04/22/2015	A/turkey/Wisconsin/15-013180-1/2015	2	PB1
MG965839	H5N2	04/22/2015	A/turkey/Wisconsin/15-013180-1/2015	3	PA
MG965840	H5N2	04/22/2015	A/turkey/Wisconsin/15-013180-1/2015	4	HA
MG965841	H5N2	04/22/2015	A/turkey/Wisconsin/15-013180-1/2015	5	NP
MG965842	H5N2	04/22/2015	A/turkey/Wisconsin/15-013180-1/2015	6	NA
MG965843	H5N2	04/22/2015	A/turkey/Wisconsin/15-013180-1/2015	7	M
MG965844	H5N2	04/22/2015	A/turkey/Wisconsin/15-013180-1/2015	8	NS
MG965845	H5N2	04/27/2015	A/turkey/Wisconsin/15-013771-1/2015	1	PB2
MG965846	H5N2	04/27/2015	A/turkey/Wisconsin/15-013771-1/2015	2	PB1
MG965847	H5N2	04/27/2015	A/turkey/Wisconsin/15-013771-1/2015	3	PA
MG965848	H5N2	04/27/2015	A/turkey/Wisconsin/15-013771-1/2015	4	HA
MG965849	H5N2	04/27/2015	A/turkey/Wisconsin/15-013771-1/2015	5	NP
MG965850	H5N2	04/27/2015	A/turkey/Wisconsin/15-013771-1/2015	6	NA
MG965851	H5N2	04/27/2015	A/turkey/Wisconsin/15-013771-1/2015	7	M

MG965852	H5N2	04/27/2015	A/turkey/Wisconsin/15-013771-1/2015	8	NS
MG965853	H5N2	05/01/2015	A/turkey/Wisconsin/15-014296-1/2015	1	PB2
MG965854	H5N2	05/01/2015	A/turkey/Wisconsin/15-014296-1/2015	2	PB1
MG965855	H5N2	05/01/2015	A/turkey/Wisconsin/15-014296-1/2015	3	PA
MG965856	H5N2	05/01/2015	A/turkey/Wisconsin/15-014296-1/2015	4	HA
MG965857	H5N2	05/01/2015	A/turkey/Wisconsin/15-014296-1/2015	5	NP
MG965858	H5N2	05/01/2015	A/turkey/Wisconsin/15-014296-1/2015	6	NA
MG965859	H5N2	05/01/2015	A/turkey/Wisconsin/15-014296-1/2015	7	M
MG965860	H5N2	05/01/2015	A/turkey/Wisconsin/15-014296-1/2015	8	NS
MG965861	H5N2	05/02/2015	A/turkey/Wisconsin/15-014298-1/2015	1	PB2
MG965862	H5N2	05/02/2015	A/turkey/Wisconsin/15-014298-1/2015	2	PB1
MG965863	H5N2	05/02/2015	A/turkey/Wisconsin/15-014298-1/2015	3	PA
MG965864	H5N2	05/02/2015	A/turkey/Wisconsin/15-014298-1/2015	4	HA
MG965865	H5N2	05/02/2015	A/turkey/Wisconsin/15-014298-1/2015	5	NP
MG965866	H5N2	05/02/2015	A/turkey/Wisconsin/15-014298-1/2015	6	NA
MG965867	H5N2	05/02/2015	A/turkey/Wisconsin/15-014298-1/2015	7	M
MG965868	H5N2	05/02/2015	A/turkey/Wisconsin/15-014298-1/2015	8	NS
