# Supplementary material for: Agricultural and geographic factors shaped the North American 2015 highly pathogenic avian influenza H5N2 outbreak
Source: PLoS Pathog. 2020 Jan 21;16(1):e1007857. doi: 10.1371/journal.ppat.1007857 (PMC7004387; doi:10.1371/journal.ppat.1007857)
Supplement: S11 Table — All parameters were specified to be log normally distributed with a lower bound of 0. Mean (M) and standard deviation (S) are specified in the log distributed space. Initial values are in real space. (PDF) [file ppat.1007857.s012.pdf]

Supplemental Table S11. Prior settings of the compartmental coalescent models. All parameters were specified to be log normally distributed with a lower bound of 0. Mean (M) and standard deviation (S) are specified in the log distributed space. Initial values are in real space.

		Initial Value	M	S
Model 1	β	0.2	1.6	1.0
	γ	31.7	3.5	0.2
	Initial S	200	5.5	1.0
	Initial I	1	0	1.5
Model 2	β	0.2	1.6	1.0
	η	1	0	1.5
	γ	31.7	3.5	0.2
	Initial S	200	5.5	1.0
	Initial I	1	0	1.5
	U size	10	2.3	1.5
Model 3	β_T	0.2	1.6	1.0
	β_C	0.2	1.6	1.0
	β_{TC}	0.2	1.6	1.0
	β_{CT}	0.2	1.6	1.0
	γ_T	42.9	3.8	0.2
	γ_C	17.9	2.9	0.25
	Initial S_T	100	5.5	1.0
	Initial S_C	100	5.5	1.0
	Initial T	1	0	1.5
	Initial C	1	0	1.5
Model 4	β_T	0.2	1.6	1.0
	β_C	0.2	1.6	1.0
	β_{TC}	0.2	1.6	1.0
	β_{CT}	0.2	1.6	1.0
	η_T	1	0	1.5
	η_C	1	0	1.5
	γ_T	42.9	3.8	0.2
	γ_C	17.9	2.9	0.25
	Initial S_T	100	5.5	1.0
	Initial S_C	100	5.5	1.0
	Initial T	1	0	1.5
	Initial C	1	0	1.5
	U size	10	2.3	1.5
