# Supplementary figures and images for: Agricultural and geographic factors shaped the North American 2015 highly pathogenic avian influenza H5N2 outbreak
Source: PLoS Pathog. 2020 Jan 21;16(1):e1007857. doi: 10.1371/journal.ppat.1007857 (PMC7004387; doi:10.1371/journal.ppat.1007857)

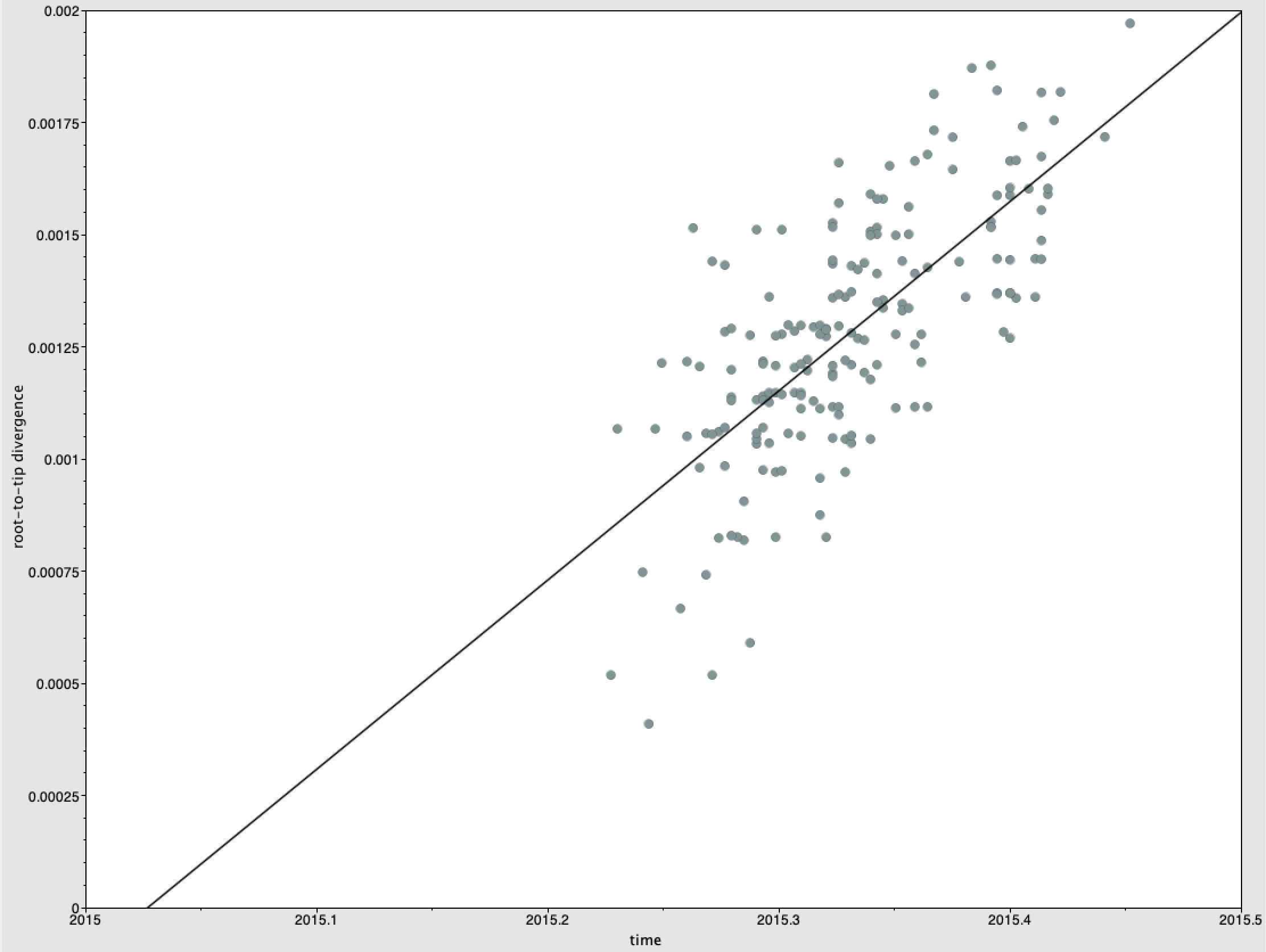

Supplement: S1 Fig — (PNG) [file ppat.1007857.s013.png]

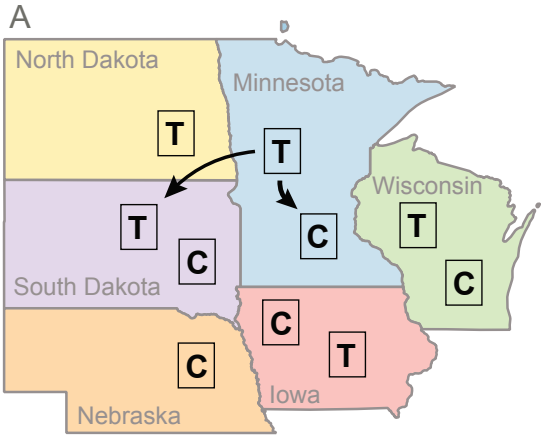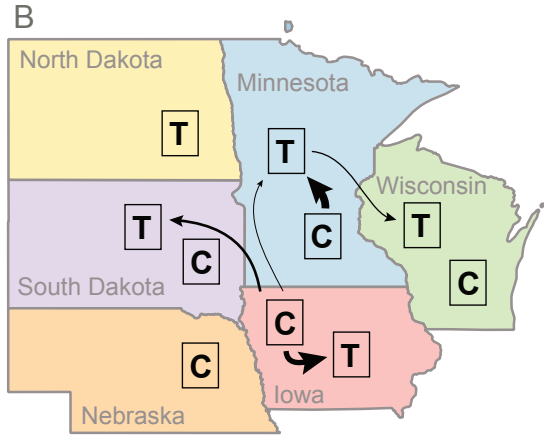

Supplement: S2 Fig — Epochs were defined as before (A) and after (B) April 10, 2015. Counties with only turkey cases (turkey exclusive; T) were grouped separately from counties with at least one layer chicken case (mixed poultry; C). Arrows represent transition rates with strong support (Bayes factor > 10) with arrow thickness proportional to the magnitude of transition rate. (PDF) [file ppat.1007857.s014.pdf]

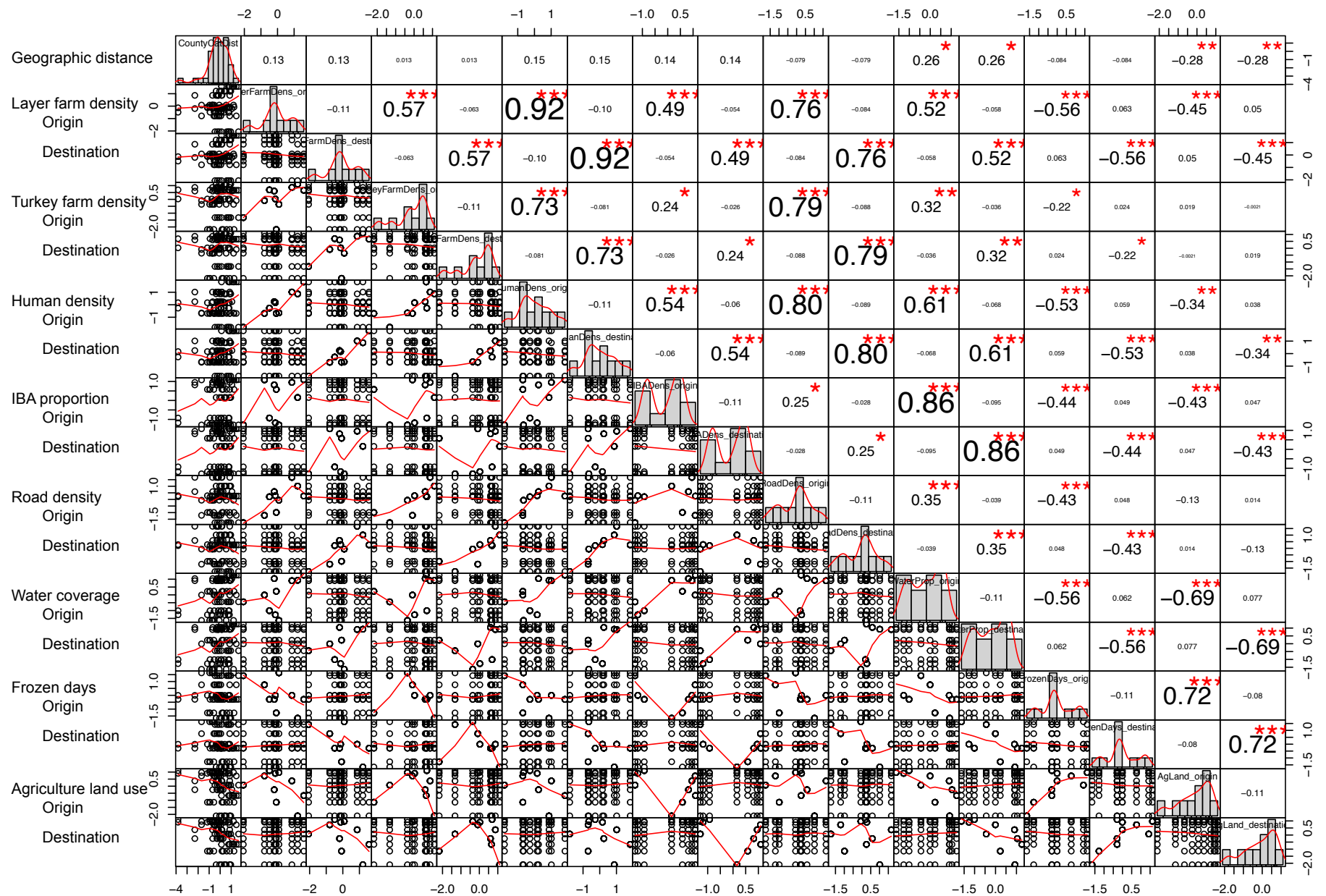

Supplement: S3 Fig — Variables have been log-transformed and standardized. Lower half of the matrix represents scatterplots between pairs of variables. Upper half of the matrix communicate the Pearson correlation coefficient. Level of statistical significance (p = 0.05, p = 0.01, p = 0.001) is denoted by asterisks (*, **, and ***, respectively). (PDF) [file ppat.1007857.s015.pdf]
